# Quinobenzothiazine–AZT Hybrids Linked via 1,2,3-Triazole: Rational Design, Synthesis, and Biological Evaluation as Anticancer Agents

**DOI:** 10.3390/ijms27125562

**Published:** 2026-06-19

**Authors:** Klaudia Giercuszkiewicz-Haśnik, Magdalena Skonieczna, Beata Morak-Młodawska, Małgorzata Jeleń

**Affiliations:** 1 Department of Systems Biology and Engineering, Silesian University of Technology, Akademicka Street 16, 44-100 Gliwice, Poland; magdalena.skonieczna@polsl.pl; 2 Centre of Biotechnology, Silesian University of Technology, Krzywoustego Street 8, 44-100 Gliwice, Poland; 3 Faculty of Medical Sciences in Katowice, Medical University of Silesia, 40-752 Katowice, Poland; 4 Department of Organic Chemistry, Faculty of Pharmaceutical Sciences in Sosnowiec, Medical University of Silesia in Katowice, Jagiellońska Street 4, 41-200 Sosnowiec, Poland; bmlodawska@sum.edu.pl

**Keywords:** phenothiazine derivatives, zidovudine (AZT), colorectal cancer, apoptosis, cell cycle arrest, anticancer activity

## Abstract

Colorectal cancer is the third most commonly diagnosed cancer worldwide and the second leading cause of cancer-related deaths, while its resistance to treatment continues to represent a major therapeutic challenge. In the present study, a series of phenothiazine derivatives, including hybrids containing a 1,2,3-triazole linker and the zidovudine (AZT) fragment, were synthesized and evaluated for their anticancer activity against colorectal cancer cell lines HCT116 and HT-29 as well as non-cancerous BEAS-2B cells. Cytotoxic activity was determined using the Alamar Blue assay, while the mechanisms of action were investigated by flow cytometric analysis of apoptosis, cell cycle progression, and reactive oxygen species (ROS) generation. Additionally, changes in the expression of genes associated with apoptosis, oxidative stress, and DNA damage response were analyzed by RT-qPCR. The obtained results demonstrated that AZT-containing derivatives exhibited stronger anticancer activity than non-conjugated phenothiazine analogs. Compounds **A9–A12** induced pronounced apoptosis and significant disturbances in cell cycle progression, particularly in HCT116 cells. Among the analyzed derivatives, compound **A9** displayed the most favorable overall biological profile, combining strong proapoptotic and cytotoxic activity with relatively high selectivity toward cancer cells and moderate effects on non-cancerous cells. The results indicate that molecular hybridization of phenothiazine derivatives with the AZT scaffold represents a promising strategy for the development of novel anticancer agents targeting colorectal cancer.

## 1. Introduction

Despite advances in modern oncology, cancer continues to represent a major therapeutic and clinical challenge worldwide. Among solid tumors, colorectal cancer remains one of the most frequently diagnosed malignancies and one of the leading causes of cancer-related mortality, with treatment resistance still significantly limiting the effectiveness of available therapies [1]. The rational design of hybrid small-molecule therapeutics—wherein two or more pharmacophores are covalently linked to create multifunctional agents—has emerged as a powerful strategy in drug discovery [2]. Molecular hybridization can produce compounds with improved biological efficacy, enhanced selectivity, and polypharmacological profiles by simultaneously engaging multiple biological targets [3,4,5]. Among the favored heterocyclic scaffolds, phenothiazine and its structural analogues have been extensively studied for their broad spectrum of biological activity [6,7,8,9]. Phenothiazines were first synthesized in the late 19th century, and since then, research has been conducted on the synthesis of new derivatives [10,11,12]. New phenothiazine analogues containing modified substituents on the thiazine nitrogen atom, as well as modified phenothiazine systems, have demonstrated among other biological activities antiviral, antibacterial, anticancer, anti-inflammatory and antiprotozoal properties [7,13,14,15,16,17,18,19,20,21,22]. Another important area of research is their impact on the central nervous system, where neuroprotective effects and potential use in the treatment of neurodegenerative diseases such as Alzheimer’s and Parkinson’s disease are observed. These mechanisms are often associated with antioxidant properties and the ability to stabilize cell membranes. Furthermore, structural modifications of the phenothiazine ring allow for the creation of compounds with selective biological activity and reduced toxicity, which is crucial for further pharmacological development [23,24,25]. The 1,2,3-triazole ring is also a widely used pharmacophoric element in medicinal chemistry. Huisgen’s 1,3-dipolar cycloaddition (“click chemistry”) enables the efficient formation of 1,4-disubstituted triazoles with high regiochemical control, yielding molecules with favorable metabolic stability and hydrogen-bonding capacity [26,27,28]. The triazole moiety is present in numerous biologically active compounds with antimicrobial, antiviral, anticancer, antituberculosis, and anti-inflammatory properties. Hybridization of the triazole ring with other heterocycles often leads to compounds with improved biological profiles compared to their parent scaffolds [29,30,31,32,33,34]. A further dimension of molecular hybridization is the incorporation of nucleoside fragments such as 3′-azido-3′-deoxythymidine (AZT), the archetypal antiviral nucleoside reverse transcriptase inhibitor. 3′-Azido-3′-deoxythymidine (AZT, also known as zidovudine) is a synthetic nucleoside analogue that played a pioneering role in the treatment of human immunodeficiency virus type 1 (HIV-1) infection. Originally synthesized in the 1960s as a potential anticancer agent, it quickly proved ineffective for this indication, but it was distinguished by its ability to inhibit retroviral reverse transcriptase activity by replacing the 3′-hydroxyl group with an azido group, leading to the termination of DNA chain elongation. Because of this property, AZT became the first drug approved by the FDA for the treatment of HIV infection in 1987, representing a breakthrough in the clinical treatment of AIDS and the prevention of vertical transmission of the virus from mother to child [35,36,37]. The activity of AZT is based on its phosphorylation to the active triphosphate form (AZT-TP), which becomes incorporated into viral DNA, leading to termination of chain elongation [38,39]. However, despite its relatively low affinity for mammalian DNA polymerases, AZT-TP has also been shown to be incorporated into eukaryotic DNA, including telomeric regions of the genome, which initiated further investigations into the anticancer potential of this compound [39,40,41]. Interest in the oncological applications of AZT increased substantially following the discovery of functional similarities between telomerase and HIV-1 reverse transcriptase [38,39]. Telomerase remains inactive in most normal somatic cells, whereas its activity has been detected in 85–90% of human cancers, making it an attractive therapeutic target [39]. AZT has been shown to reduce telomerase activity and inhibit the proliferation of cancer cells in several malignancies, including esophageal, colorectal, breast, ovarian, parathyroid, and hepatocellular cancers [38,39,42,43,44]. In HT-29 colorectal cancer cells, exposure to 125 μM AZT resulted in telomerase inhibition, reduced population doubling, and progressive telomere shortening over a period of 91 days [45]. In contrast, significant inhibition of telomerase activity in parathyroid cancer cells was observed after 48 h incubation with 100 μM AZT and was associated with suppressed cellular proliferation [46]. It has also been reported that AZT-induced telomere shortening may be irreversible, as shortened telomeres did not undergo re-elongation even after removal of the compound from the culture medium [39]. At the same time, activation of the alternative lengthening of telomeres (ALT) pathway was proposed as a potential compensatory mechanism limiting the effectiveness of anti-telomerase therapy [39]. Numerous studies demonstrated that the anticancer activity of AZT is closely associated with disruption of cell cycle progression [38,40,42,43,44]. In TE-11 esophageal cancer cells, AZT treatment caused a dose-dependent decrease in telomerase activity and the proportion of cells in the G1/G0 phase, accompanied by an increased percentage of cells in the S phase [38]. Similar observations were reported in HepG2 cells, where AZT decreased the proportion of cells in G1/G0 while simultaneously increasing the percentage of cells in the S and G2/M phases; this effect was particularly evident during the early stage of treatment with 100 μM AZT [40]. Likewise, studies on ovarian cancer cells demonstrated that cell cycle arrest constituted one of the principal mechanisms underlying the anticancer effects of AZT, although the observed responses depended on the cell type [44]. In CaOV3 cells, AZT induced S-phase arrest associated with upregulation of *p16* expression, whereas TOV21G cells exhibited G2/M arrest accompanied by increased *p21* expression [44]. Furthermore, regulation of *p21* expression was shown to occur through both p53-dependent and p53-independent pathways [44]. The mechanism of AZT action has also been linked to DNA damage induction and activation of the cellular DNA damage response [38,40,42,43]. Increased expression of γ-H2AX as well as phosphorylated Chk1 and Chk2 was observed in AZT-treated cells, indicating activation of DNA damage signaling pathways [38,40,42,43]. Simultaneously, total Chk1 and Chk2 protein levels were reduced, with statistically significant decreases observed at concentrations ≥20 μM AZT, suggesting activation of cell cycle checkpoint pathways [40]. The authors proposed that AZT-induced DNA damage leads to cell cycle arrest, thereby enabling DNA repair or triggering cell death [40]. It was also suggested that at least part of the DNA damage induced by AZT may be reversible, since partial or complete reversal of selected biological effects was observed following removal of the compound [39,40]. In addition, cancer cells were found to be more sensitive to AZT than normal cells, which was attributed to their higher proliferation rate and increased thymidine turnover [40]. Another important aspect of the anticancer activity of AZT involves activation of p53-related signaling pathways [43,47,48,49,50]. In colorectal cancer cells, AZT activated the p53-Puma/Bax/Noxa pathways promoting apoptosis, as well as the p53-p21 axis responsible for cell cycle arrest [47]. Aneuploid cells were more sensitive to AZT-induced cell cycle arrest and DNA double-strand break formation, whereas euploid cells displayed greater susceptibility to AZT-induced apoptosis mediated by the p53-Puma/Bax/Noxa pathway [47]. Long-term exposure to AZT also resulted in increased expression of *p14ARF* and *TP53* together with elevated *MDM2* expression and reduced *MDMX* levels in HTLV-I-associated T-cell leukemia cells. The authors suggested that p14ARF stabilizes p53 by preventing *MDM2*-mediated p53 degradation while simultaneously promoting *MDM2*-dependent degradation of *MDMX*, thereby eliminating an additional inhibitor of p53 function [50]. Induction of apoptosis also represents an important consequence of AZT treatment [38,40,43,44,51]. Reduced telomerase activity was shown to promote telomere destabilization, impaired DNA repair, and activation of apoptotic pathways [38]. Increased apoptotic cell death following AZT exposure was observed in cancer cells, particularly in those characterized by active telomerase [39,40]. In EBV-positive lymphomas with low *BCL2* expression and elevated CD95 levels, high sensitivity to AZT-induced apoptosis was observed, whereas lymphomas expressing high levels of *BCL2* remained resistant to treatment [51]. Consequently, it was proposed that patients with tumors characterized by high *BCL2* expression may be resistant to AZT, while tumors with lower *BCL2* levels could potentially benefit from AZT-based combination therapies [51]. Several studies also emphasized the contribution of oxidative stress and mitochondrial dysfunction to the mechanism of AZT activity [42,52,53,54,55]. Zidovudine has been shown to increase reactive oxygen species (ROS) production, enhance the release of pro-inflammatory cytokines, and disrupt mitochondrial bioenergetic processes [53,55]. AZT-induced mitochondrial toxicity includes oxidative stress, disturbances in L-carnitine metabolism, and direct impairment of mitochondrial function [53]. In head and neck cancer cells, AZT enhanced cisplatin cytotoxicity through induction of oxidative stress and disruption of thiol metabolism [42,52]. Altered expression of *OGG1*, an enzyme involved in the repair of oxidative DNA damage, was also observed [54]. Despite numerous reports supporting the anticancer potential of AZT, its application as a single-agent anticancer drug proved to be limited and was eventually abandoned [39,52,56,57]. One of the major limitations was the relatively slow mechanism of action resulting from the need for progressive telomere shortening to reach a critical length [39,41,56,58]. At the same time, the dynamic nature of cancer progression was emphasized, as tumor cells may continue to proliferate for multiple divisions before a therapeutic effect becomes evident, potentially threatening patient survival [39]. It was also suggested that telomere dysfunction may contribute to oncogene activation or silencing of tumor suppressor genes [41,59]. Furthermore, telomerase inhibitors may also affect normal highly proliferative cells, including stem cells and germ cells, in which telomerase activity is maintained in contrast to most somatic tissues [39,41]. Some authors additionally highlighted the potential carcinogenic properties of AZT together with the risk of significant adverse effects limiting its clinical applicability [41]. Systemic toxicity constituted another important limitation of AZT-based therapy [53,57]. Reported adverse effects included mitochondrial toxicity, enhanced oxidative stress, impaired mitochondrial bioenergetics, severe hematological toxicity, and myopathy [50,57]. In a phase II clinical trial evaluating methotrexate in combination with AZT in patients with advanced pancreatic cancer and hepatocellular carcinoma, the treatment was considered ineffective, while severe anemia was observed in half of the patients [57]. Despite the limited efficacy of AZT monotherapy, numerous studies demonstrated synergistic effects of AZT with other anticancer modalities [38,39,40,41,42,60]. AZT has been shown to potentiate the effects of 5-fluorouracil, cisplatin, paclitaxel, and radiotherapy [39,41,42,44,60]. In HT-29 colorectal cancer cells, the presence of AZT increased the cytotoxicity of 5-fluorouracil, while experimental studies additionally demonstrated enhanced DNA strand break formation during combination treatment [45,60]. Experimental data further indicated that AZT-induced DNA strand breaks are intensified by 5-fluorouracil in metastatic colorectal cancer [60]. Consequently, AZT has increasingly been considered primarily as a component of combination therapies and polypharmacology-based strategies, as well as a structural platform for the design and synthesis of novel AZT-derived hybrid compounds [41,52].

The AZT core has served as a versatile synthon for preparing antiviral and anticancer analogues through exploitation of its azido functionality via click chemistry. Incorporation of triazole-linked substituents can modulate antiviral activity and cellular uptake, as demonstrated in numerous AZT-derived triazoles with submicromolar inhibition of HIV-1 reverse transcriptase and other viral targets. While many such derivatives bear simple aryl or heterocyclic groups, significantly less attention has been devoted to integrating AZT into complex heterocyclic hybrid frameworks [61,62,63,64,65]. Our earlier studies showed that purposeful modification of the phenothiazine core by introducing one or two quinoline moieties—resulting in the generation of quinobenzothiazine and diquinothiazine systems—represents a viable approach to broadening the pharmacological spectrum of this scaffold. Several quinoline-containing phenothiazine derivatives demonstrated marked antiproliferative activity across a range of human cancer cell lines, while simultaneously displaying antioxidant and significant immunomodulatory properties. In functional assays, these compounds suppressed mitogen-stimulated proliferation of peripheral blood mononuclear cells, diminished TNFα secretion, and influenced the catalytic activity of enzymes including butyrylcholinesterase [25,66,67,68,69,70]. Animal model experiments also substantiated the anti-inflammatory and immunosuppressive potential of these derivatives. The compounds were shown to attenuate delayed-type hypersensitivity responses, alleviate symptoms in models of psoriasis and colitis, and influence the expression of IFNβ together with downstream components of the IFNβ-regulated signaling cascade [71,72]. Overall, these data support the view that quinoline-substituted phenothiazines constitute a valuable and pharmacologically versatile group of compounds with prospective utility in oncological and autoimmune indications. This therapeutic potential warrants further chemical refinement of the scaffold and comprehensive structure–activity relationship (SAR) investigations aimed at optimizing their biological profile.

**Figure 1:**
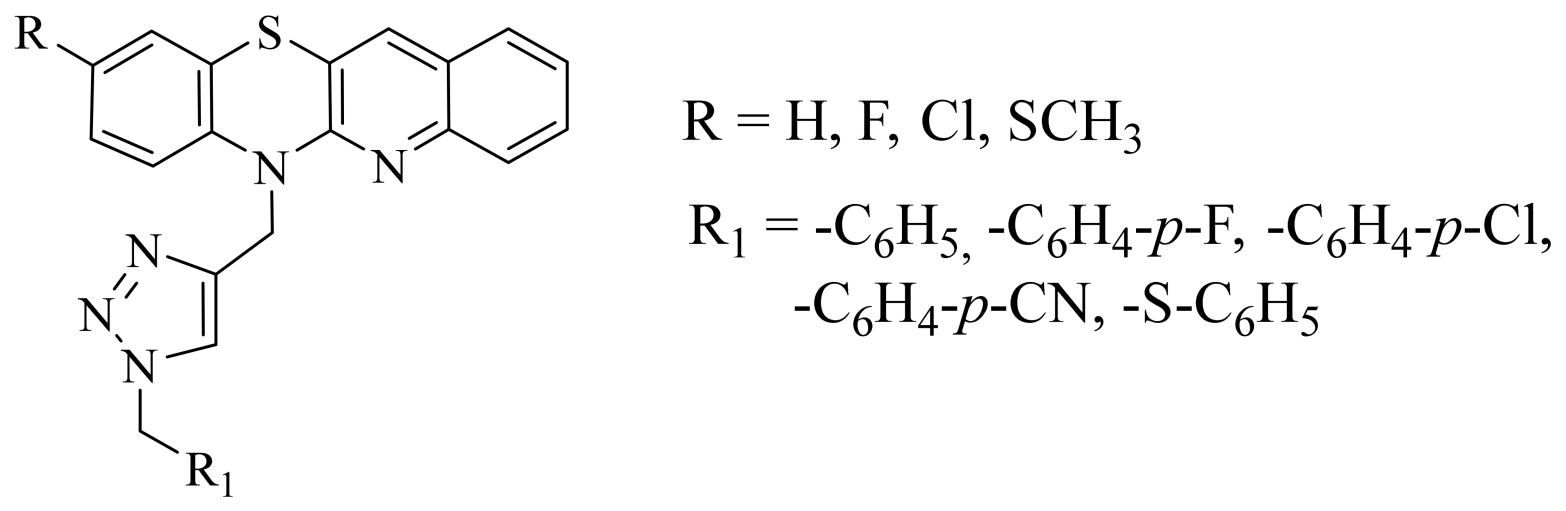
1,2,3-Triazole-quinobenzothiazine hybrids.

Our previous article demonstrated that 1,2,3-triazole-quinobenzothiazine hybrids (Figure 1) constitute an interesting class of compounds with immunomodulatory potential, with relatively limited cytotoxicity against the studied tumor lines. The resulting derivatives, differing in the substituents at position 9 of the quinobenzothiazine system and the nature of the 1,2,3-triazole ring moiety, demonstrated the ability to modulate the expression of genes associated with cell survival (*BCL2*, *MDM2*, *AIFM2*) and the inflammatory response (*IL6*, *IL8*), with particularly pronounced activity observed for derivatives containing sulfur-containing substituents. These results indicate that modifying the structure of the classical phenothiazine skeleton by introducing a 1,2,3-triazole ring is an effective strategy for designing compounds with targeted biological activity, consistent with the concept of molecular hybridization [73].

**Figure 2:**
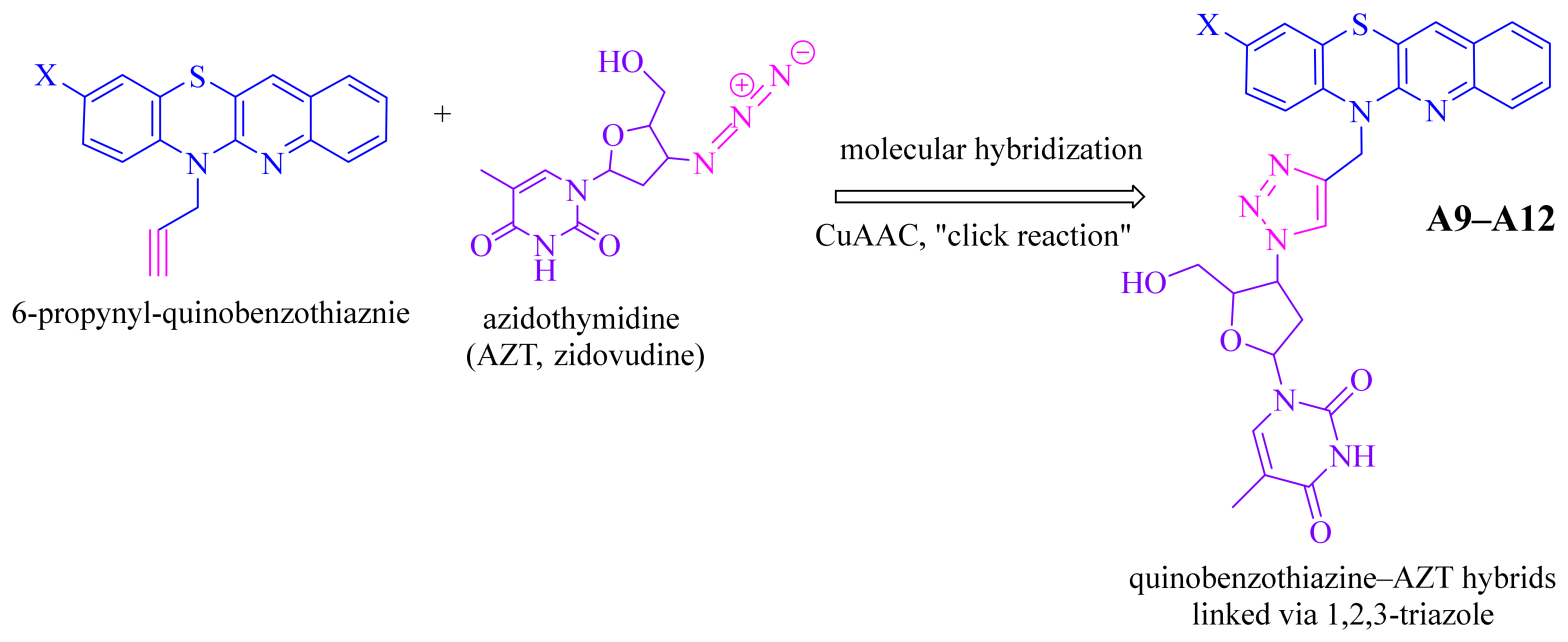
Molecular hybridization strategy used for the design of quinobenzothiazine–triazole–AZT derivatives.

The next step in our research, and the subject of this publication, is the further functionalization of the quinobenzothiazine system by introducing a fragment derived from azidothymidine (AZT) into the 1,2,3-triazole ring (Figure 2). AZT, as a nucleoside analog with a well-characterized biological profile, may impart additional properties to the designed hybrids related to their influence on the replication processes and metabolism of nucleic acids. Incorporating the AZT structure into the quinobenzothiazine-triazole system represents a rational extension of the current design strategy, enabling the creation of multifunctional molecules combining cytostatic properties, anticancer potential, and a nucleoside component. In the present study, 9-substituted quinobenzothiazine skeletons bearing hydrogen, fluorine, chlorine, or the SCH_3_ group were selected as precursor structures. These substituents were chosen to evaluate how changes in electronic character, lipophilicity, solubility, and potential membrane interactions affect biological activity. After conjugation with the AZT fragment through the 1,2,3-triazole linker, the same substituent pattern was retained in the final hybrids, enabling direct comparison of its influence on the physicochemical and anticancer properties of the resulting compounds.

## 2. Results and Discussion

### 2.1. Synthesis of AZT-1,2,3-Triazolo-Quinobenzothiazine Hybrids

**Scheme 1:**
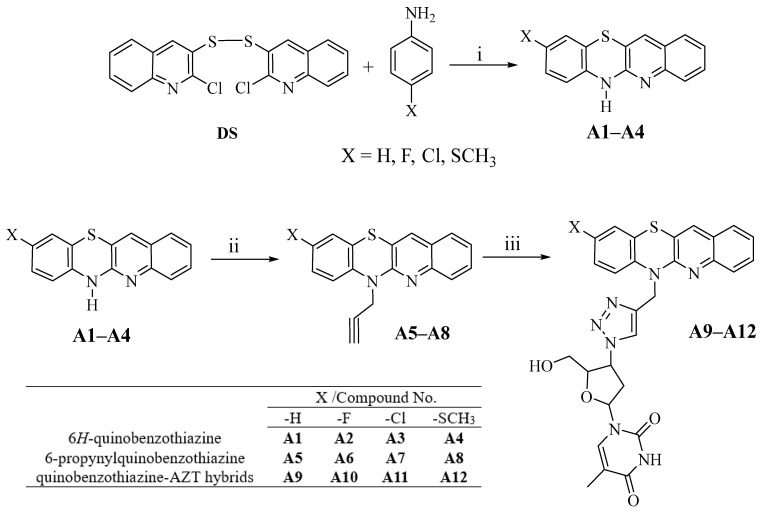
Synthesis of AZT-1,2,3-triazolo-quinobenzothiazine hybrids **A9–A12**. Reagents and reaction conditions: (i) MEDG (monomethyl ether of diethylene glycol), reflux, 3 h [75]; (ii) DMF (dimethylformamide), t-BuOK, ambient temperature, 1 h, 3-bromo-1-propyne, 24 h [70]; (iii) DMF/H_2_O, CuSO_4_ × 5H_2_O, sodium ascorbate, AZT (azidothymidine), ambient temperature, 48 h.

Continuing our research on the design of biologically active quinobenzothiazines, based on the molecular hybridization strategy, which involves the rational combination of structural fragments derived from two (or more) known drugs, we designed and synthesized hybrids consisting of a 9-substituted quinobenzothiazine skeleton linked via a 1,2,3-triazole linker to an AZT fragment. The goal of our work was to obtain new chemical compounds that combine the favorable pharmacodynamic properties of the three starting structures. As demonstrated in the introduction, both phenothiazines, 1,2,3-triazoles, and AZT are valuable chemical motifs in drug design due to their broad spectrum of biological activity. Therefore, the aim of our study was to synthesize and evaluate hybrids of quinobenzothiazines with AZT and 1,2,3-triazole for immunomodulatory and cytotoxic activity against selected cancer cell lines. Hybridization of phenothiazines with 1,2,3-triazoles substituted with an AZT fragment at the nitrogen atom of the triazole ring is based on the Huisgen cycloaddition reaction, known as “click chemistry.” This reaction allows efficient and selective formation of the triazole bond between the corresponding azide and alkyne groups [74]. The synthetic strategy was based on a previously developed route for functionalization of the nitrogen atom at position 6 of the quinobenzothiazine core by introducing a propargyl substituent, which enabled the use of these compounds in a cycloaddition reaction. The corresponding N*H*-quinobenzothiazines (6*H*-quino[3,2-b]benzo[1,4]thiazine **A1**, 6*H*-9-fluoroquino[3,2-b]benzo[1,4]thiazine **A2**, 6*H*-9-chloroquino[3,2-b]benzo[1,4]thiazine **A3**, and 6*H*-9-methylthioquino[3,2-b]benzo[1,4]thiazine **A4**) were synthesized according to the procedures developed and described in the literature [75]. Quinobenzothiazines **A1–A4** were then used as substrates in the reaction with 3-bromo-1-propyne (t-BuOK/DMF) to obtain 6-propynyl derivatives (**A5–A8**). This step was also performed according to the previously described methodology [70]. The title quinobenzothiazine-AZT hybrids linked via a 1,2,3-triazole ring **A9–A12** were obtained from propargylquinobenzothiazines **5–8** using a synthesis based on copper-catalysed azide-alkyne cycloaddition (CuAAC) (Scheme 1). The catalyst required to ensure selective reaction progress at low temperature (Cu^2+^ ions) is generated in situ in the reaction medium by the reduction of Cu^2+^ ions with sodium ascorbate. DMF was used as the solvent in this reaction; the products were isolated from the reaction mixture by pouring the reaction solution into water, followed by filtration of the precipitated solid. The ^1^H NMR, ^13^C NMR and HRMS spectra of the compounds obtained are presented in the Supplementary File.

### 2.2. Alamar Blue Test

Two molecularly distinct colorectal cancer cell lines, HCT116 and HT-29, were selected for this study to evaluate the activity of the investigated compounds in models differing in p53 status, mutational profile, and overall molecular characteristics. In addition, the BEAS-2B cell line was used as a non-cancerous model to assess the selectivity of the analyzed derivatives.

The HCT116 cell line represents a colorectal cancer model harboring an activating KRAS p.Gly13Asp (G13D) mutation while retaining functional wild-type p53 protein [76]. Owing to this molecular background, HCT116 cells are particularly suitable for studies investigating mechanisms related to DNA damage response, oxidative stress, and apoptosis induction. For this reason, this cell line was selected as the primary model for further mechanistic investigations.

The HT-29 cell line represents a colorectal cancer model carrying the BRAF Val600E mutation, which leads to constitutive activation of the MAPK/ERK signaling pathway and consequently promotes cancer cell proliferation and survival Moreover, HT-29 cells harbor a *TP53* mutation resulting in the loss of normal p53 tumor suppressor function through impaired DNA binding and reduced activation of genes involved in cell cycle arrest and apoptosis [77]. These molecular alterations are associated with enhanced tumor cell survival, disease progression, and chemoresistance. Therefore, HT-29 cells are considered a more resistant colorectal cancer model compared with HCT116 cells. In the present study, this cell line was used as an additional screening model to determine whether the investigated compounds retain cytotoxic activity against cells with a more resistant molecular phenotype.

The different molecular background of HCT116 and HT-29 cells may partly explain the differences observed in their sensitivity to the investigated compounds [78,79]. The presence of functional p53 in HCT116 cells may facilitate the activation of DNA damage response pathways, including cell cycle arrest, DNA repair, senescence, and apoptosis [80]. This may contribute to the generally stronger response of HCT116 cells to the analyzed derivatives, particularly in assays related to apoptosis and cell cycle disturbance. In contrast, the mutant p53 status of HT-29 cells may impair canonical p53-dependent apoptotic and cell cycle checkpoint responses, which is consistent with their more resistant phenotype [81].

The BEAS-2B cell line was used as a non-cancerous model to compare the sensitivity of normal and cancer cells to the investigated derivatives. Such an approach allows for a preliminary assessment of the anticancer selectivity of the analyzed compounds and helps determine whether they exhibit stronger activity toward cancer cells than toward normal cells. Although BEAS-2B cells are commonly used as a non-tumorigenic reference model, they are immortalized with an adenovirus 12-SV40 hybrid virus and express SV40 large T antigen; therefore, they should be regarded as an immortalized non-cancerous epithelial model rather than a fully physiological representation of normal tissue [82].

IC_50_ values for the analyzed compounds were calculated on the basis of the obtained cell viability data and are summarized in Table 1. The IC_50_ parameter corresponds to the concentration required to decrease cell viability to 50% compared with untreated control cells and is commonly used as an indicator of cytotoxic potency. Compounds characterized by lower IC_50_ values were considered more biologically active toward the tested cell lines. The results are presented as mean ± standard deviation (SD) calculated from three independent replicate measurements. In cases where the 50% viability threshold was not reached within the tested concentration range, approximate IC_50_ values were estimated by linear extrapolation and treated only as indicative values. However, when extrapolated IC_50_ values markedly exceeded the analyzed concentration range or could not be reliably estimated, the results were reported as IC_50_ > 100 µM. Representative cell viability curves illustrating the relationship between compound concentration and cell viability for the analyzed derivatives are provided in the Supplementary Materials as Figure S1.

Based on the obtained results, compounds **A9**, **A11**, and **A12** appeared to be the most promising derivatives due to their relatively low IC_50_ values toward both colorectal cancer cell lines, particularly after 72 h incubation. Among them, compound **A12** demonstrated the strongest cytotoxic activity against HCT116 cells after 24 h treatment (37.0 ± 1.3 µM). In turn, **A9** maintained comparably strong activity against both HCT116 and HT-29 cells while simultaneously exhibiting higher IC_50_ values toward BEAS-2B cells, suggesting a favorable selectivity profile. Compound **A11** also showed pronounced anticancer activity after prolonged incubation, especially against HCT116 (46.7 ± 0.2 µM) and HT-29 cells (56.0 ± 1.4 µM); however, a marked decrease in BEAS-2B cell viability was also observed after 72 h exposure.

Compound **A10** also demonstrated notable cytotoxic activity toward both colorectal cancer cell lines, particularly after 72 h incubation, with IC_50_ values of 72.0 ± 1.0 µM for HCT116 and 80.5 ± 3.3 µM for HT-29 cells. At the same time, after 24 h treatment, this derivative exhibited lower toxicity toward BEAS-2B cells (113.7 ± 9.1 µM), which may indicate a favorable selectivity profile during shorter exposure periods. Nevertheless, prolonged incubation resulted in substantially lower IC_50_ values in non-cancerous cells, indicating increased toxicity after 72 h treatment.

Compound **A8** displayed moderate anticancer activity accompanied by relatively higher IC_50_ values toward BEAS-2B cells after 24 h incubation, which may suggest partial selectivity toward cancer cells. In contrast, compound **A5** showed time-dependent cytotoxicity and reduced selectivity following prolonged exposure. Although moderate activity against colorectal cancer cells was observed, the IC_50_ value determined for BEAS-2B cells after 72 h was also relatively low (49.3 ± 0.5 µM), indicating increased toxicity toward non-cancerous cells.

Conversely, compounds **A6** and **A7** demonstrated limited biological activity, with IC_50_ values exceeding 100 µM in most analyzed conditions, particularly after 72 h incubation. Similarly, compounds **A1–A4** were characterized by generally weak cytotoxic effects toward the tested cell lines.

Notably, derivatives containing the AZT moiety (**A9–A12**) generally exhibited higher anticancer activity than the earlier compounds from the series, which may suggest that incorporation of the AZT fragment positively influenced the biological properties of the analyzed derivatives.

### 2.3. Selectivity Index Analysis

To further evaluate the biological profile of the most active derivatives, the selectivity index (SI) was determined for compounds **A9–A12** containing the AZT moiety. The SI parameter was calculated to assess the preferential cytotoxic activity of the analyzed compounds toward colorectal cancer cells compared with non-cancerous BEAS-2B cells. SI values were determined according to the following equation:

SI = IC_50_ (BEAS-2B)/IC_50_ (cancer cells)

An SI value greater than 1 indicated preferential activity toward cancer cells, whereas values below 1 suggested increased toxicity toward non-cancerous cells (Table 2).

Among the analyzed AZT-containing derivatives, compounds **A9** and **A12** demonstrated the most favorable selectivity profiles, particularly after 24 h incubation. The highest selectivity was observed for compound **A12** against HCT116 cells (SI = 2.82), indicating markedly stronger cytotoxic activity toward colorectal cancer cells than toward BEAS-2B cells. Compound **A9** also exhibited consistently beneficial selectivity values toward both HCT116 and HT-29 cells at both analyzed time points, maintaining SI values above 1 even after prolonged incubation.

Compound **A10** displayed moderate selectivity after 24 h treatment; however, longer incubation resulted in a decrease in SI values associated with increased cytotoxicity toward non-cancerous cells. A similar effect was observed for compound **A11**, which despite strong anticancer activity showed reduced selectivity after 72 h exposure due to elevated toxicity toward BEAS-2B cells. Overall, the obtained data suggest that AZT-containing derivatives, particularly **A9** and **A12**, combine relatively high anticancer activity with a favorable selectivity profile toward colorectal cancer cells.

Interestingly, compound **A2** also demonstrated a favorable biological profile despite the absence of fully calculable SI values at certain time points due to IC_50_ values exceeding 100 µM in BEAS-2B cells. This observation may suggest relatively low toxicity toward non-cancerous cells while maintaining detectable anticancer activity against HCT116 cells. These findings suggest that the modified phenothiazine scaffold itself contributes to anticancer activity independently of the AZT fragment.

### 2.4. Microscopic Evaluation of Morphological Changes

**Figure 3:**
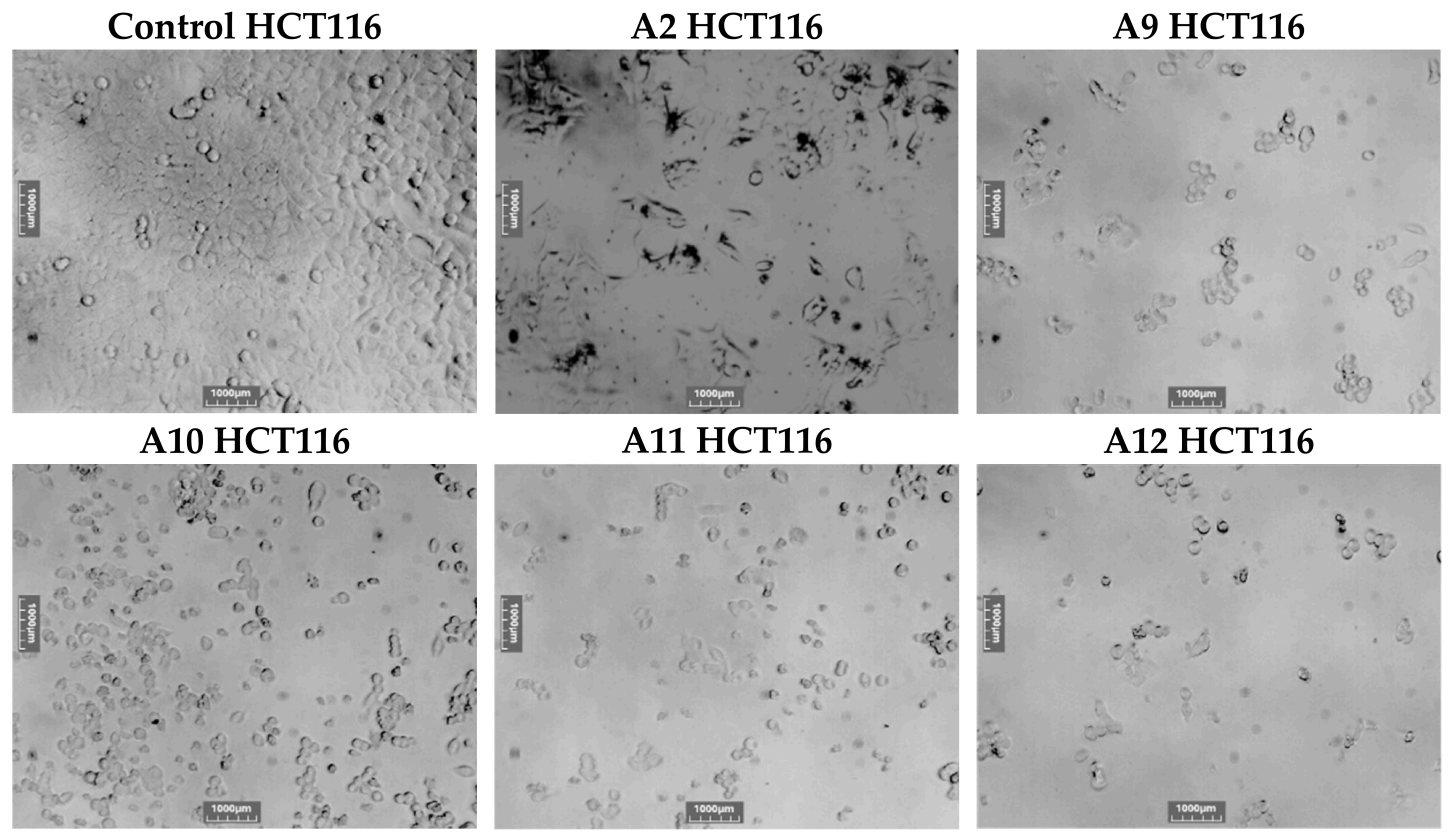

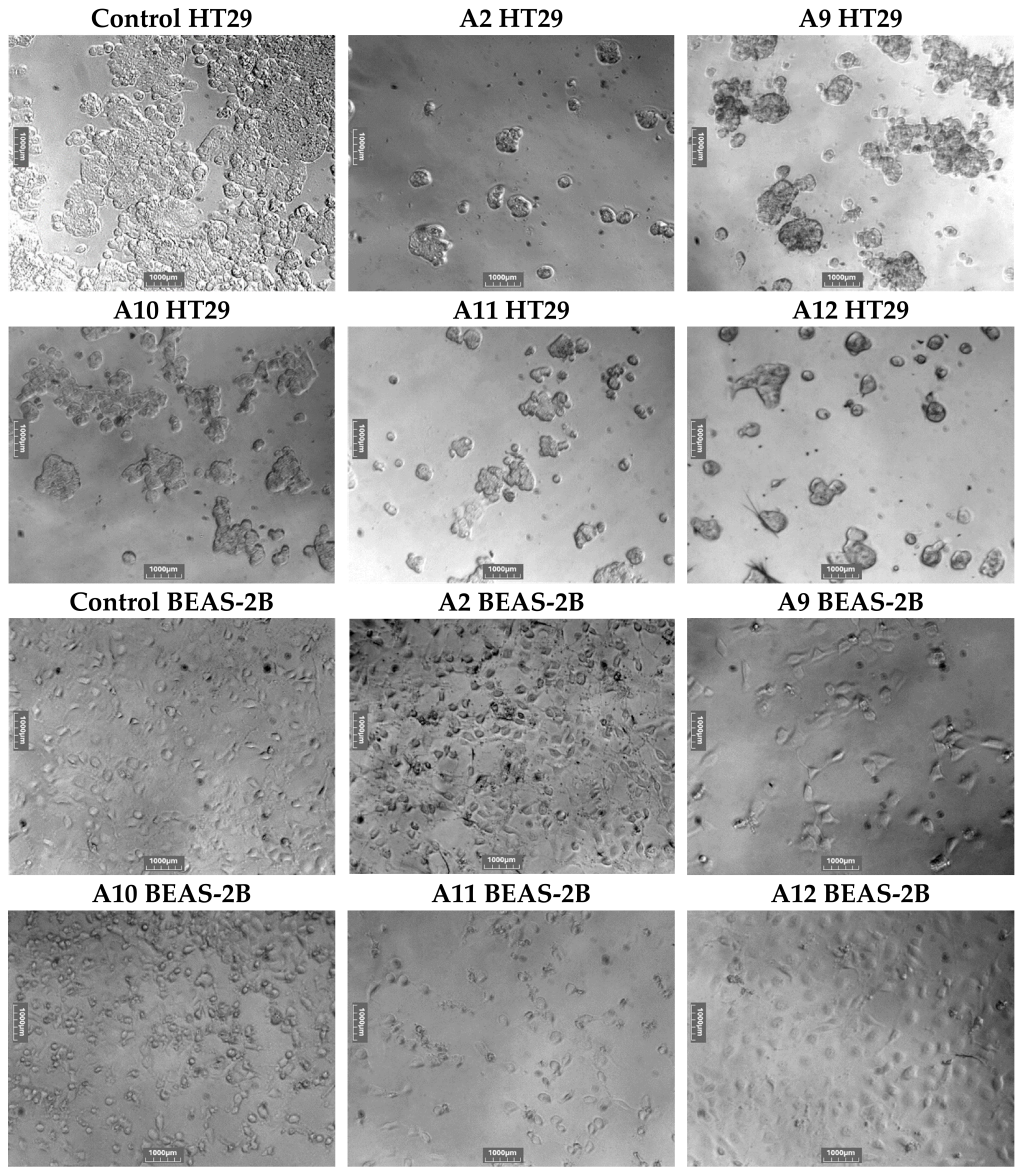
Representative microscopic images of HCT116, HT-29, and BEAS-2B cells after 24 h incubation with compounds **A2** and **A9–A12** (100 µM). Control cells were cultured without treatment. Magnification: 10×.

Microscopic evaluation was carried out using HCT116, HT-29, and BEAS-2B cell lines following 24 h exposure to compounds **A1–A12** at a concentration of 100 µM under standard culture conditions (37 °C, 5% CO_2_, humidified atmosphere). HCT116 cells, characterized by wild-type p53 status, and HT-29 cells harboring a *TP53* mutation were included as molecularly distinct colorectal cancer models, while BEAS-2B cells served as a non-cancerous reference line. Representative microscopic images of HCT116, HT-29, and BEAS-2B cells treated with compounds **A2** and **A9–A12** are presented in Figure 3, as these derivatives demonstrated the most relevant biological activity in the performed experiments. Microscopic images of all investigated cell lines following treatment with compounds **A1–A12** are provided in the Supplementary Materials (Figures S2–S4). Figure S2 presents representative microscopic images of HCT116 cells, Figure S3 of HT-29 cells, and Figure S4 of BEAS-2B cells after 24 h treatment with compounds **A1–A12** at a concentration of 100 µM under standard culture conditions. Images were acquired at 10× magnification.

Microscopic observations revealed significant differences in the morphology and confluence of colorectal cancer and non-cancerous cells following treatment with compounds **A1–A12**. Untreated control cultures of HCT116, HT-29, and BEAS-2B cells exhibited typical morphology and high confluence. Among the analyzed derivatives, the most pronounced morphological alterations in HCT116 cells were observed after treatment with compounds **A9–A12**. These compounds caused a marked reduction in cell confluence accompanied by an increased number of rounded and detached cells, indicating cytotoxic activity.

Similar effects were observed in HT-29 cells; however, the extent of morphological disturbances was slightly less pronounced compared with HCT116 cells, which may reflect the higher resistance of the HT-29 model. In BEAS-2B cells, morphological changes were generally less evident than in cancer cells, although treatment with compounds **A9** and **A11** also resulted in a noticeable decrease in cell confluence.

### 2.5. RT-qPCR

Real-time quantitative polymerase chain reaction (RT-qPCR) analysis was performed for the reference gene *RPL41* (ribosomal protein L41) and the target genes *BCL2*, *AIFM2*, *MDM2*, *OGG1*, *IL6*, and *IL8*. The selected target genes were analyzed to evaluate the potential effects of the investigated compounds on cellular pathways associated with apoptosis regulation, oxidative stress response, inflammatory signaling, DNA damage response, and cell survival mechanisms. *RPL41* was selected as the reference gene due to its stable expression profile and the absence of significant expression variability between the analyzed cell lines and experimental conditions.

The experiments were conducted using HCT116 and BEAS-2B cells as representative models of colorectal cancer and non-cancerous cells, respectively. The HCT116 cell line was additionally selected due to its preserved wild-type p53 status, enabling the evaluation of cellular responses associated with p53-dependent pathways, including cell cycle regulation, DNA damage response, and apoptosis induction. Compounds demonstrating the most promising biological activity in the previously performed experiments were selected for further molecular evaluation.

**Figure 4:**
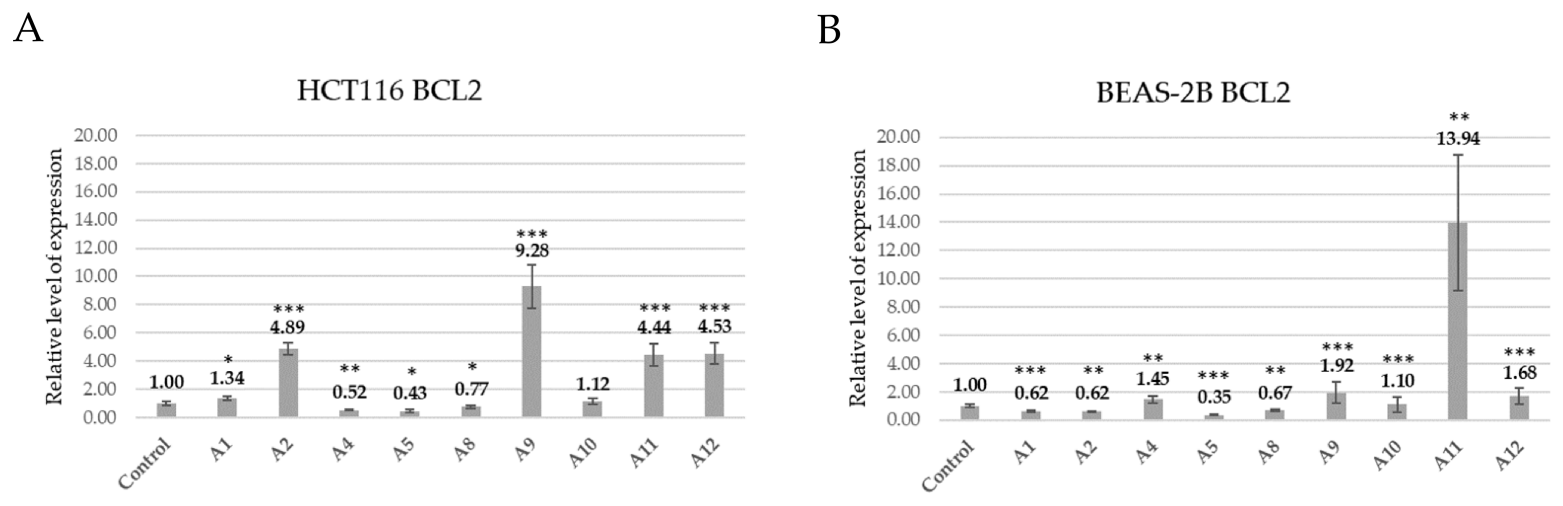

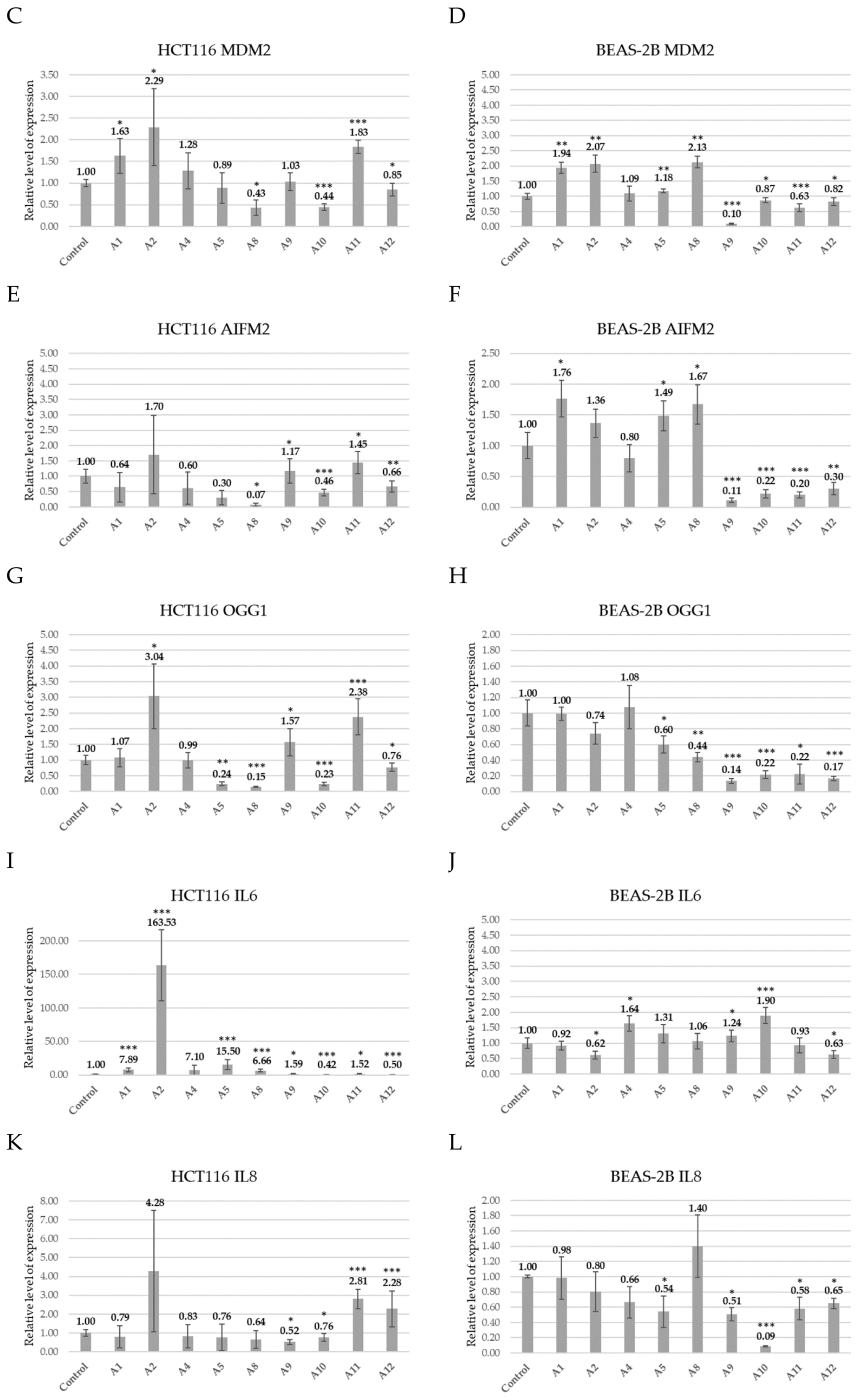
Relative expression levels of *BCL2*, *AIFM2*, *MDM2*, *OGG1*, *IL6*, and *IL8* genes in HCT116 and BEAS-2B cells after 24 h incubation with the selected compounds at a concentration of 100 μM, determined by RT-qPCR analysis. Panels: (**A**) *BCL2* expression in HCT116 cells; (**B**) *BCL2* expression in BEAS-2B cells; (**C**) *MDM2* expression in HCT116 cells; (**D**) *MDM2* expression in BEAS-2B cells; (**E**) *AIFM2* expression in HCT116 cells; (**F**) *AIFM2* expression in BEAS-2B cells; (**G**) *OGG1* expression in HCT116 cells; (**H**) *OGG1* expression in BEAS-2B cells; (**I**) *IL6* expression in HCT116 cells; (**J**) *IL6* expression in BEAS-2B cells; (**K**) *IL8* expression in HCT116 cells; (**L**) *IL8* expression in BEAS-2B cells. Relative gene expression levels were calculated using the 2^−ΔΔCt^ method and normalized to the reference gene *RPL41*. The results are presented as mean ± SD from three independent experiments (*n* = 3). Statistical significance was determined using an unpaired two-tailed Welch’s *t*-test based on ΔCt values. * *p* < 0.05, ** *p* < 0.01, *** *p* < 0.001 compared to the control group.

Gene expression levels were assessed after 24 h incubation of the cells with the tested compounds at a concentration of 100 μM. The selection of this concentration was based both on the results of the performed biological studies and on literature reports concerning AZT activity. Numerous studies demonstrated that the biological effects of zidovudine itself, including telomerase inhibition, induction of DNA damage, and cell cycle disturbances, were most evident following the application of relatively high concentrations and prolonged incubation times. Since gene expression analysis in the present study was performed after 24 h incubation, the use of a 100 μM concentration allowed for the observation of more pronounced biological effects while maintaining comparable experimental conditions for all analyzed compounds. The obtained gene expression results are presented in Figure 4.

RT-qPCR is a method that enables quantitative evaluation of relative gene expression levels based on real-time monitoring of nucleic acid amplification. Relative gene expression levels were calculated using the 2^−ΔΔCt^ method relative to the control group [83]. The results are presented as mean ± standard deviation (SD) from three independent experiments (*n* = 3). Statistical analysis was performed using ΔCt values and an unpaired two-tailed Welch’s *t*-test. Statistical significance was defined as follows: * *p* < 0.05, ** *p* < 0.01, and *** *p* < 0.001, whereas the absence of asterisks indicated no statistically significant differences compared to the control group.

*BCL2* encodes an anti-apoptotic protein responsible for maintaining mitochondrial membrane stability and inhibiting the activation of mitochondria-dependent apoptotic pathways; therefore, reduced *BCL2* expression may indicate the pro-apoptotic activity of the investigated compounds [84]. The expression profile of the *BCL2* gene differed markedly between HCT116 cancer cells and non-cancerous BEAS-2B cells, suggesting a differential effect of the analyzed compounds on cell survival mechanisms and apoptosis regulation. In HCT116 cells, the strongest increase in *BCL2* expression was observed following treatment with compound **A9**, for which the expression level reached 9.28 relative to the control. Substantially elevated expression was also detected for compounds **A2**, **A11**, and **A12**. In contrast, compounds **A4**, **A5**, and **A8** reduced *BCL2* expression below the control level, which may indicate weakened anti-apoptotic mechanisms and increased susceptibility of the cells to apoptosis induction. Compound **A5** appeared particularly noteworthy, as it resulted in one of the lowest *BCL2* expression levels in HCT116 cells. Compounds **A1** and **A10** caused a slight increase in *BCL2* expression, although in the case of **A10** the difference was not statistically significant. In BEAS-2B cells, the strongest decrease in *BCL2* expression was observed following treatment with **A5**, **A1**, **A2**, and **A8**. An exception was compound **A11**, which induced a pronounced increase in *BCL2* expression to a level of 13.94, markedly exceeding both the control and all other tested compounds. This may indicate activation of protective and anti-apoptotic mechanisms in non-cancerous cells following exposure to **A11**. A moderate increase in expression was also observed for **A9** and **A12**. Comparison of both cell lines revealed that compounds containing the AZT moiety (**A9–A12**) more frequently induced increased *BCL2* expression, particularly in HCT116 cells. This may suggest the activation of adaptive mechanisms associated with the cellular response to stress and DNA damage induced by these hybrids. At the same time, these findings indicate that apoptosis induction by the investigated compounds is likely not solely dependent on direct downregulation of *BCL2* expression, but may also involve more complex mechanisms related to the activation of DNA damage pathways, oxidative stress, and disruption of mitochondrial homeostasis. Despite the increased *BCL2* expression observed particularly after **A9** treatment, subsequent apoptosis analyses demonstrated a very high percentage of apoptotic cells, suggesting that the elevated *BCL2* expression represented a compensatory cellular response insufficient to counteract the strong pro-apoptotic signals induced by the tested compounds.

*MDM2* encodes a protein that serves as the principal negative regulator of p53 and is involved in controlling its stability and degradation; therefore, changes in *MDM2* expression levels may reflect the activation of DNA damage response pathways and mechanisms regulating cell survival [85]. In HCT116 cells, the highest *MDM2* expression levels were observed following treatment with compounds **A2** and **A11**, for which expression values reached 2.29 and 1.83 relative to the control, respectively. Increased expression was also detected for **A1** and **A4**. In contrast, compounds **A8**, **A10**, and **A12** significantly reduced *MDM2* expression below the control level. The strongest effect was observed for **A10**, where the expression level decreased to only 0.44. In the case of **A9**, *MDM2* expression remained comparable to the control. In BEAS-2B cells, increased *MDM2* expression was observed primarily after treatment with **A1**, **A2**, and **A8**, with the highest value recorded for **A8** (2.13). In contrast, compounds containing the AZT moiety, particularly **A9** and **A11**, led to a marked reduction in *MDM2* expression. The strongest decrease was observed for **A9**, for which the expression level reached only 0.10 relative to the control. Reduced expression was also noted for **A10**, **A11**, and **A12**. Comparing both cell lines, it can be observed that the responses of HCT116 and BEAS-2B cells to the investigated compounds differed substantially. In cancer cells, some compounds, particularly **A2** and **A11**, induced increased *MDM2* expression, which may indicate activation of cellular stress and DNA damage response mechanisms associated with the p53-MDM2 axis. At the same time, selected compounds containing the AZT moiety caused a reduction in *MDM2* expression, especially in BEAS-2B cells, which may suggest disruption of mechanisms regulating p53 stability. Compound **A9** appears particularly interesting, as despite its strong pro-apoptotic activity it did not induce increased *MDM2* expression in HCT116 cells, while simultaneously markedly reducing its level in BEAS-2B cells. This may indicate that the mechanism of action of this compound is not based solely on classical activation of the p53-*MDM2* axis, but also involves more complex processes related to the DNA damage response, disruption of cellular homeostasis, and induction of cell death.

*AIFM2*, also known as *FSP1*, functions as a ferroptosis suppressor and participates in protecting cells against oxidative stress and lipid peroxidation; therefore, reduced expression of this gene may increase cellular susceptibility to oxidative stress-induced cell death [86]. In HCT116 cells, the highest *AIFM2* expression level was observed following treatment with compound **A2**, for which the expression value reached 1.70 relative to the control. Increased expression was also detected for **A9** and **A11**. In contrast, compounds **A5** and **A8** caused a pronounced reduction in *AIFM2* expression, to levels of 0.30 and 0.07 relative to the control, respectively. Reduced expression was also observed following treatment with **A10** and **A12**. Compound **A10** appeared particularly interesting, as it significantly decreased *AIFM2* expression to a level of 0.46. In BEAS-2B cells, most compounds lacking the AZT moiety induced increased *AIFM2* expression. The highest levels were observed for **A1**, **A5**, and **A8**, for which expression values exceeded the control level. In contrast, all compounds containing the AZT moiety (**A9–A12**) caused a marked reduction in *AIFM2* expression. The strongest effect was observed for **A9**, where the expression level reached only 0.11 relative to the control. Reduced expression was also noted for **A10**, **A11**, and **A12**. Comparing both cell lines, it can be observed that hybrids containing the AZT moiety tended to reduce *AIFM2* expression, particularly in BEAS-2B cells. This may suggest impairment of protective mechanisms associated with the response to oxidative stress and regulation of ferroptosis. In HCT116 cells, this effect was more heterogeneous, as **A9** and **A11** induced increased *AIFM2* expression, which may reflect compensatory activation of defense mechanisms in response to intensified cellular stress induced by these compounds. At the same time, the very strong pro-apoptotic activity observed for **A9–A12** suggests that even elevated *AIFM2* expression in some cancer cells was insufficient to prevent the induction of cell death. These findings may indicate that the mechanism of action of the investigated compounds involves not only the induction of oxidative stress, but also disruption of pathways responsible for protecting cells against oxidative damage and ferroptosis.

*OGG1* encodes 8-oxoguanine DNA glycosylase, an enzyme involved in the base excision repair (BER) pathway responsible for the removal of oxidatively damaged nitrogenous bases, particularly 8-oxoguanine. Reduced *OGG1* expression may lead to the accumulation of DNA damage and increased susceptibility of cells to oxidative stress-induced cell death [87]. In HCT116 cells, the highest *OGG1* expression level was observed following treatment with compound **A2**, for which the expression value reached 3.04 relative to the control. Increased expression was also detected for **A9** and **A11**. In contrast, compounds **A8**, **A5**, **A10**, and **A12** caused a marked reduction in *OGG1* expression below the control level. The strongest effect was observed for **A8**, **A10**, and **A5**, where expression levels reached 0.15, 0.23, and 0.24, respectively. In BEAS-2B cells, most of the analyzed compounds reduced *OGG1* expression. A particularly strong decrease was observed for compounds containing the AZT moiety (**A9–A12**), for which expression levels remained markedly below the control. The lowest value was observed for **A9**, where *OGG1* expression reached only 0.14 relative to the control. Reduced expression was also observed for **A5** and **A8**, whereas the remaining compounds did not induce significant changes compared to the control group. Comparing both cell lines, it can be observed that compounds containing the AZT moiety tended to reduce *OGG1* expression, particularly in BEAS-2B cells. This may suggest impairment of oxidative DNA damage repair mechanisms and increased susceptibility of cells to oxidative stress induced by the investigated hybrids. In HCT116 cells, the response was more heterogeneous, as **A9** and **A11** increased *OGG1* expression, which may reflect activation of compensatory repair mechanisms in response to intensified DNA damage. At the same time, compounds such as **A8** and **A10** caused a pronounced reduction in *OGG1* expression, which may promote the accumulation of oxidative damage and enhance the induction of cell death. These findings suggest that the mechanism of action of the investigated compounds may involve both the induction of oxidative stress and modulation of the cellular capacity to repair oxidative DNA damage.

IL6 and IL8 are pro-inflammatory cytokines that play important roles in the regulation of the tumor microenvironment. These cytokines are involved, among others, in angiogenesis, proliferation, and migration of cancer cells, and may also support tumor progression and metastasis formation, while their elevated levels are associated with poor prognosis [88,89]. In HCT116 cells, the strongest increase in the expression of both *IL6* and *IL8* was observed following treatment with compound **A2**. *IL6* expression reached a value of 163.53 relative to the control, whereas *IL8* expression increased to 4.28. A marked increase in *IL6* expression was also observed following treatment with compounds **A1**, **A5**, and **A8**. A different expression profile was observed for derivatives containing the AZT moiety. Compounds **A10** and **A12** reduced *IL6* expression, whereas **A9** and **A11** induced a moderate increase in *IL8* expression. At the same time, **A9** and **A10** reduced *IL8* expression, while **A11** and **A12** increased its level. The remaining compounds did not significantly affect *IL8* expression in HCT116 cells. In BEAS-2B cells, the cytokine expression profile differed substantially. In the case of *IL6*, the highest level was observed following treatment with **A10**, where expression reached 1.90 relative to the control. A moderate increase in expression was also observed for **A4**, **A5**, and **A9**. At the same time, some compounds, particularly **A2** and **A12**, reduced *IL6* expression below the control level. In the case of *IL8*, most of the investigated compounds reduced its expression relative to the control. The strongest decrease was observed for **A10**, for which the expression level reached only 0.09. An exception was **A8**, which increased *IL8* expression to a level of 1.40; however, this value was not statistically significant. Comparing both cell lines, it can be observed that the pro-inflammatory response was much more pronounced in HCT116 cancer cells than in BEAS-2B cells. Compound **A2** appears particularly interesting, as it induced very strong *IL6* expression and increased *IL8* levels in HCT116 cells, which may indicate an intense stress response and activation of pathways associated with cellular damage. At the same time, compounds containing the AZT moiety, especially **A9–A12**, did not induce an equally strong pro-inflammatory response despite the high pro-apoptotic activity observed in subsequent experiments. This may suggest that the mechanism of action of these hybrids is more strongly associated with direct induction of DNA damage, disruption of cellular homeostasis, and activation of cell death pathways rather than enhancement of the inflammatory response. These findings also indicate that the high cytotoxicity of the investigated compounds does not always directly correlate with the level of pro-inflammatory cytokine induction.

Overall, the obtained results suggest that conjugation of the AZT moiety with the phenothiazine-triazole scaffold significantly affects the biological profile of the investigated compounds, leading to modulation of pathways associated with the DNA damage response, oxidative stress, apoptosis regulation, and cellular protective mechanisms. In contrast to some derivatives lacking the AZT moiety, hybrids **A9–A12** generally did not induce a very strong pro-inflammatory response associated with *IL6* and *IL8*, which may suggest a more targeted mechanism of action related to direct disruption of cellular homeostasis and activation of cell death pathways. At the same time, the diverse effects of these compounds on the expression of *BCL2*, *MDM2*, *AIFM2*, and *OGG1* indicate that their anticancer activity is likely associated with a multidirectional mechanism involving both the induction of cellular stress and modulation of the cellular capacity to repair DNA damage and counteract oxidative stress.

### 2.6. Level of ROS

Reactive oxygen species (ROS) play an important role in the regulation of numerous cellular processes, including proliferation, differentiation, inflammatory response, and cell death [90]. Under physiological conditions, ROS levels are controlled by cellular antioxidant mechanisms; however, excessive production of reactive oxygen species leads to oxidative stress, damage to lipids, proteins, and DNA, as well as impairment of mitochondrial function [91]. Cancer cells are often characterized by elevated basal ROS levels, which makes them particularly susceptible to additional disturbances in redox balance induced by anticancer compounds [92,93].

Many compounds with anticancer activity exert their effects through enhancement of oxidative stress, which may lead to activation of the DNA damage response, cell cycle arrest, and induction of apoptosis [93]. On the other hand, excessive ROS induction in normal cells may be associated with non-selective toxicity of the investigated compounds. Therefore, ROS level analysis constitutes an important element in the evaluation of the potential mechanisms of action of novel anticancer derivatives and their selectivity toward cancer cells.

**Figure 5:**
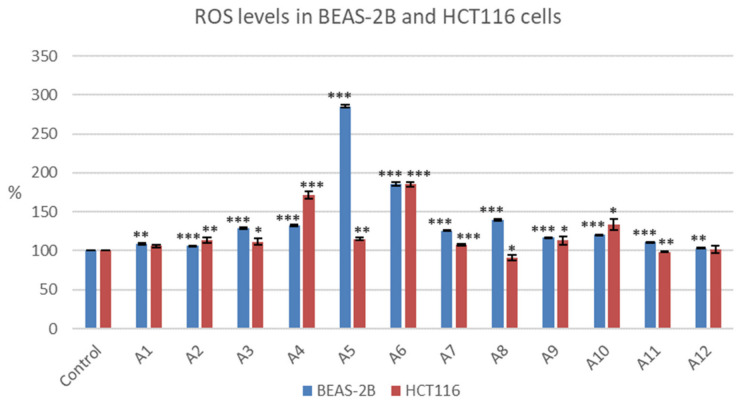
Reactive oxygen species (ROS) levels in BEAS-2B and HCT116 cells following 24 h treatment with compounds **A1–A12** at a concentration of 100 µM. Intracellular ROS levels were determined by flow cytometry using CellROX™ Green reagent. Results are presented as percentage relative to the control group (100%) and expressed as mean ± SD. Statistical significance was determined using an unpaired two-tailed Welch’s *t*-test. * *p* < 0.05, ** *p* < 0.01, *** *p* < 0.001 compared to the control group.

To evaluate the effects of the investigated compounds on oxidative stress levels, ROS production was analyzed in HCT116 and BEAS-2B cells following 24 h incubation with compounds **A1–A12** at a concentration of 100 µM. ROS levels were determined by flow cytometry using the CellROX™ Green dye (Thermo Fisher Scientific, Waltham, MA, USA). The obtained results are presented in Figure 5. Statistical significance was defined as follows: * *p* < 0.05, ** *p* < 0.01, and *** *p* < 0.001, whereas the absence of asterisks indicated no statistically significant differences compared to the control group. Representative flow cytometric histograms of ROS generation are included in the Supplementary Materials (Figure S5).

Analysis of reactive oxygen species (ROS) levels demonstrated that most of the investigated compounds induced increased oxidative stress in both HCT116 and BEAS-2B cells; however, the intensity of this effect depended on the type of compound and the cell line examined. Increased ROS levels were particularly evident for compounds **A4–A8**, whereas derivatives **A9–A12** induced more moderate changes.

The strongest increase in ROS levels in BEAS-2B cells was observed following treatment with compound **A5**, for which ROS levels reached approximately 280% relative to the control. Markedly elevated ROS levels in non-cancerous cells were also induced by compounds **A4**, **A6**, **A7**, and **A8**. These findings suggest that some phenothiazine derivatives may induce strong oxidative stress independently of the neoplastic status of the cells, which may be associated with their toxicity toward normal cells.

In HCT116 cells, the highest ROS levels were observed following incubation with compounds **A4** and **A6**. Compounds **A3**, **A7**, and **A10** also caused a significant increase in reactive oxygen species production, although this effect was less pronounced. At the same time, for some derivatives, particularly **A8**, different response profiles were observed between cancerous and non-cancerous cells. **A8** induced a marked increase in ROS levels in BEAS-2B cells, whereas in HCT116 cells ROS levels remained comparable to the control.

It is also noteworthy that derivatives containing the AZT moiety (**A9–A12**), which exhibited the highest pro-apoptotic and cytotoxic activity against HCT116 cells, did not induce ROS production as strongly as compounds **A4–A8**. This suggests that oxidative stress is not the sole or dominant mechanism responsible for their anticancer activity. In the case of **A9–A12**, mechanisms associated with DNA damage, disruption of cell cycle progression, and apoptosis induction appear to be more probable.

Particular attention should be paid to compound **A9**, which despite its very strong pro-apoptotic activity and favorable cytotoxicity profile induced only a moderate increase in ROS levels in both HCT116 and BEAS-2B cells. This may indicate a more selective mechanism of action not based solely on nonspecific induction of oxidative stress. In contrast, **A5**, despite very strong ROS induction, also exhibited higher toxicity toward normal cells, which may limit its potential therapeutic application. Overall, the obtained results suggest that the investigated phenothiazine derivatives may induce oxidative stress as one of the components of their anticancer mechanism of action.

### 2.7. Cell Cycle

**Figure 6:**
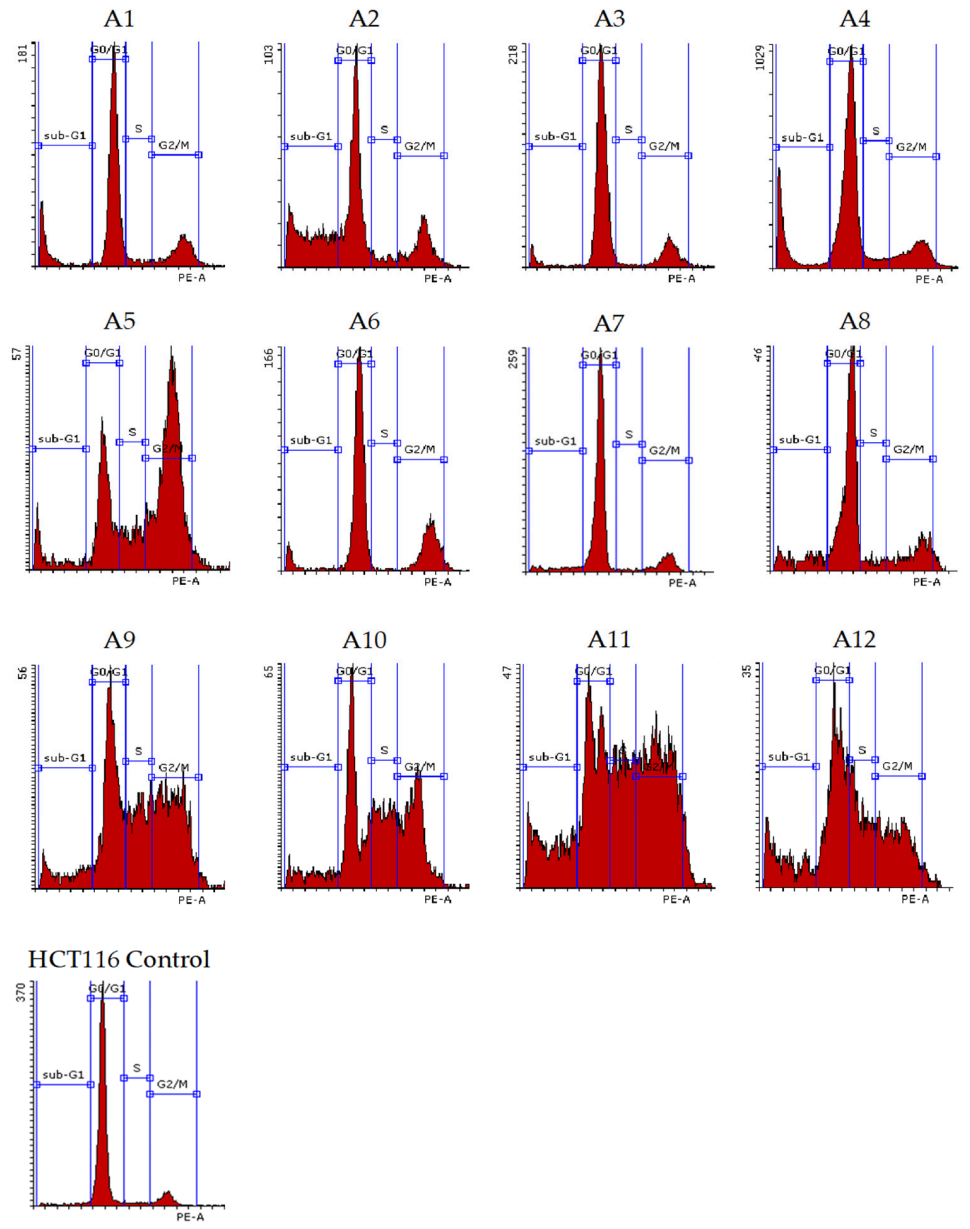
Representative flow cytometry histograms of cell cycle distribution in HCT116 cells treated with compounds **A1–A12**.

**Figure 7:**
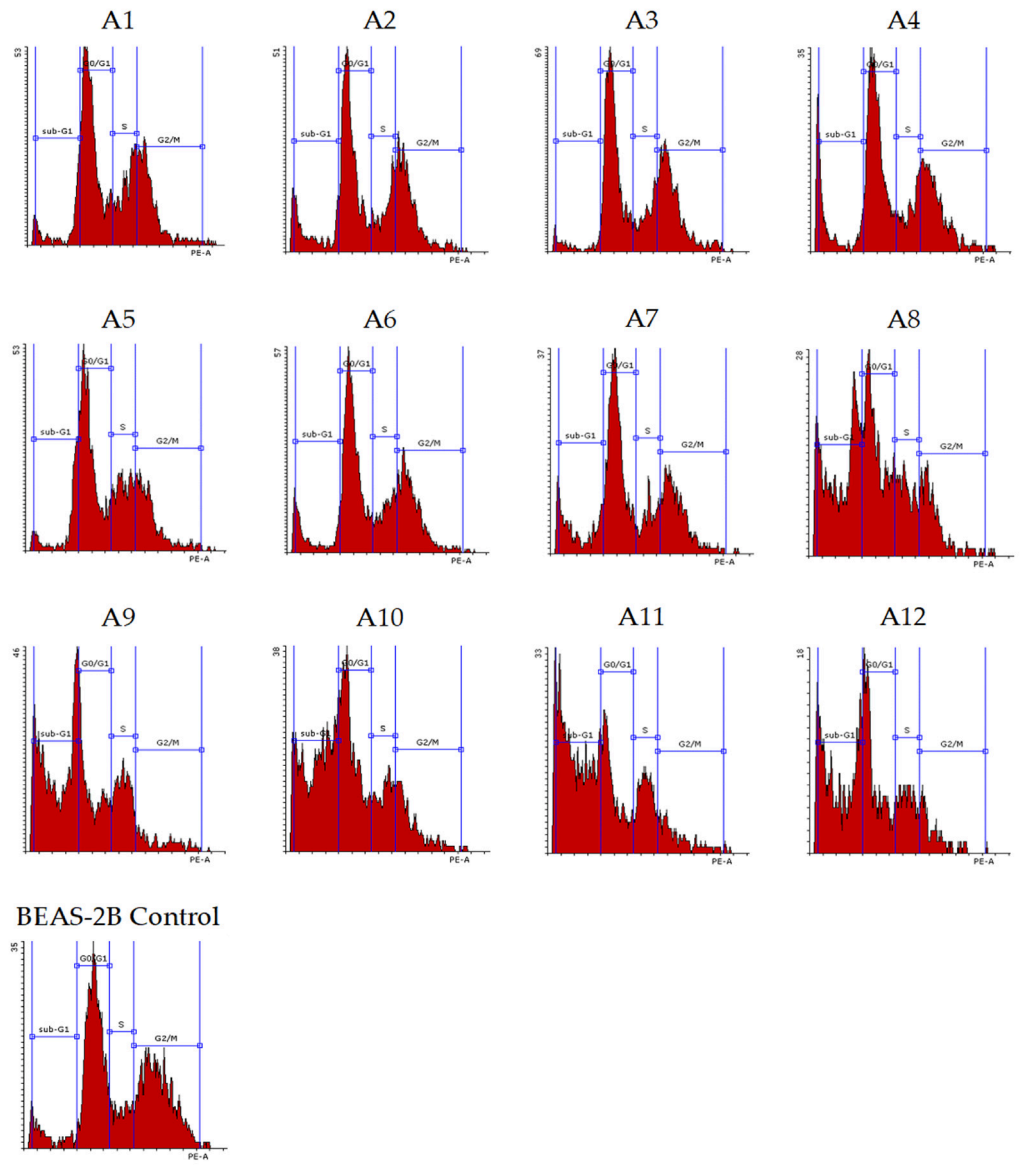
Representative flow cytometry histograms of cell cycle distribution in BEAS-2B cells treated with compounds **A1–A12**.

**Figure 8:**
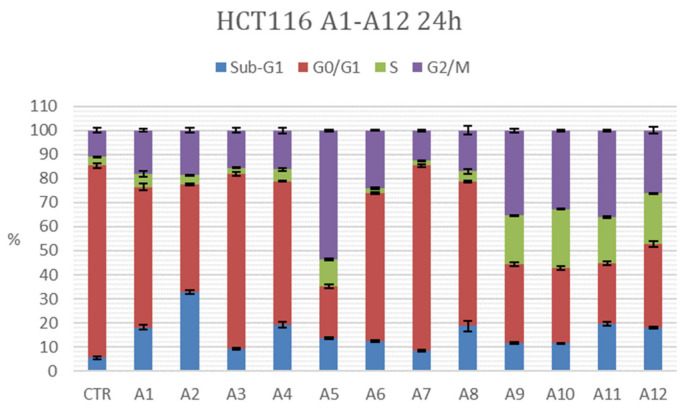
Cell cycle analysis of HCT116 cells following 24 h treatment with compounds **A1**–**A12** at a concentration of 100 µM. The distribution of cells in the sub-G1, G0/G1, S, and G2/M phases was determined by flow cytometry after propidium iodide staining. Results are presented as mean ± SD.

**Figure 9:**
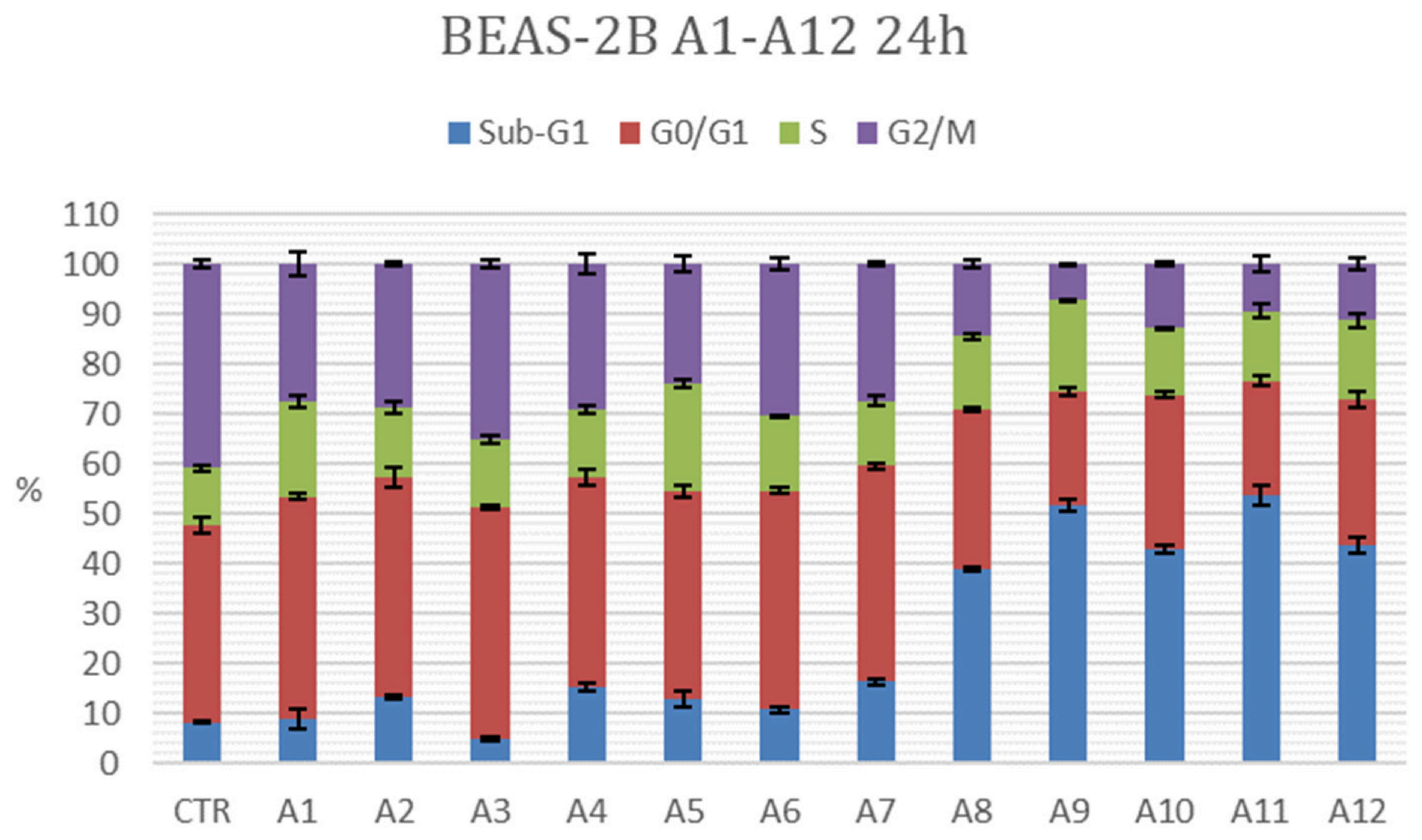
Cell cycle analysis of BEAS-2B cells following 24 h treatment with compounds **A1–A12** at a concentration of 100 µM. The distribution of cells in the sub-G1, G0/G1, S, and G2/M phases was determined by flow cytometry after propidium iodide staining. Results are presented as mean ± SD.

Cell cycle analysis is one of the fundamental methods used to evaluate the mechanism of action of potential anticancer compounds, as it enables assessment of the effects of investigated substances on cell proliferation and identification of the cell cycle phases that are particularly susceptible to their activity. Many anticancer agents induce cell cycle arrest at specific phases, most commonly G0/G1, S, or G2/M, which leads to inhibition of cancer cell proliferation through activation of cell cycle checkpoints and restriction of cell progression to subsequent stages of division [94]. Extensive and short-term DNA damage may activate apoptosis, whereas prolonged and mild damage induces cellular senescence [94]. Additionally, an increase in the sub-G1 population is widely recognized as an indicator of DNA fragmentation and apoptotic cell death [95]. To evaluate the effects of the obtained compounds on cell cycle progression and induction of cell death, cytometric analysis was performed using HCT116 and BEAS-2B cells following 24 h incubation with the investigated compounds at a concentration of 100 µM. The high concentration of the analyzed compounds was applied in mechanistic studies to induce measurable biological changes, enabling assessment of the potential anticancer mechanisms of action of the investigated derivatives. The obtained results are presented in Figure 6, Figure 7, Figure 8 and Figure 9. Quantitative cell cycle distribution data for HCT116 and BEAS-2B cells are provided in the Supplementary Materials as Figures S6 and S7, respectively, to facilitate detailed comparison of changes in individual cell cycle phases.

Cell cycle analysis demonstrated that the investigated compounds induced marked disturbances in cell cycle progression in both HCT116 cancer cells and non-cancerous BEAS-2B cells; however, the nature of the observed changes depended on the structure of the compound and the presence of the AZT moiety. Particularly interesting differences were observed between classical phenothiazine derivatives (**A1–A8**) and AZT-containing hybrids (**A9–A12**), which exhibited distinct activity profiles.

In HCT116 cells, most derivatives lacking the AZT moiety caused predominance of the G0/G1 population with only a slight increase in the sub-G1 fraction. Against this background, compounds **A2** and **A5** were clearly distinguishable. Compound **A2** induced a very strong increase in the sub-G1 population, exceeding 30%, accompanied by a reduction in the proportion of cells in the G0/G1 phase. This may indicate intense induction of DNA fragmentation and cell death occurring at an early stage of compound activity. In contrast, **A5** was characterized by a marked increase in the proportion of cells in the S and G2/M phases together with a moderate increase in the sub-G1 fraction, which may primarily indicate cell cycle arrest associated with disruption of DNA replication and activation of cell cycle checkpoints.

An even more characteristic profile was observed for hybrids containing the AZT moiety. Compounds **A9–A12** induced simultaneous increases in the populations of cells in the S and G2/M phases together with an increase in the sub-G1 fraction, accompanied by a reduction in the G0/G1 fraction, which may indicate coexistence of cell cycle arrest and apoptosis activation. This effect is consistent with previous literature reports concerning AZT, according to which this compound induces cell cycle arrest in the S and G2/M phases through induction of DNA damage and disruption of telomerase activity [38,40,44]. Studies discussed in the theoretical background demonstrated that AZT may lead to increased expression of γ-H2AX, a marker of genetic material damage, activation of Chk1/Chk2, and activation of the p53-p21 pathways, resulting in cell cycle arrest and induction of apoptosis [38,40,42,43,47]. The obtained results suggest that a similar mechanism may also occur in the case of the investigated **A9–A12** hybrids.

In BEAS-2B cells, a different pattern of changes was observed. Compounds **A9–A12** induced a very strong increase in the sub-G1 population, particularly for **A9** and **A11**, where it exceeded 50%. At the same time, the proportion of cells in the G2/M phase was markedly lower than in HCT116 cells. This may suggest that in non-cancerous cells, induction of cell death occurs more rapidly, without prolonged cell cycle arrest. In the case of **A2** and **A5**, the changes observed in BEAS-2B cells were less pronounced than in HCT116 cells, which may indicate partial selectivity of these compounds toward cancer cells.

Overall, the obtained results suggest that the investigated compounds affect cell cycle regulation through disruption of cell progression through the S and G2/M phases and activation of cell cycle checkpoints, which may be associated with induction of DNA damage and impairment of DNA replication processes.

### 2.8. Level of Apoptosis

Apoptosis plays a key role in maintaining tissue homeostasis through the elimination of damaged, mutated, or unnecessary cells. Disturbances in the mechanisms regulating programmed cell death are among the characteristic features of cancer and may lead to uncontrolled proliferation, disease progression, and the development of therapy resistance [96]. For this reason, the ability of potential anticancer compounds to induce apoptosis represents one of the most important parameters used to evaluate their biological activity.

The apoptotic process is characterized by a series of biochemical and morphological changes, including chromatin condensation, DNA fragmentation, loss of plasma membrane asymmetry, and formation of apoptotic bodies. In contrast to necrosis, apoptosis occurs in an orderly manner and usually does not induce a strong inflammatory response [96]. In in vitro studies, flow cytometric analysis of apoptosis using annexin V and propidium iodide enables discrimination between viable cells, cells in early and late apoptosis, and necrotic cells [97].

**Figure 10:**
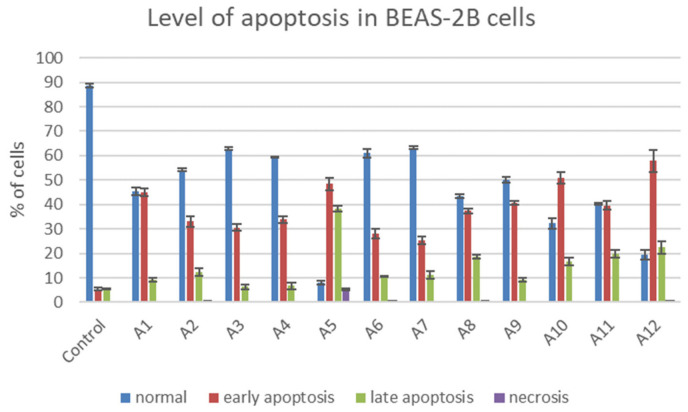
Apoptosis analysis of BEAS-2B cells following 24 h treatment with compounds **A1–A12** at a concentration of 100 µM. The percentages of viable cells, early apoptotic cells, late apoptotic cells, and necrotic cells were determined by flow cytometry using Annexin V/propidium iodide staining. Results are presented as mean ± SD.

**Figure 11:**
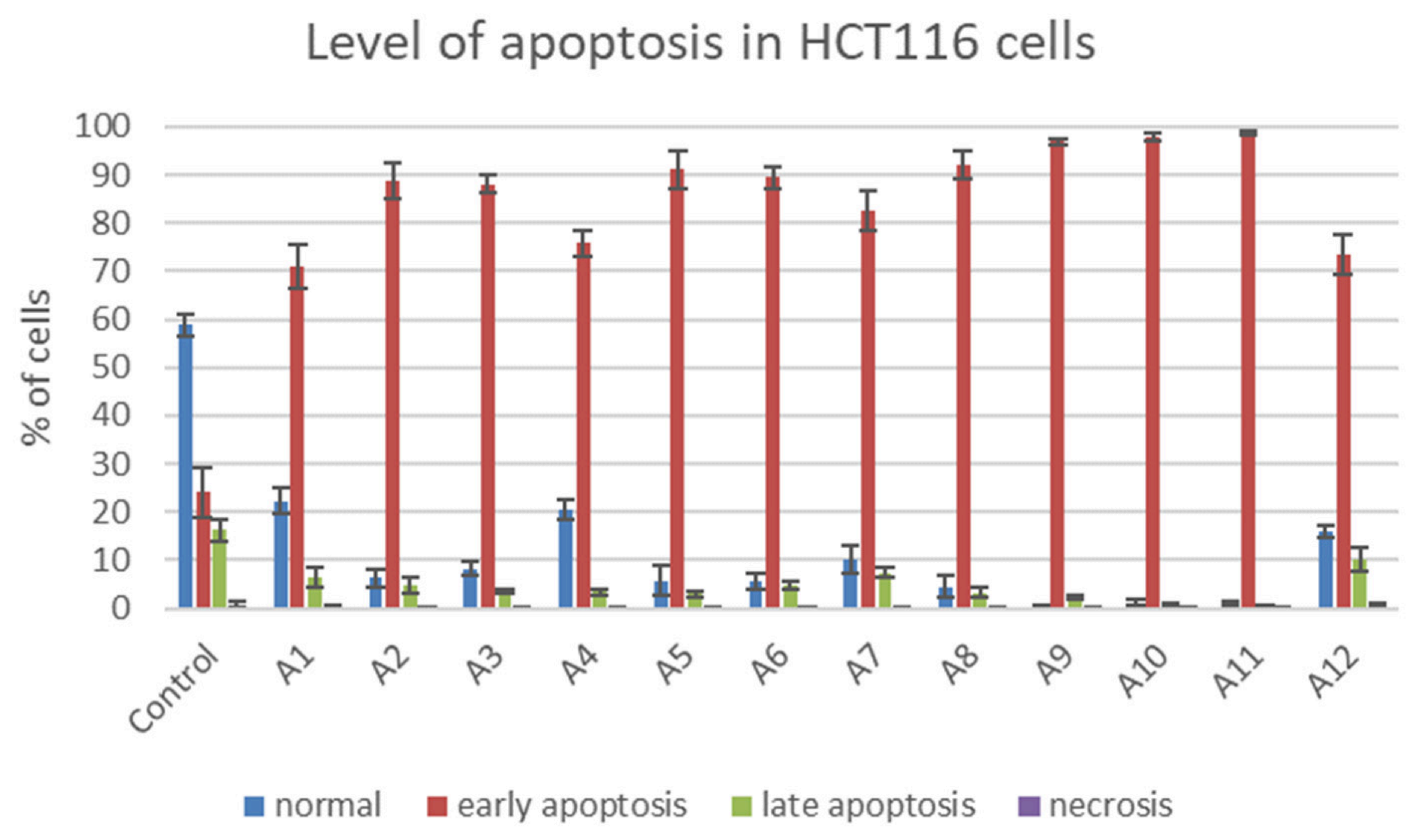
Apoptosis analysis of HCT116 cells following 24 h treatment with compounds **A1–A12** at a concentration of 100 µM. The percentages of viable cells, early apoptotic cells, late apoptotic cells, and necrotic cells were determined by flow cytometry using Annexin V/propidium iodide staining. Results are presented as mean ± SD.

To evaluate the effects of the investigated derivatives on the induction of programmed cell death, apoptosis analysis was performed in HCT116 and BEAS-2B cells following 24 h incubation with compounds **A1–A12** at a concentration of 100 µM. Particular attention was paid to differences between cancerous and non-cancerous cells, which enabled assessment of the potential anticancer selectivity of the analyzed derivatives. The obtained results, presented as the mean of three independent experiments together with standard deviation (±SD), are shown in Figure 10 and Figure 11 and are available in the Supplementary Materials (Figure S8).

Apoptosis analysis in HCT116 cells demonstrated that all investigated compounds **A1–A12** induced a marked increase in the percentage of cells undergoing early apoptosis compared to the control, accompanied by a substantial reduction in the number of viable cells. Following treatment with the investigated compounds, the proportion of viable cells decreased markedly, while the apoptotic population significantly increased. The strongest pro-apoptotic activity was observed for compounds **A9–A11**, which caused an almost complete shift in the cell population toward early apoptosis. In the case of these derivatives, the percentage of cells in early apoptosis reached approximately 90–95%, whereas the proportion of viable cells was minimal. These findings indicate very strong anticancer activity of these compounds against colorectal cancer cells.

The obtained results also suggest that the presence of the azidothymidine moiety may enhance the ability of the investigated compounds to induce apoptotic cell death. Compared to earlier phenothiazine derivatives (**A1–A8**), compounds containing AZT exhibited markedly stronger apoptotic activity and a greater reduction in the number of viable cells. For most investigated compounds, early apoptosis was the predominant form of cell death, whereas the contribution of late apoptosis and necrosis remained relatively low. This suggests that the analyzed derivatives primarily induce controlled cell death rather than nonspecific cellular damage leading to necrosis. Such an activity profile is advantageous from the perspective of potential anticancer application, since apoptosis is considered a more desirable mechanism of cancer cell elimination than necrosis, which may intensify the inflammatory response. Among the derivatives lacking the AZT moiety, particularly interesting results were obtained for **A2**, **A5**, and **A8**, which also caused a marked increase in the proportion of apoptotic cells. However, their activity was slightly weaker than that observed for derivatives **A9–A11**. These findings suggest that the phenothiazine core itself possesses the ability to induce apoptosis, whereas conjugation with AZT may further enhance this effect. Overall, the results indicate that the main mechanism underlying the cytotoxic activity of the investigated derivatives against HCT116 cells is apoptosis induction, and the most promising candidates for further studies appear to be compounds **A9–A11**, particularly **A9**, which combines very strong pro-apoptotic activity with a favorable selectivity profile observed in other biological assays. In BEAS-2B cells, the response profile to the investigated compounds was more heterogeneous than in HCT116 cells. For most derivatives, a relatively high percentage of viable cells was maintained, suggesting lower toxicity toward non-cancerous cells. Compound **A2** was particularly interesting, as despite its strong pro-apoptotic activity against HCT116 cells, it induced a markedly weaker effect in BEAS-2B cells. These findings may indicate partial selectivity of this derivative toward cancer cells and are consistent with the Alamar Blue assay results, where for HCT116 cells after 24 h IC_50_ = 50.75 ± 1.67 µM, whereas for BEAS-2B cells IC_50_ > 100 µM. A different activity profile was observed for compound **A5**, which in BEAS-2B cells induced both high levels of early and late apoptosis, accompanied by a slight increase in necrosis. These results suggest stronger and less selective cytotoxic activity of this derivative toward normal cells. Among the derivatives containing the AZT moiety (**A9–A12**), compound **A9** exhibited the most favorable profile. Despite its very strong pro-apoptotic activity against HCT116 cells, a relatively high proportion of viable cells, approximately 50%, was still maintained in BEAS-2B cells, suggesting a more favorable biological selectivity profile for this derivative. Compound **A11** induced a more moderate effect, resulting in a comparable proportion of viable cells and cells undergoing early apoptosis. In contrast, for **A10** and **A12**, the population of cells in early apoptosis predominated, indicating stronger activity also toward non-cancerous cells.

It is worth emphasizing that some derivatives lacking the AZT moiety, despite clearly inducing apoptosis in HCT116 cells, did not exhibit equally strong cytotoxic effects in other biological assays. This may suggest that the observed activation of apoptotic mechanisms did not always translate into sustained inhibition of cell viability or that the effect was more transient in nature.

### 2.9. In Silico ADME and Drug-likeness Evaluation

To further characterize the analyzed compounds, selected physicochemical and pharmacokinetic parameters predicted using the SwissADME platform were evaluated (Table 3) [98,99]. The analysis included molecular weight (MW), topological polar surface area (TPSA), lipophilicity (Consensus LogP), aqueous solubility (ESOL LogS), gastrointestinal absorption, bioavailability score, and compliance with Lipinski’s rule of five, which is commonly used to estimate the drug-likeness and oral bioavailability of small molecules.

Clear differences were observed between the precursor compound **A2** and the AZT-containing hybrids **A9–A12**. Compound **A2** was characterized by markedly lower molecular weight and TPSA values, which are parameters strongly associated with membrane permeability and passive diffusion. This compound also showed high predicted gastrointestinal absorption and better compliance with Lipinski’s rule of five compared with the hybrid derivatives. These features may partially explain the relatively rapid cytotoxic effect of **A2** observed after 24 h incubation.

In contrast, the AZT-containing hybrids were characterized by substantially higher molecular weight and polarity, reflected by increased TPSA values. All compounds from this group showed molecular weights above 550 g/mol and TPSA values of approximately 151–176 Å^2^. These properties are consistent with the low predicted gastrointestinal absorption and low bioavailability score observed for **A9–A12**. Thus, although the hybrids demonstrated stronger and more sustained anticancer activity in biological assays, their predicted oral bioavailability appears limited.

Lipophilicity and solubility varied within the hybrid series depending on the substituent attached to the aromatic system. Compound **A9**, containing hydrogen as the substituent, showed the lowest lipophilicity among the AZT-containing hybrids, with a Consensus LogP value of 1.76, and the most favorable predicted aqueous solubility according to the ESOL model (LogS −4.95). These features may support dissolution; however, the high molecular weight and elevated TPSA still strongly limit predicted intestinal absorption.

In compound **A10**, the presence of fluorine slightly increased lipophilicity, with a Consensus LogP value of 2.12, while maintaining the same TPSA value as **A9**. This was accompanied by a slight decrease in predicted solubility, suggesting a modest shift toward a more lipophilic profile. However, this change was not sufficient to improve the predicted bioavailability class, as **A10** was still characterized by low gastrointestinal absorption and the same low bioavailability score as the other hybrids.

Compound **A11**, bearing a chlorine substituent, showed a more pronounced increase in lipophilicity and lower predicted solubility (ESOL LogS −5.55), with a shift toward poorer solubility also indicated by other solubility models. Although the higher lipophilicity of **A11** could theoretically support membrane interactions, this potential advantage is counterbalanced by reduced solubility and persistently high TPSA, which together maintain its unfavorable predicted oral bioavailability profile.

The SCH_3_-containing derivative **A12** showed the highest TPSA value in the hybrid series (176.31 Å^2^), together with a molecular weight above 600 g/mol and lipophilicity comparable to **A11**. Its predicted solubility was also lower than that of **A9** and **A10**. Therefore, despite its pronounced long-term biological activity in cellular assays, **A12** appears to be the least favorable compound among the hybrids in terms of predicted oral bioavailability, mainly due to its high polarity, increased molecular size, and limited solubility.

Overall, the in silico analysis indicates that the substituent mainly modulates the balance between lipophilicity and aqueous solubility, but does not overcome the principal limitations shared by **A9–A12**, namely high molecular weight, elevated TPSA, low predicted gastrointestinal absorption, and low bioavailability score. Nevertheless, all analyzed compounds retained measurable biological activity in cellular assays, indicating that their unfavorable predicted oral bioavailability did not prevent cellular activity under the applied in vitro conditions. Importantly, none of the analyzed compounds generated PAINS or Brenk alerts, suggesting the absence of structural motifs commonly associated with nonspecific or problematic biological activity.

## 3. Materials and Methods

Melting points were determined using a Boetius melting point apparatus in open capillary tubes and are uncorrected. Nuclear magnetic resonance (NMR) spectra were recorded on a Bruker Avance 600 spectrometer (Bruker, Billerica, MA, USA). ^1^H NMR spectra were acquired at 600 MHz, whereas ^13^C NMR spectra were measured at 150 MHz. DMSO-d6 was used as the solvent in all experiments. Chemical shifts (δ) are reported in parts per million (ppm). High-resolution mass spectra (HRMS) were obtained under electron ionization (EI) conditions using a Bruker Impact II mass spectrometer (Bruker, Billerica, MA, USA). The ^1^H NMR, ^13^C NMR, and HRMS spectra are provided in the Supplementary Materials. Thin-layer chromatography (TLC) analyses were carried out on aluminum plates coated with neutral alumina 60 F_254_ (type E; Merck, 1.05581), employing dichloromethane (CH_2_Cl_2_) as the developing solvent. The parent quinobenzothiazines—6*H*-quino[3,2-b]benzo[1,4]thiazine (**A1**), 6*H*-9-fluoroquino[3,2-b]benzo[1,4]thiazine (**A2**), 6*H*-9-chloroquino[3,2-b]benzo[1,4]thiazine (**A3**), and 6*H*-9-methylthioquino[3,2-b]benzo[1,4]thiazine (**A4**) were synthesized according to previously reported procedures [75]. Subsequently, these compounds were converted into their corresponding 6-propynyl derivatives (**A5–A8**) following the methodology described in the literature [70].

### 3.1. General Procedure for the Synthesis of Compounds **A9–A12**

0.75 mmol of AZT was added to a solution of the appropriate 6-propargylquinobenzothiazine (**A5–A8**) in 10 ml of DMF. Solutions of 0.1 mmol of sodium ascorbate in 1 ml of water and 0.05 mmol of CuSO_4_ × 5H_2_O, also in 1 ml of water, were then prepared. Both solutions were mixed and immediately poured into the reaction mixture. The mixture was stirred at room temperature for 48 h. After this time, the mixture was poured into 50 ml of cold water, the precipitate was drained off, washed with water and air-dried. The crude products were purified on a chromatographic column packed with Al_2_O_3_, using a 5:1 chloroform-ethanol mixture as the solvent.

1-(4-(4-((Quino[3,2-b]benzo[1,4]thiazin-6-yl)methyl)-1*H*-1,2,3-triazol-1-yl)-5-(hydroxymethyl)tetrahydrofuran-2-yl)-5-methylpyrimidine-2,4(1*H*,3*H*)-dione (**A9**):

Yield: 75%. M.p.: 265–266 °C. ^1^H NMR (600 MHz, CDCl_3_) δ: 1.93 (s. 3H, CH_3_), 2.96–3.02 (m, 2H), 3.73–3.76 (m, 2H), 3.80–3.82 (m, 1H), 4.47–4.48 (m, 1H), 5.50–5.52 (m, 1H), 6.25 (t, 1H, *J* = 6.6 Hz), 7.38–7.44 (m, 3H, H-7, H-9, H-10), 7.53–7.57 (m, 3H, H-1, H-3, H-8), 7.70 (s, 1H, H-12), 7.90 (s, 1H, CH), 7.94 (d, 1H, H-4, *J* = 7.8 Hz), 8.03 (s, 1H), 8.7 (s, 1H, N-H). ^13^C NMR (150 MHz, CDCl_3_) δ: 12.48, 37.48, 58.19, 59.51, 61.70, 85.34, 89.18, 111.30, 111.38, 124.46, 125.03, 126.48, 126.70, 127.93, 128.26, 129.80, 129.83, 131.31, 133.36, 134.61, 137.81, 137.98, 142.88, 150.23, 150.29, 151.48, 163.41, 165.29. HR MS (ESI) calcd for: C_28_H_26_N_7_O_4_S [M + H]^+^: 556.1767, found: 556.1773.

1-(4-(4-((9-Fluoroquino[3,2-b]benzo[1,4]thiazin-6-yl)methyl)-1*H*-1,2,3-triazol-1-yl)-5-(hydroxymethyl)tetrahydrofuran-2-yl)-5-methylpyrimidine-2,4(1*H*,3*H*)-dione (**A10**):

Yield: 73%. M.p.: 249–250 °C. ^1^H NMR (600 MHz, DMSO-d6) δ: 1.79 (s, 3H, CH_3_), 2.57–2.60 (m, 1H), 2.66–2.69 (m, 1H), 3.56–3.38 (m, 1H), 3.64–3.66 (m, 1H), 4.16–4.18 (m, 1H), 5.27 (s, 1H), 5.35–5.40 (m, 2H), 6.43 (t, 1H, *J* = 6.6 Hz), 7.04–7.07 (m, 1H, H-7), 7.17–7.19 (m, 1H, H-8), 7.34 (t, 1H, H-2), 7.37–7.40 (m, 1H, H-10), 7.56–7.59 (m, 1H, H-3), 7.69–7.70 (m, 1H, H-1), 7.48 (d, 1H, H-4, *J* = 8.4 Hz), 7.80 (s, 1H, H-12), 8.02 (s, 1H), 8.30 (s, 1H, CH), 11.37 (s, 1H, N-H). ^13^C NMR (150 MHz, DMSO-d6) δ: 12.73, 19.03, 37.63, 59.73, 61.22, 84.35, 84.94, 110.05, 113.88 (d, *J*_C-F_ = 25.5 Hz), 114.63 (d, *J*_C-F_ = 22.0 Hz), 116.84, 117.97 (d, *J*_C-F_ = 8.1 Hz), 121.49 (d, *J*_C-F_ = 8.4 Hz), 124.99, 125.14, 126.14, 127.01, 127.46, 130.10, 132.80, 136.65, 137.60 (d, *J*_C-F_ = 1.8 Hz), 144.11, 145.44, 150.91, 151.36, 158.40 (d, *J*_C-F_ = 239.91 Hz), 164.18. HR MS (ESI) calcd for: C_28_H_25_FN_7_O_4_S [M + H]^+^: 574.1673, found: 574.1673.

1-(4-(4-((9-Chloroquino[3,2-b]benzo[1,4]thiazin-6-yl)methyl)-1*H*-1,2,3-triazol-1-yl)-5-(hydroxymethyl)tetrahydrofuran-2-yl)-5-methylpyrimidine-2,4(1*H*,3*H*)-dione (**A11**):

Yield: 71%. M.p.: 253–254 °C. ^1^H NMR (600 MHz, CDCl_3_) δ: 1.93 (s. 3H, CH_3_), 2.85–2.98 (m, 2H), 3.72–3.76 (m, 2H), 3.98 (d, 1H, *J* = 12 Hz), 4.43–4.44 (m, 1H), 5.35–5.39 (m, 1H), 6.21 (t, 1H, *J* = 13.2 Hz), 7.05 (s, 1H, H-7), 7.08 (d, 1H, H-10, *J* = 8.4 Hz), 7.34 (t, 1H, H-2, *J* = 7.2 Hz), 7.36–7.38 (m, 1H, H8), 7.56–7.58 (m, 2H, H-1, H-3), 7.68 (s, 1H, H-12), 7.78 (d, 1H, H-4, *J* = 4.1 Hz), 7.96 (s, 1H), 8.03 (s, 1H, CH), 8.54 (s, 1H, N-H). ^13^C NMR (150 MHz, CDCl_3_) δ: 12.46, 36.53, 37.45, 59.36, 61.78, 85.29, 89.28, 111.31, 117.54, 117.83, 121.66, 124.86, 124.99, 125.97, 126.01, 126.47, 127.76, 128.24, 128.51, 129.97, 132.53, 137.94, 139.69, 144.70, 150.17, 151.21, 162.58, 163.30. HR MS (ESI) calcd for: C_28_H_25_ClN_7_O_4_S [M + H]^+^: 590.1377, found: 590.1385.

1-(4-(4-((9-Methylthioquino[3,2-b]benzo[1,4]thiazin-6-yl)methyl)-1H-1,2,3-triazol-1-yl)-5-(hydroxymethyl)tetrahydrofuran-2-yl)-5-methylpyrimidine-2,4(1H,3H)-dione (**A12**):

Yield: 69%. M.p.: 270–271 °C. ^1^H NMR (600 MHz, DMSO-d6) δ: 1.78 (s, 3H, CH_3_), 2.25–2.29 (m, 2H), 2.47 (s, 3H, SCH_3_), 3.60–3.64 (m, 2H), 3.80–3.83 (m, 1H), 4.39–4.42 (m, 1H), 4.96 (s, 1H), 5.24 (t, 1H, *J* = 5.4 Hz), 6.10 (t, 1H, *J* = 6.6 Hz), 7.17–7.22 (m, 3H, H-7, H-8, H-10), 7.36 (t, 1H, H-2, *J* = 7.2 Hz), 7.58 (t, 1H, H-3, *J* = 7.8 Hz), 7.69–7.72 (m, 3H, H-4, H-12, H-pyrim), 8.06 (s, 1H, CH), 11.35 (s, 1H, N-H). ^13^C NMR (150 MHz, DMSO-d6) δ: 12.71, 15.90, 35.27, 36.63, 60.61, 61.25, 83.81, 84.42, 109.97, 117.19, 117.28, 120.72, 124.83, 125.12, 126.16, 126.62, 127.11, 127.42, 130.12, 132.99, 133.06, 136.53, 137.84, 145.24, 150.87, 151.14, 164.18. HR MS (ESI) calcd for: C_29_H_28_N_7_O_4_S_2_ [M + H]^+^: 602.1644, found: 602.1651.

### 3.2. Cell Culture

The HCT116 (ATCC CCL-247), HT-29 (ATCC HTB-38), and BEAS-2B (ATCC CRL-3588) cell lines used in this study were obtained from the American Type Culture Collection (ATCC, Manassas, VA, USA). The BEAS-2B human bronchial epithelial cell line was cultured in RPMI-1640 medium, while the HCT116 and HT-29 colorectal cancer cell lines were maintained in DMEM medium. All culture media were supplemented with 10% fetal bovine serum (FBS) and 1% penicillin/streptomycin solution. Cells were cultured under standard conditions at 37 °C in a humidified atmosphere containing 5% CO_2_. The culture medium was routinely replaced, and cells were passaged after reaching appropriate confluence using trypsinization.

### 3.3. Morphological Analysis

For morphological analysis, cells were seeded into 96-well plates at a density of 1 × 10^4^ cells per well and incubated for 24 h under standard culture conditions (37 °C, 5% CO_2_, humidified atmosphere) to allow proper attachment. After this period, compounds from the **A1–A12** series were added to the culture medium at a final concentration of 100 µM. Cells cultured without the tested compounds were used as the control group. Following 24 h incubation with the analyzed derivatives, cellular morphology was evaluated using a JuLI™ FL Live fluorescence microscope (NanoEntek, Martinsried, Germany). Microscopic observations included assessment of cell shape, degree of confluence, cellular arrangement, and attachment to the culture surface.

### 3.4. Alamar Blue Assay

The effect of the analyzed compounds on cell viability was assessed using the Alamar Blue assay. Cells were seeded into 96-well plates and maintained for 24 h under standard incubation conditions (37 °C, 5% CO_2_, humidified atmosphere) to enable cell attachment and stabilization. After incubation, the culture medium was supplemented with the tested compounds at concentrations ranging from 6.25 to 100 µM. Cells cultured in medium without the addition of the analyzed derivatives were used as the control and defined as 100% viable.

Following treatment, Alamar Blue reagent (Invitrogen, Thermo Fisher Scientific, Waltham, MA, USA) was added to each well according to the manufacturer’s protocol, and the plates were incubated for an additional period protected from light. Fluorescence was subsequently measured using a microplate reader. All concentrations were tested in triplicate, and cell viability was expressed as a percentage relative to untreated control cells.

IC_50_ values were calculated from dose–response curves obtained for individual compounds. For each technical replicate, the IC_50_ was estimated by linear interpolation between two neighboring concentrations corresponding to viability values above and below 50%. Mean IC_50_ values and standard deviations were then determined from three independent calculations. In cases where the viability threshold of 50% was not reached within the tested concentration range, approximate extrapolation was applied only for comparative estimation purposes.

### 3.5. RT-qPCR Analysis

BEAS-2B and HCT116 cells were grown in 6-well culture plates under standard incubation conditions (37 °C, 5% CO_2_, humidified atmosphere). BEAS-2B cells were maintained in RPMI-1640 medium, whereas HCT116 cells were cultured in DMEM medium. Both media were supplemented with 10% fetal bovine serum (FBS) and 1% penicillin/streptomycin solution. Cells were seeded at a density of 2 × 10^5^ cells per well and allowed to attach for 24 h before treatment. Subsequently, the analyzed compounds were added at a final concentration of 100 µM and incubated with the cells for an additional 24 h.

After treatment, cells were washed with PBS, detached by trypsinization, collected by centrifugation, and suspended in Fenozol reagent. Samples were stored at −20 °C until further analysis. Total RNA was isolated using the Total RNA Mini kit (A&A Biotechnology, Gdynia, Poland) following the manufacturer’s recommendations. RNA quantity and purity were evaluated spectrophotometrically using a NanoDrop 2000 instrument (Thermo Fisher Scientific, Waltham, MA, USA). Complementary DNA (cDNA) synthesis was performed using the NG dART RT kit (EURx, Gdańsk, Poland).

Expression levels of *BCL2*, *AIFM2*, *MDM2*, *OGG1*, *IL6*, and *IL8* were determined by real-time quantitative PCR using Brilliant III Ultra Fast SYBR Green QPCR Master Mix and gene-specific primers (Table 4). The *RPL41* gene was used as an internal reference control. Amplification reactions were carried out using the CFX96 Real-Time PCR Detection System (Bio-Rad, Hercules, CA, USA). Data acquisition and preliminary analysis were performed using CFX Maestro software (version 2.3), while further calculations were conducted in Microsoft Excel.

Relative changes in gene expression were determined using the 2^−ΔΔCt^ method with untreated cells used as the reference group [83]. The obtained data are presented as mean ± standard deviation (SD) from three independent experiments. Statistical evaluation was carried out using ΔCt values derived from RT-qPCR analysis (*n* = 3). Comparisons between control and treated groups were performed using an unpaired two-tailed Welch’s *t*-test, selected due to its robustness in analyses not assuming equal variances between compared datasets. Differences were considered statistically significant at *p* < 0.05. Statistical significance was marked as follows: * *p* < 0.05, ** *p* < 0.01, and *** *p* < 0.001, whereas results with *p* ≥ 0.05 were regarded as not statistically significant.

### 3.6. ROS Analysis

BEAS-2B and HCT116 cells were seeded into 12-well plates at a density of 1 × 10^5^ cells per well and maintained for 24 h under standard incubation conditions (37 °C, 5% CO_2_, humidified atmosphere). BEAS-2B cells were cultured in RPMI-1640 medium, whereas HCT116 cells were maintained in DMEM medium. Both media were supplemented with 10% fetal bovine serum and 1% penicillin/streptomycin. After cell attachment, the analyzed compounds were added at a final concentration of 100 µM and incubated with the cells for an additional 24 h.

Following treatment, cells were collected and stained with CellROX™ Green Reagent (Thermo Fisher Scientific, Waltham, MA, USA) at a final concentration of 5 µM according to the manufacturer’s recommendations. The samples were then analyzed using a FACSAria™ III flow cytometer (Becton Dickinson, Franklin Lakes, NJ, USA).

CellROX™ Green is a fluorogenic probe designed for the detection of intracellular reactive oxygen species in live cells. In its reduced state, the reagent exhibits minimal fluorescence; however, after oxidation by ROS, it produces a strong fluorescent signal and binds to nucleic acids. The intensity of fluorescence correlates with the intracellular oxidative stress level, allowing quantitative assessment of ROS generation by flow cytometry [100]. Comparisons between control and treated groups were performed using an unpaired two-tailed Welch’s *t*-test, selected due to its robustness in analyses not assuming equal variances between compared datasets. Differences were considered statistically significant at *p* < 0.05. Statistical significance was marked as follows: * *p* < 0.05, ** *p* < 0.01, and *** *p* < 0.001, whereas results with *p* ≥ 0.05 were regarded as not statistically significant.

### 3.7. Cell Cycle Analysis

BEAS-2B and HCT116 cells were seeded into 6-well plates at a density of 3 × 10^5^ cells per well and maintained for 24 h under standard incubation conditions (37 °C, 5% CO_2_, humidified atmosphere). BEAS-2B cells were cultured in RPMI-1640 medium, whereas HCT116 cells were grown in DMEM medium. Following the initial incubation period, cells were exposed to the analyzed compounds at a final concentration of 100 µM for 24 h.

After treatment, cells were harvested, centrifuged at 3000 rpm for 2 min, and washed with PBS. The resulting cell pellets were fixed in 70% cold ethanol and stored at −20 °C until flow cytometric analysis. Prior to staining, samples were centrifuged again, the ethanol was removed, and the cells were washed twice with PBS. After the final wash, a small volume of PBS was left above the pellet and the cells were incubated with RNase solution (100 µg/mL, Merck KGaA, Darmstadt, Germany) for 20 min at room temperature in the dark to eliminate RNA interference. Subsequently, propidium iodide (PI) solution (100 µg/mL, Merck) was added to each sample.

Cell cycle distribution was analyzed using a FACSAria™ III flow cytometer (Becton Dickinson, Franklin Lakes, NJ, USA) equipped with a 488 nm laser and LP 550/BP 575/25 optical filters. The applied method is based on quantitative measurement of DNA content using propidium iodide, a fluorescent intercalating dye that binds double-stranded DNA. Since fluorescence intensity directly reflects the amount of cellular DNA, it enables discrimination of cell populations corresponding to the G0/G1, S, and G2/M phases of the cell cycle. Cells with fragmented DNA, characteristic of apoptotic processes, were identified as the sub-G1 population [101].

### 3.8. Analysis of Apoptotic Cell Death

BEAS-2B and HCT116 cells were seeded into 12-well plates at a density of 1 × 10^5^ cells per well and cultured for 24 h under standard conditions (37 °C, 5% CO_2_, humidified atmosphere). BEAS-2B cells were maintained in RPMI-1640 medium, whereas HCT116 cells were cultured in DMEM medium. Both media were supplemented with 10% FBS and 1% penicillin/streptomycin. After the initial incubation period, cells were treated with the analyzed compounds at a concentration of 100 µM for an additional 24 h.

Following treatment, cells were harvested, centrifuged at 3000 rpm for 2 min, and washed with PBS. The resulting pellets were resuspended in Annexin V Staining Buffer from the Annexin V-FITC Apoptosis Detection Kit (Invitrogen, Waltham, MA, USA). Subsequently, Annexin V-FITC and propidium iodide (PI) were added to each sample according to the manufacturer’s protocol. Samples were incubated for 20 min at room temperature in the dark and then analyzed using a FACSAria™ III flow cytometer (Becton Dickinson, Franklin Lakes, NJ, USA).

The assay is based on simultaneous staining with Annexin V-FITC and propidium iodide, allowing discrimination between viable, apoptotic, and necrotic cells by flow cytometry. During the early stages of apoptosis, phosphatidylserine residues become exposed on the outer surface of the plasma membrane and can be detected by Annexin V binding. In contrast, propidium iodide penetrates only cells with impaired membrane integrity, characteristic of late apoptotic or necrotic cells. Based on the staining pattern, cells were classified as viable (Annexin V−/PI−), early apoptotic (Annexin V+/PI−), late apoptotic (Annexin V+/PI+), or necrotic (Annexin V−/PI+) [102].

### 3.9. In Silico ADME

In silico physicochemical, pharmacokinetic, and drug-likeness properties of selected compounds were predicted using the SwissADME online platform [98,99]. The chemical structures of the analyzed compounds were converted into SMILES format and submitted to SwissADME using default settings. The evaluated parameters included molecular weight (MW), topological polar surface area (TPSA), lipophilicity expressed as Consensus LogP, aqueous solubility estimated by the ESOL LogS model, predicted gastrointestinal absorption, bioavailability score, and compliance with Lipinski’s rule of five. In addition, PAINS and Brenk alerts were analyzed to identify potential structural motifs associated with nonspecific biological activity or problematic medicinal chemistry properties.

## 4. Conclusions

The conducted studies confirmed that modification of the phenothiazine core through the introduction of a 1,2,3-triazole moiety and conjugation with AZT represents an effective strategy for the design of novel compounds with potential anticancer activity. The obtained derivatives exhibited a multidirectional activity profile involving effects on cell viability, cell cycle progression, apoptosis induction, and modulation of oxidative stress. The results suggest that combining the classical phenothiazine scaffold with the AZT moiety may lead to compounds with a more complex and multitarget mechanism of action than phenothiazine derivatives alone.

Particularly interesting is the observation that derivatives containing the AZT moiety exhibited stronger and markedly faster biological activity than zidovudine itself, as described in the literature. In the case of classical antitelomeric therapy using AZT, the anticancer effect was observed only after reaching critical telomere shortening, which according to the literature data required approximately 20–30 cell divisions [39]. For example, HT-29 cells cultured for 91 days with AZT at a concentration of 125 μM showed only a reduction in the number of population doublings, indicating a relatively slow mechanism of action of AZT alone and raising doubts regarding its efficacy against rapidly proliferating cancers [45]. In contrast, compounds **A9–A11**, after only 24 h incubation at a concentration of 100 μM, induced more than 90% early apoptosis in HCT116 cells and caused marked disturbances in cell cycle progression associated with accumulation of cells in the S and G2/M phases. These findings suggest that conjugation of AZT with the phenothiazine-triazole scaffold may significantly enhance and accelerate anticancer activity compared to zidovudine alone.

A more detailed analysis of the obtained IC_50_ values revealed clear differences between the precursor compounds **A1–A8** and the AZT-containing hybrids **A9–A12**. Overall, the hybrid derivatives showed stronger anticancer activity toward colorectal cancer cells than their non-conjugated counterparts, suggesting that incorporation of the AZT fragment enhanced the biological activity of the analyzed phenothiazine derivatives. These observations are consistent with the proposed role of AZT in promoting mechanisms associated with replication stress, DNA damage response, telomerase inhibition, and apoptosis induction. Within the hybrid series, the substituent present at position 9 appeared to influence both the rate and durability of the observed cytotoxic effect. Compound **A9**, containing hydrogen as the substituent, showed the most favorable balance between anticancer activity and selectivity toward non-cancerous cells. In contrast, **A11** containing chlorine exhibited reduced selectivity, whereas **A12** bearing the SCH_3_ group demonstrated the strongest effect after prolonged incubation. This may be related to the increased lipophilicity and enhanced intracellular retention associated with the SCH_3_ substituent, which could promote the gradual accumulation of replication-associated disturbances during extended exposure. Interesting differences were also observed between compounds **A2** and **A10**, both containing a fluorine substituent. Despite the absence of the AZT fragment, **A2** exhibited relatively strong cytotoxic activity toward HCT116 cells after only 24 h incubation. This may suggest that small electronegative substituents such as fluorine facilitate membrane permeability and rapid interaction with intracellular targets, leading to an earlier reduction in cell viability. In contrast, **A10**, representing the AZT-conjugated analogue of **A2**, showed a more sustained biological effect after prolonged incubation, which may indicate that the presence of the AZT moiety enhances time-dependent mechanisms associated with replication disturbances and cumulative DNA damage. These observations further support the hypothesis that conjugation of phenothiazine derivatives with AZT contributes not only to increased cytotoxicity, but also to prolonged biological activity during extended exposure.

The obtained data also indicate that the activity of the investigated compounds is not associated solely with the induction of oxidative stress, but most likely also involves disturbances in the DNA damage response, modulation of pathways related to cell cycle regulation, and activation of apoptotic mechanisms. The proapoptotic activity of compounds **A9–A12** observed not only in HCT116 cells but also in HT-29 cells suggests that their mechanism of action is not exclusively dependent on functional p53 [103]. This observation indicates that p53-independent mechanisms, such as replication-associated stress, mitochondrial dysfunction, oxidative stress, or alternative DNA damage response pathways, may also contribute to apoptosis induction by the investigated hybrids. Therefore, the obtained results suggest that both p53-dependent and p53-independent pathways may participate in the biological activity of the AZT-containing derivatives.

Taken together, these findings indicate that appropriately designed phenothiazine-triazole hybrids may represent a valuable platform for the further development of novel multifunctional anticancer compounds. It seems particularly important to exploit the AZT moiety not only as a structural element enabling the application of click chemistry, but also as a biologically active component capable of affecting processes associated with cancer cell proliferation and genome stability. Among the synthesized compounds, particular attention should be paid to compounds **A2**, **A9**, and **A10**.

Compound **A2**, a phenothiazine derivative lacking the AZT moiety, exhibited an interesting biological profile despite its moderate cytotoxic activity compared to derivatives **A9–A12**. In HCT116 cells, relatively low IC_50_ values (50.75 ± 1.67 µM) were observed after 24 h incubation, accompanied by limited toxicity toward BEAS-2B cells (IC_50_ > 100 µM), suggesting partial selectivity of action. In some analyses, precise determination of the SI value was not possible due to IC_50_ values exceeding 100 µM for non-cancerous cells; however, these observations themselves may indicate a low impact of the compound on normal cells. Gene expression analysis in the HCT116 line revealed significant increases in *BCL2*, *MDM2*, *AIFM2*, and *OGG1* expression, together with a particularly pronounced increase in *IL6* and *IL8*, which may indicate activation of stress-related and pro-inflammatory responses. It is noteworthy that after 72 h, IC_50_ values in both cancer cell lines became undetectable, which may suggest effective compensatory activation of pro-survival pathways during prolonged exposure. In the BEAS-2B line, pro-inflammatory cytokines were reduced, similarly to *BCL2* and *OGG1*, whereas *AIFM2* and *MDM2* expression levels were elevated. Apoptosis analysis after 24 h demonstrated marked induction of early apoptosis in HCT116 cells, with a considerably weaker effect observed in BEAS-2B cells. At the same time, **A2** induced moderate disturbances in cell cycle progression in BEAS-2B cells and a visible increase in the sub-G1 phase in HCT116 cells, accompanied only by a slight increase in ROS levels, suggesting that its activity was not primarily associated with enhanced oxidative stress. Overall, the obtained results suggest that the modified phenothiazine core itself possesses the ability to induce controlled apoptotic cell death with relatively limited toxicity toward non-cancerous cells.

Particularly interesting results were obtained for compound **A10**, which represents an **A2** derivative conjugated with the AZT moiety. Introduction of AZT markedly enhanced the biological activity of the compound, leading to stronger cytotoxic effects against all cell lines, particularly after 72 h, as well as more pronounced apoptosis induction than that observed for **A2**. Apoptosis analysis demonstrated predominance of the population of cells undergoing early apoptosis in both HCT116 and BEAS-2B cells, indicating substantially stronger biological activity of this derivative. Compound **A10** reduced the expression of *AIFM2* and *OGG1*, which may have sensitized cells to oxidative stress. Additionally, it reduced *MDM2* expression levels, which may decrease its interaction with p53 and facilitate apoptosis induction. At the same time, decreased expression of both pro-inflammatory cytokine genes in HCT116 cells and *IL8* in BEAS-2B cells was observed. Compound **A10** also caused marked disturbances in cell cycle progression associated with accumulation of cells in the S and G2/M phases in HCT116 cells and in the sub-G1 phase in BEAS-2B cells. However, after prolonged incubation, a decrease in SI values below 1 was observed, which was associated with increased toxicity toward BEAS-2B cells. The activity of **A10** was accompanied by a moderate increase in ROS levels, suggesting that its cytotoxic mechanism may be primarily associated with enhancement of replicative stress, activation of the DNA damage response, and induction of apoptotic pathways related to the presence of the AZT moiety. Thus, direct comparison of **A2** and **A10** clearly indicates that conjugation with AZT may significantly enhance the cytotoxic activity of phenothiazine derivatives, although partially at the expense of their biological selectivity.

Among all analyzed derivatives, compound **A9** exhibited the most favorable and well-balanced biological profile. This derivative demonstrated very good cytotoxic activity against HCT116 and HT-29 cells while maintaining favorable SI values after both 24 h and 72 h incubation. Apoptosis analysis revealed an almost complete shift in the HCT116 cell population into early apoptosis (over 96%), whereas in BEAS-2B cells a relatively high proportion of viable cells, approximately 50%, was still maintained, indicating a more selective anticancer effect than that observed for **A10**. Compound **A9** also caused a marked increase in the sub-G1 population in BEAS-2B cells and disturbances in cell cycle progression associated with accumulation of cells in the S and G2/M phases in HCT116 cells, confirming its strong pro-apoptotic and cytostatic activity. Importantly, despite its high biological activity, only a slight increase in ROS levels was observed in both HCT116 and BEAS-2B cells, suggesting a more controlled mechanism of action not based solely on nonspecific induction of oxidative stress.

Additionally, **A9** reduced the expression of genes associated with cell survival and oxidative stress response, such as *MDM2*, *AIFM2*, and *OGG1*, in BEAS-2B cells, while simultaneously inducing compensatory increases in the expression of these genes in HCT116 cells, together with increased *BCL2* expression in both cell lines. Despite this defensive response, the majority of HCT116 cells still underwent early apoptosis; therefore, this increase is interpreted as a biologically ineffective protective response of the cells against the cytostatic activity of the compound. Despite activation of adaptive pathways, the extent of cellular damage appears sufficient to trigger programmed cell death, as confirmed by our experimental results. As described in the literature, the signal initiating cell death remains suppressed until survival signals are abolished, as occurs during rapid degradation of BCL2 proteins in cells exposed to stress factors [104]. However, it should also be taken into account that cells surviving treatment may undergo adaptation, and increased expression of pro-survival proteins may contribute to therapy resistance [104]. Overall, these findings demonstrate the ability of compound **A9** to disrupt cancer cell homeostasis, while its final biological effect may depend on the balance between anti- and pro-apoptotic responses within the organism, which should be further investigated in vivo.

It should be noted that the mechanistic assays were performed using a single concentration of 100 µM to enable direct comparison of all analyzed compounds under standardized experimental conditions and to identify early cellular responses within a relatively short incubation period. This concentration was selected as an in vitro screening condition rather than as a direct reflection of clinically achievable AZT exposure. Therefore, the obtained results should be interpreted as a preliminary mechanistic characterization of the synthesized hybrids. Further studies performed at lower concentrations and over extended exposure times may provide additional insight into the concentration dependence and therapeutic relevance of the observed effects.

In summary:

- Molecular hybridization of phenothiazine derivatives with the AZT moiety led to enhanced anticancer activity of the investigated compounds.
- Compounds containing the AZT fragment (**A9–A12**) exhibited stronger cytotoxic activity than derivatives lacking azidothymidine.
- The obtained results indicate the presence of a cytostatic effect accompanied by activation of programmed cell death.
- Clear differences were observed between the responses of cancerous and non-cancerous cells, indicating partial selectivity of action of the analyzed derivatives.
- Most of the investigated compounds induced predominantly early apoptosis with a relatively low contribution of necrosis.
- The analyzed compounds represent an interesting group of candidates for further development as potential multitarget anticancer agents directed against colorectal cancer.

The obtained results indicate that molecular hybridization involving the AZT moiety may effectively enhance the biological activity of phenothiazine derivatives; however, the final activity profile also depends on the nature of the substituents present within the compound structure. Comparison of **A2** and **A10** directly demonstrates the impact of the AZT fragment on increasing anticancer activity, whereas the profile of **A9** suggests that an appropriate combination of the phenothiazine scaffold, triazole moiety, and AZT may lead to the development of more balanced and selective candidates for further anticancer studies.

## Figures and Tables

**Table 1:** IC_50_ values of compounds **A1–A12** determined in BEAS-2B, HCT116, and HT-29 cell lines after 24 h and 72 h incubation using the Alamar Blue assay. Results are expressed in µM as mean ± SD.

	HCT116 24 h	HCT116 72 h	HT-29 24 h	HT-29 72 h	BEAS-2B 24 h	BEAS-2B 72 h
**A1**	110.0 ± 3.2	IC_50_ > 100	177.9 ± 12.9	IC_50_ > 100	IC_50_ > 100	IC_50_ > 100
**A2**	50.75 ± 1.67	IC_50_ > 100	94.8 ± 1.0	IC_50_ > 100	IC_50_ > 100	IC_50_ > 100
**A3**	155.8 ± 2.4	IC_50_ > 100	195.1 ± 3.9	IC_50_ > 100	IC_50_ > 100	IC_50_ > 100
**A4**	175.3 ± 13.5	IC_50_ > 100	IC_50_ > 100	IC_50_ > 100	IC_50_ > 100	IC_50_ > 100
**A5**	188.7 ± 5.8	113.2 ± 1.8	80.5 ± 3.0	72.5 ± 4.2	IC_50_ > 100	49.3 ± 0.5
**A6**	162.1 ± 13.6	IC_50_ > 100	113.7 ± 1.4	IC_50_ > 100	IC_50_ > 100	IC_50_ > 100
**A7**	111.3 ± 3.4	IC_50_ > 100	102.9 ± 0.4	IC_50_ > 100	IC_50_ > 100	IC_50_ > 100
**A8**	65.0 ± 5.1	74.0 ± 0.4	78.0 ± 1.4	80.7 ± 2.9	111.8 ± 0.5	66.9 ± 1.1
**A9**	42.0 ± 0.5	45.0 ± 0.6	46.2 ± 0.9	49.3 ± 1.2	72.2 ± 0.5	61.0 ± 1.0
**A10**	64.5 ± 2.2	72.0 ± 1.0	78.1 ± 1.9	80.5 ± 3.3	113.7 ± 9.1	42.6 ± 0.1
**A11**	69.6 ± 1.5	46.7 ± 0.2	75.1 ± 1.8	56.0 ± 1.4	86.2 ± 0.1	37.7 ± 0.1
**A12**	37.0 ± 1.3	46.8 ± 0.5	67.9 ± 1.4	65.6 ± 2.0	104.5 ± 8.0	44.6 ± 1.2

**Table 2:** Selectivity index (SI) values calculated for AZT-containing compounds **A9–A12** in HCT116 and HT-29 colorectal cancer cell lines after 24 h and 72 h incubation. SI values were determined as the ratio of IC_50_ obtained for BEAS-2B cells to the corresponding IC_50_ value for cancer cells. SI > 1 indicates preferential cytotoxicity toward cancer cells.

Compound	SI HCT116 24 h	SI HT-29 24 h	SI HCT116 72 h	SI HT-29 72 h
**A9**	1.72	1.56	1.36	1.24
**A10**	1.76	1.46	0.59	0.53
**A11**	1.24	1.15	0.81	0.67
**A12**	2.82	1.54	0.95	0.68

**Table 3:** Selected physicochemical, ADME, and drug-likeness parameters of compounds **A2** and **A9–A12** predicted using the SwissADME platform.

Compound	−X	MW (g/mol)	TPSA (Å^2^)	LogP	LogS (ESOL)	Solubility Class	GI Absorption	Lipinski Violations	Bioavailability Score
**A2**	F	268.31	50.22	4.30	−4.84	Moderately soluble	High	1 (MLOGP > 4.15)	0.55
**A9**	H	557.62	151.01	1.76	−4.95	Moderately soluble	Low	2 (MW > 500, NorO > 10)	0.17
**A10**	F	575.61	151.01	2.12	−5.11	Moderately soluble	Low	2 (MW > 500, NorO > 10)	0.17
**A11**	Cl	592.07	151.01	2.35	−5.55	Poorly soluble	Low	2 (MW > 500, NorO > 10)	0.17
**A12**	SCH_3_	603.72	176.31	2.34	−5.47	Poorly soluble	Low	2 (MW > 500, NorO > 10)	0.17

**Table 4:** Primer sequences.

Target Gene	Primer Sequence
*BCL2*	F: CTTCAGGGACGGGGTGAAC R: GGATCCAGGTGTGCAGGTG
*MDM2*	F: GCCCTTCGTGAGAATTGGCT R: CCTCAACACATGACTCTCTGG
*AIFM2*	F: CTGCCCTTCTCTCATCTTATCCT R: CTGCCTCACCATGTCCTCATAG
*IL6*	F: GTTCTGCCAAACCAGCCTTG R: AGATCACCTAGTCCACCCCC
*IL8*	F: GGTGCAGTTTTGCCAAGGAG R: ACCAAGGCACAGTGGAACAA
*RPL41*	F: TCCTGCGTTGGGATTCCGTG R: ACGGTGCAACAAGCTAGCGG
*OGG1*	F: CCTGTGGGGACCTTATGCTG R: TGTGAATCCCCTCTCCCGAT

## Data Availability

The original contributions presented in this study are included in the article/Supplementary Material. Further inquiries can be directed to the corresponding authors.

## Supplementary Materials

The following supporting information can be downloaded at: https://www.mdpi.com/article/10.3390/ijms27125562/s1.

## Abbreviations

The following abbreviations are used in this manuscript:

AIDS	Acquired Immunodeficiency Syndrome
*AIFM2*	Apoptosis-Inducing Factor Mitochondria-Associated 2
ALT	Alternative Lengthening of Telomeres
Annexin V	Annexin V apoptosis detection assay
AZT	3′-Azido-3′-deoxythymidine (Zidovudine)
AZT-TP	Zidovudine triphosphate
Bax	*BCL2*-associated X protein
BEAS-2B	Human bronchial epithelial cell line
BER	Base excision repair
*BCL2*	B-cell lymphoma 2
Chk1	Checkpoint kinase 1
Chk2	Checkpoint kinase 2
CuAAC	Copper-catalyzed azide-alkyne cycloaddition
DMEM	Dulbecco’s Modified Eagle Medium
EBV	Epstein–Barr virus
FDA	Food and Drug Administration
FBS	Fetal bovine serum
HCT116	Human colorectal carcinoma cell line
HepG2	Human hepatocellular carcinoma cell line
HIV-1	Human Immunodeficiency Virus type 1
HRMS	High-resolution mass spectrometry
HT-29	Human colorectal adenocarcinoma cell line
HTLV-I	Human T-cell leukemia virus type I
IC_50_	Half maximal inhibitory concentration
IFNβ	Interferon beta
*IL6*	Interleukin 6
*IL8*	Interleukin 8
MAPK/ERK	Mitogen-activated protein kinase/extracellular signal-regulated kinase
*MDM2*	Mouse Double Minute 2 homolog
*MDMX*	Mouse Double Minute X
NMR	Nuclear magnetic resonance
*OGG1*	8-Oxoguanine DNA Glycosylase 1
PBS	Phosphate-buffered saline
PI	Propidium iodide
Puma	p53 upregulated modulator of apoptosis
ROS	Reactive oxygen species
*RPL41*	Ribosomal protein L41
RPMI	Roswell Park Memorial Institute medium
RT-qPCR	Reverse transcription quantitative polymerase chain reaction
SAR	Structure-activity relationship
