# Supplementary material for: Quinobenzothiazine–AZT Hybrids Linked via 1,2,3-Triazole: Rational Design, Synthesis, and Biological Evaluation as Anticancer Agents
Source: Int J Mol Sci. 2026 Jun 19;27(12):5562. doi: 10.3390/ijms27125562 (PMC13299229; doi:10.3390/ijms27125562)
Supplement: Supplementary file 1 [file ijms-27-05562-s001.zip › ijms-4377605-supplementary.pdf]

Quinobenzothiazine–AZT Hybrids Linked via 1,2,3-Triazole: Rational Design, Synthesis, and Biological Evaluation as Anticancer Agents

Klaudia Giercuszkiewicz-Haśnik[1,2,3,*], Magdalena Skonieczna[1,2], Beata Morak-Młodawska[4] and Małgorzata Jeleń[4,*]

[1] Department of Systems Biology and Engineering, The Silesian University of Technology, Akademicka Street 16, 44-100 Gliwice, Poland; klaudia.giercuszkiewicz@polsl.pl (K.G.-H.); magdalena.skonieczna@polsl.pl (M.S.)
[2] Centre of Biotechnology, Silesian University of Technology, Krzywoustego Street 8, 44-100 Gliwice, Poland
[3] Faculty of Medical Sciences in Katowice, Medical University of Silesia, 40-752 Katowice, Poland
[4] Department of Organic Chemistry, Faculty of Pharmaceutical Sciences in Sosnowiec, Medical University of Silesia in Katowice, Jagiellońska Street 4, 41-200 Sosnowiec, Poland; manowak@sum.edu.pl (M.J.), bmlodawska@sum.edu.pl (B.M.-M.)
* Correspondence: klaudia.giercuszkiewicz@polsl.pl (K.G.-H.), manowak@sum.edu.pl (M.J.)

Content:

^{1}H NMR and ^{13}C NMR spectra and HR MS of compounds **A9–A12**.

Figure S1. Representative cell viability curves showing the concentration-dependent effects of the analyzed compounds on HCT116, HT-29, and BEAS-2B cells after 24 and 72 h of treatment.

Figure S2. Representative microscopic images of HCT116 cells after 24 h treatment with compounds **A1–A12** at a concentration of 100 µM under standard culture conditions. Images were acquired at 10× magnification.

Figure S3. Representative microscopic images of HT29 cells after 24 h treatment with compounds **A1–A12** at a concentration of 100 µM under standard culture conditions. Images were acquired at 10× magnification.

Figure S4. Representative microscopic images of BEAS-2B cells after 24 h treatment with compounds **A1–A12** at a concentration of 100 µM under standard culture conditions. Images were acquired at 10× magnification.

Figure S5. Representative flow cytometry histograms showing intracellular ROS levels in HCT116 and BEAS-2B cells following treatment with compounds **A1–A12** for 24 h.

Figure S6. Quantitative cell cycle distribution of HCT116 cells after 24 h treatment with compounds **A1–A12** at a concentration of 100 µM.

Figure S7. Quantitative cell cycle distribution of BEAS-2B cells after 24 h treatment with compounds **A1–A12** at a concentration of 100 µM.

Figure S8. Representative Annexin V-FITC/PI dot plots of HCT116 and BEAS-2B cells treated with compounds **A1–A12** for 24 h.

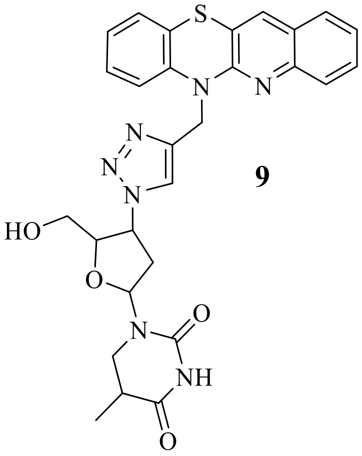

9

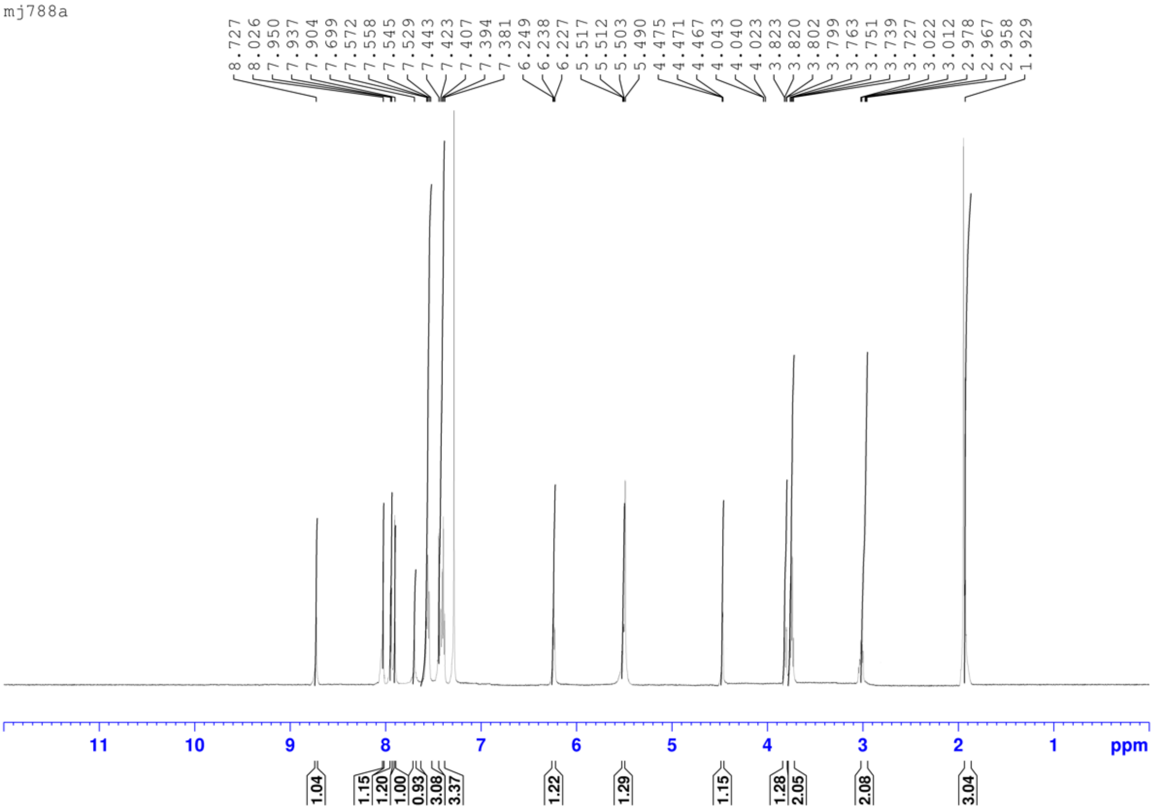
mj788a
8.727
8.026
7.950
7.937
7.904
7.699
7.572
7.558
7.545
7.529
7.443
7.423
7.407
7.394
7.381
6.249
6.238
6.227
5.517
5.512
5.503
5.490
4.475
4.471
4.467
4.043
4.040
4.023
3.823
3.820
3.802
3.799
3.763
3.751
3.739
3.727
3.022
3.012
2.978
2.967
2.958
1.929
11
10
9
8
7
6
5
4
3
2
1
ppm
1.04
1.15
1.20
1.00
0.93
3.08
3.37
1.22
1.29
1.15
1.28
2.05
2.08
3.04

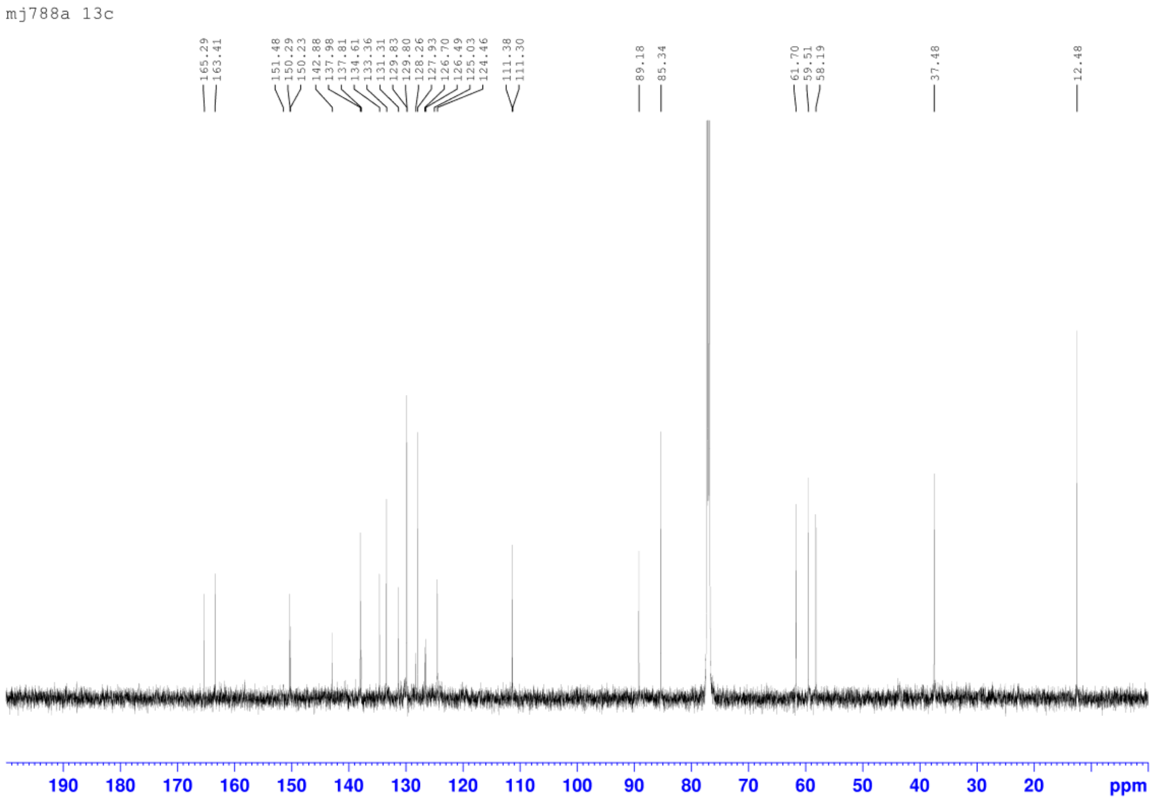

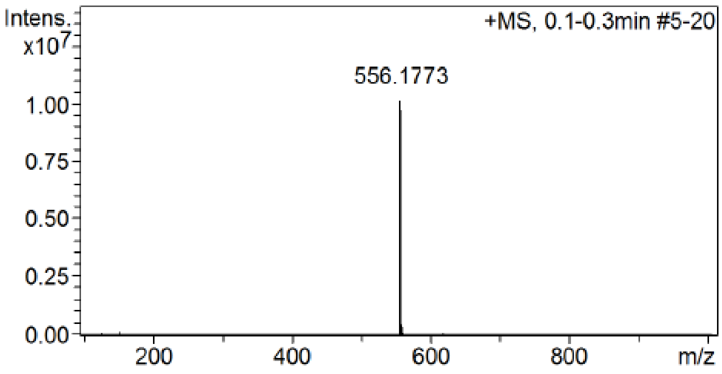

#	m/z	Res.	S/N	I	I %	FWHM
1	556.1773	18771	14238.5	1606245	100.0	0.0296

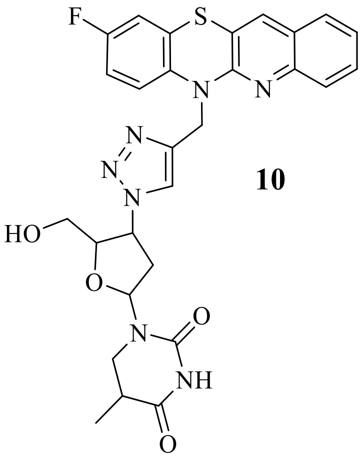

10

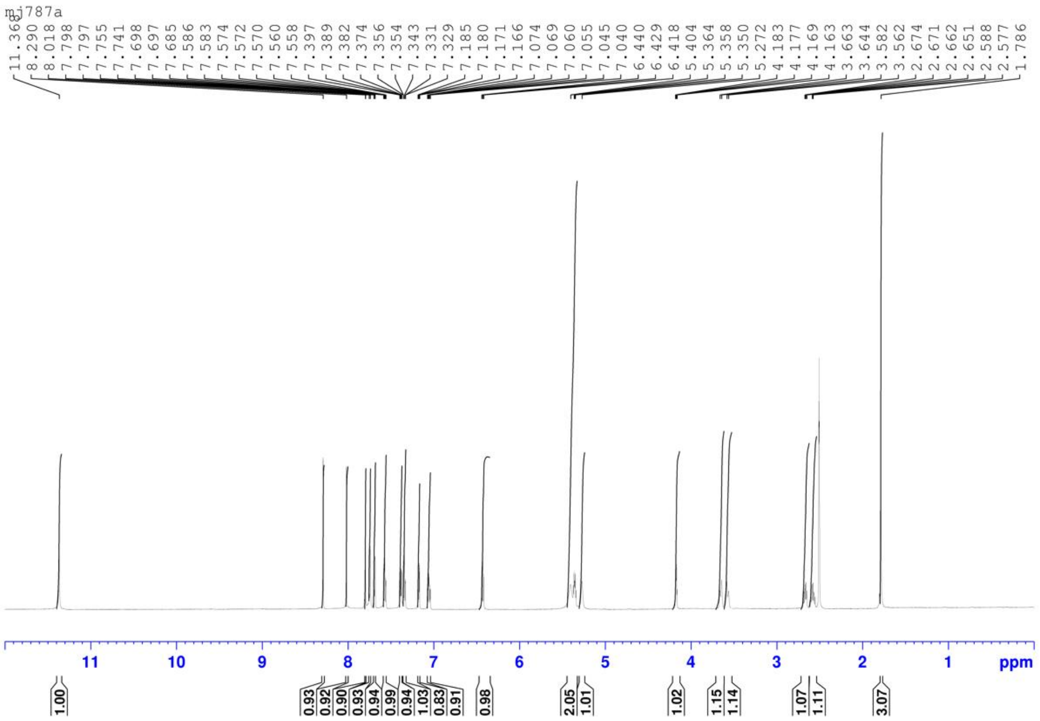

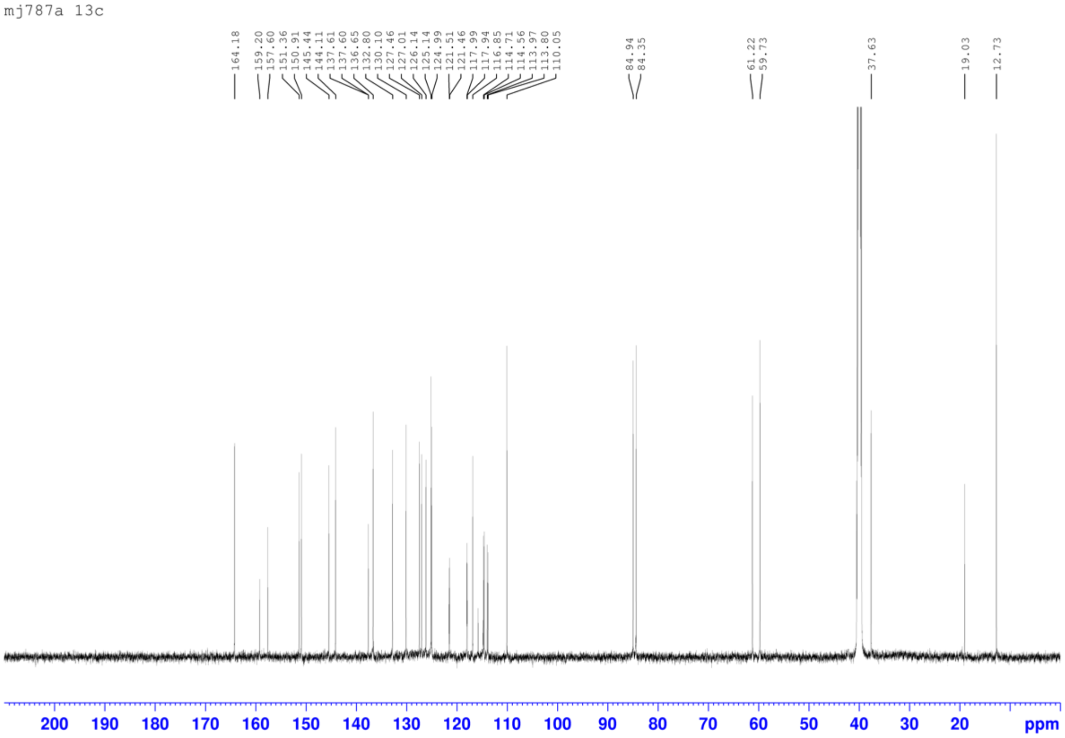

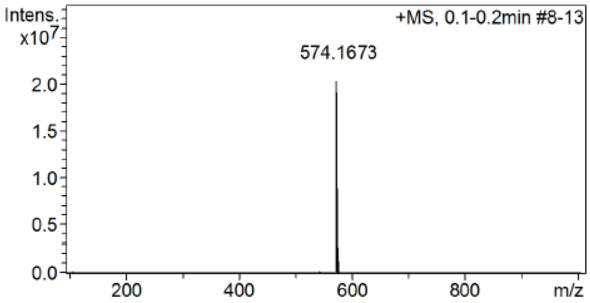

#	m/z	Res.	S/N	I	I %	FWHM
1	574.1673	28906	4776.0	978795	100.0	0.0199

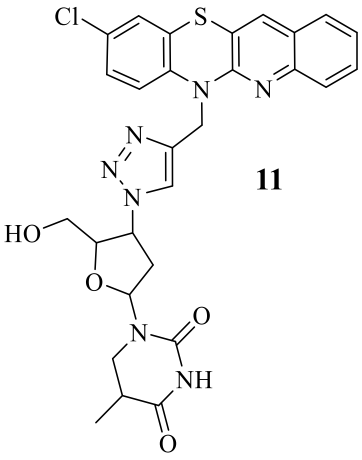
Cl
S
N
N
N
N
N
HO
O
O
NH
O

11

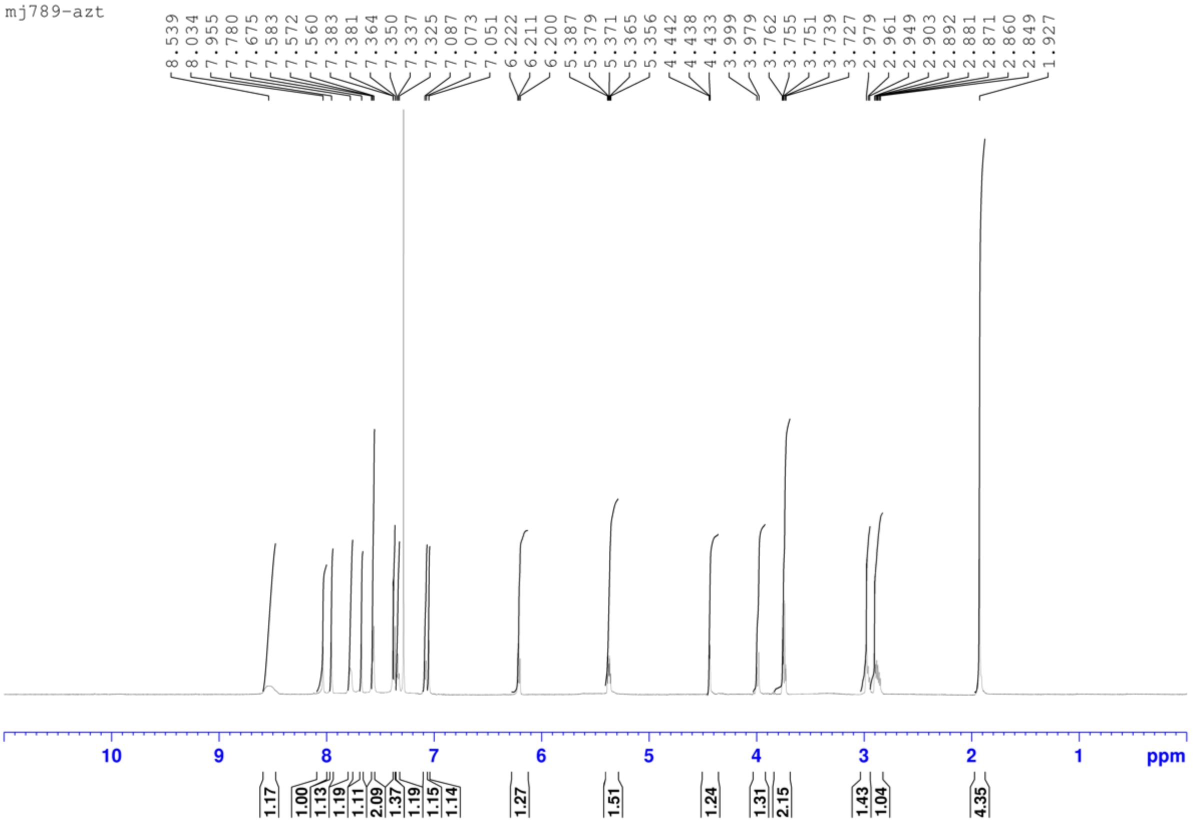
mj789-azt
8.539
8.034
7.955
7.780
7.675
7.583
7.572
7.560
7.383
7.381
7.364
7.350
7.337
7.325
7.087
7.073
7.051
6.222
6.211
6.200
5.387
5.379
5.371
5.365
5.356
4.442
4.438
4.433
3.999
3.979
3.762
3.755
3.751
3.739
3.727
2.979
2.961
2.949
2.903
2.892
2.881
2.871
2.860
2.849
1.927
10
9
8
7
6
5
4
3
2
1
ppm
1.17
1.00
1.13
1.19
1.11
2.09
1.37
1.19
1.15
1.14
1.27
1.51
1.24
1.31
2.15
1.43
1.04
4.35

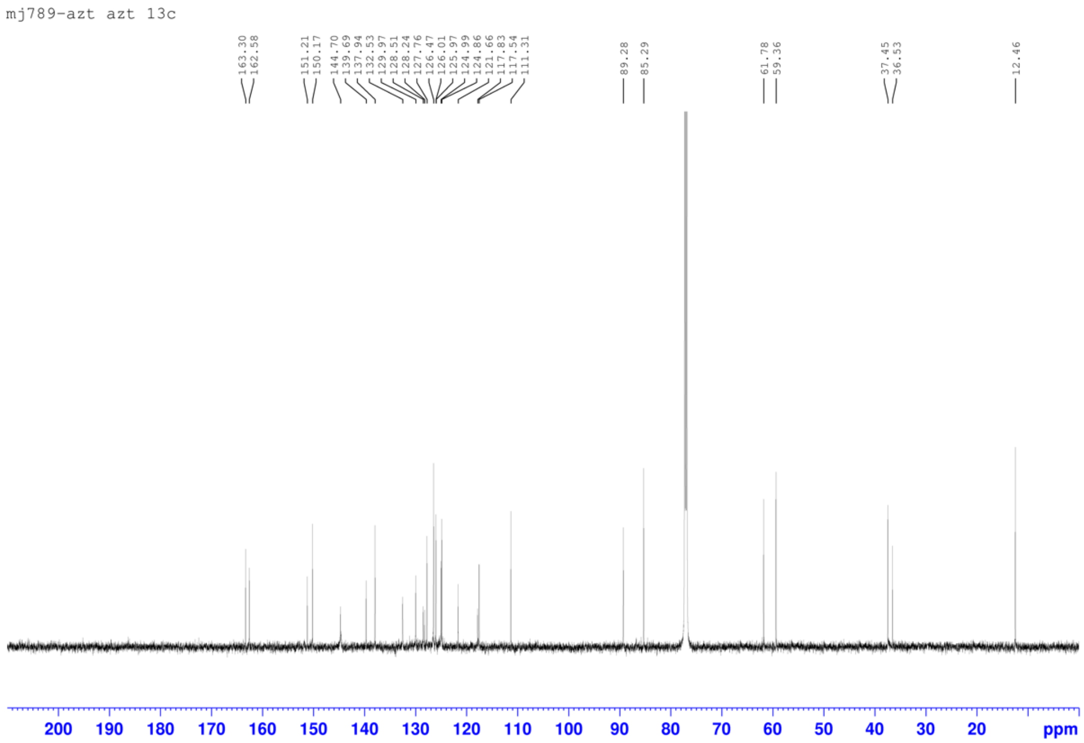

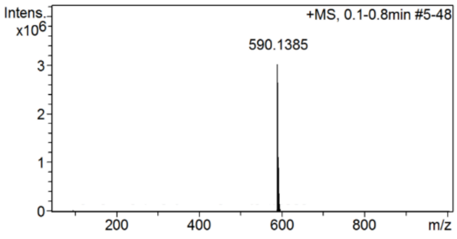

#	m/z	Res.	S/N	I	I %	FWHM
1	590.1385	20570	2621.5	87113	100.0	0.0287

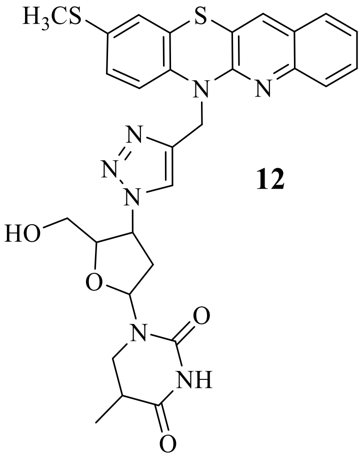

12

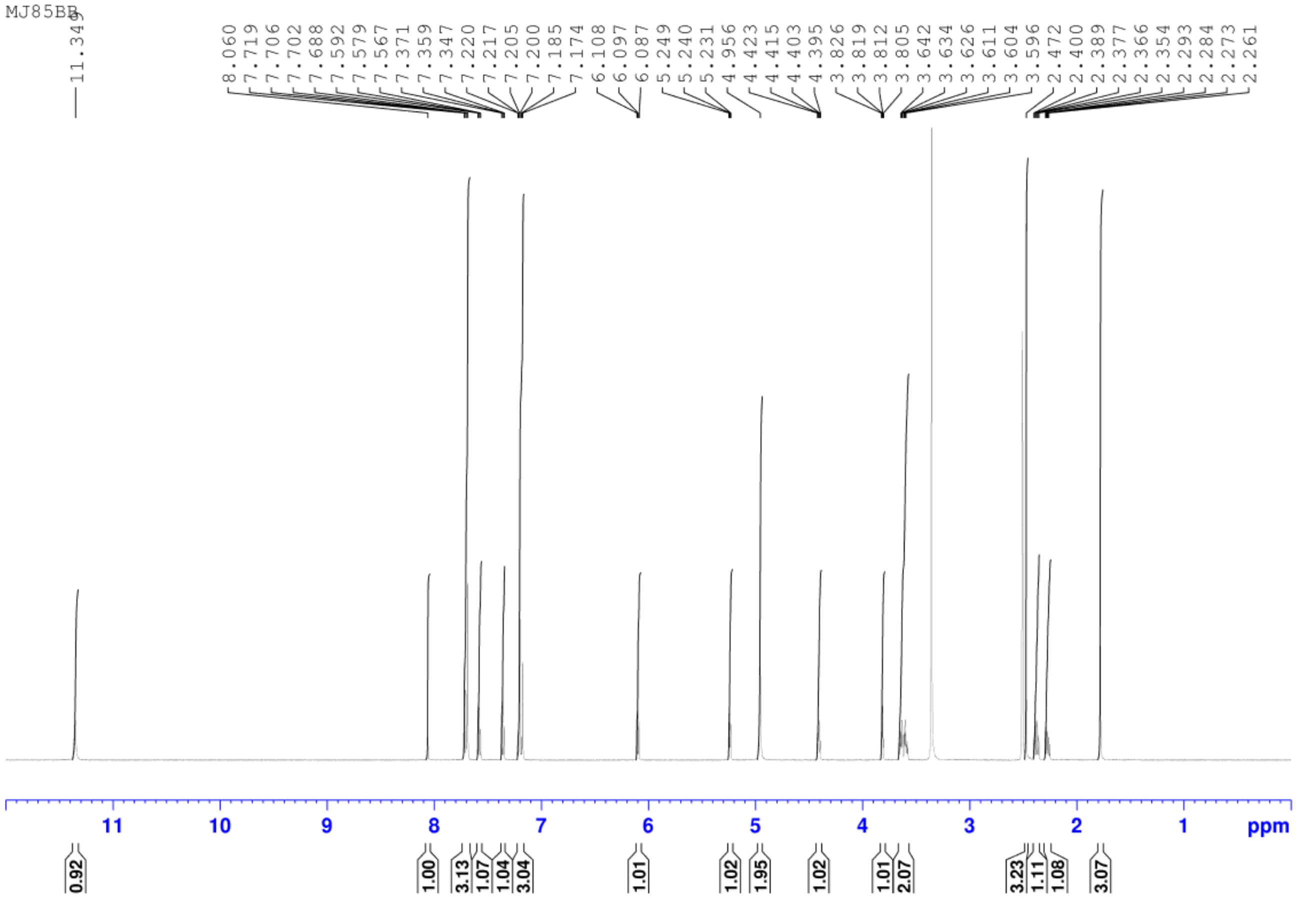
MJ85BB
11.349
8.060
7.719
7.706
7.702
7.688
7.592
7.579
7.567
7.371
7.359
7.347
7.220
7.217
7.205
7.200
7.185
7.174
6.108
6.097
6.087
5.249
5.240
5.231
4.956
4.423
4.415
4.403
4.395
3.826
3.819
3.812
3.805
3.642
3.634
3.626
3.611
3.604
3.596
2.472
2.400
2.389
2.377
2.366
2.354
2.293
2.284
2.273
2.261
11
10
9
8
7
6
5
4
3
2
1
ppm
0.92
1.00
3.13
1.07
1.04
3.04
1.01
1.02
1.95
1.02
1.01
2.07
3.23
1.11
1.08
3.07

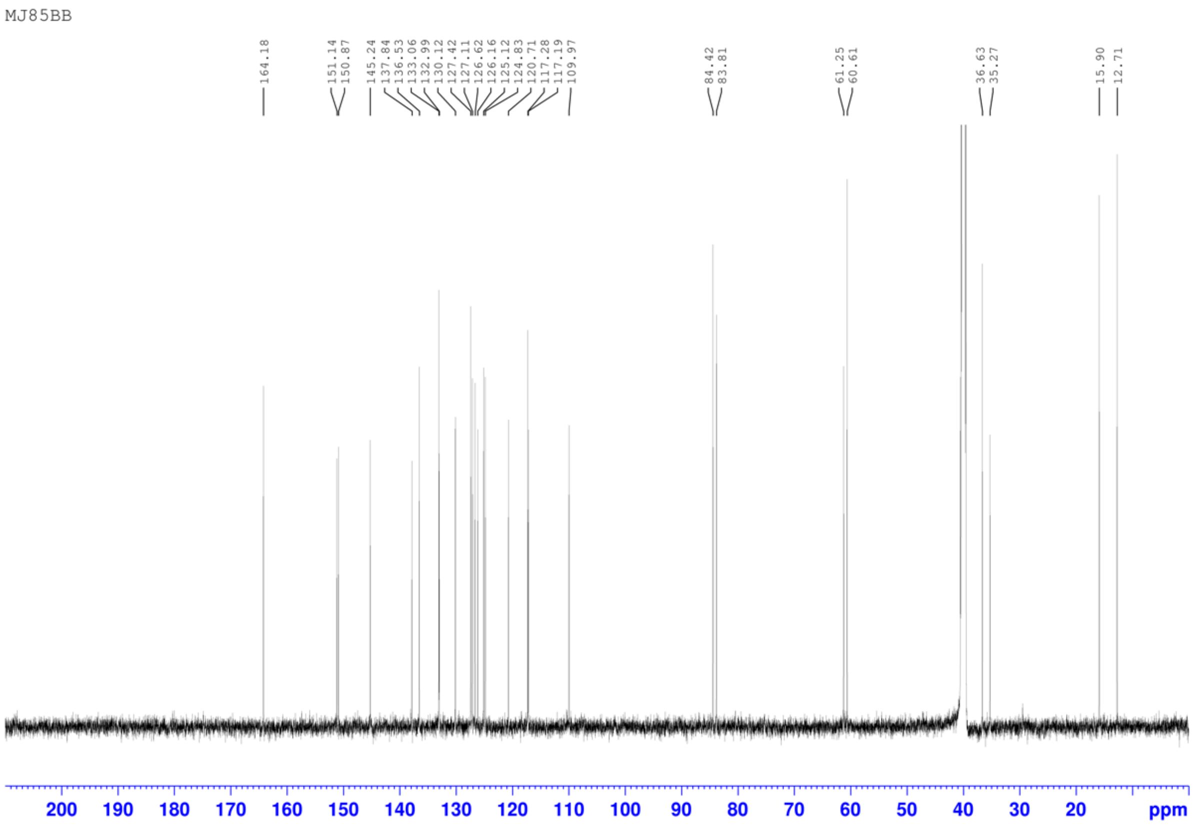

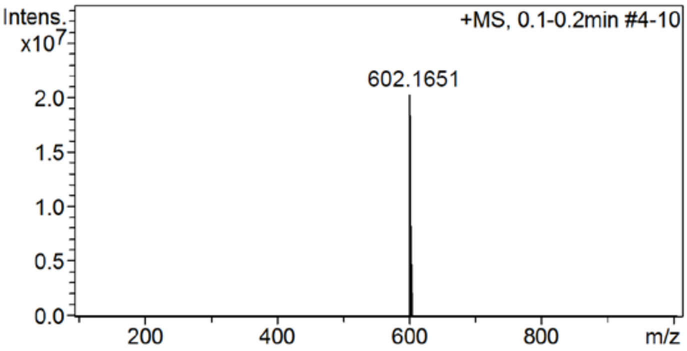

#	m/z	Res.	S/N	I	I %	FWHM
1	602.1651	22856	493.7	216399	100.0	0.0263

Figure S1. Representative cell viability curves showing the concentration-dependent effects of the analyzed compounds on HCT116, HT-29, and BEAS-2B cells after 24 and 72 h of treatment. Cell viability was assessed using the Alamar Blue assay and expressed as a percentage relative to untreated control cells, considered as 100% viability.

HCT116	24h	72h
**A1**	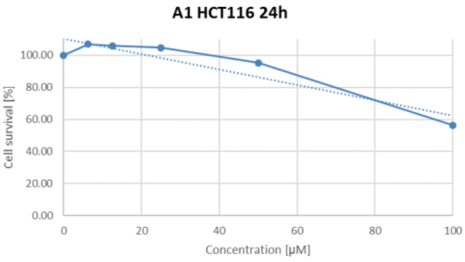	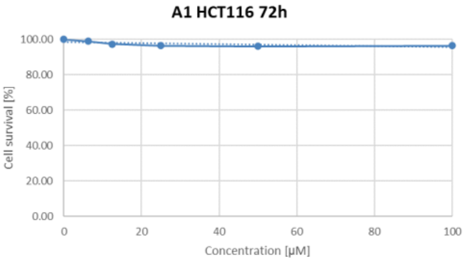
**A2**	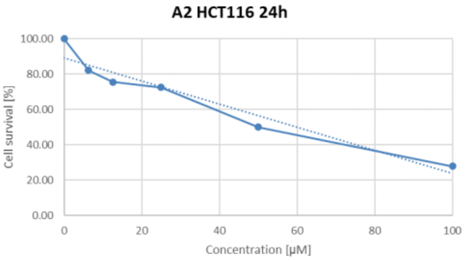	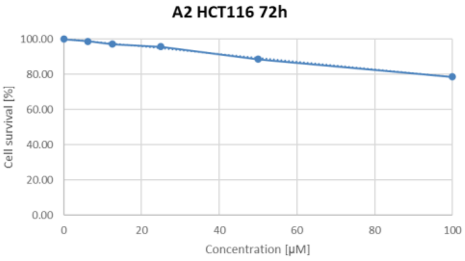
**A3**	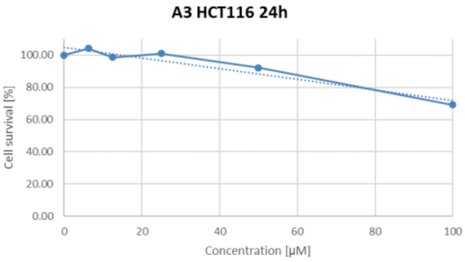	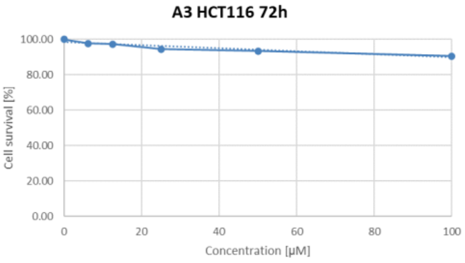
**A4**	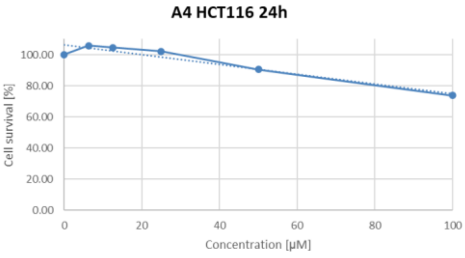	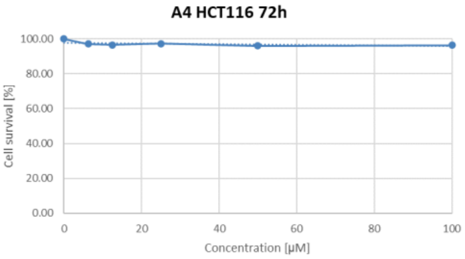
**A5**	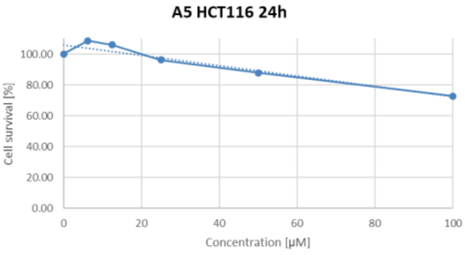	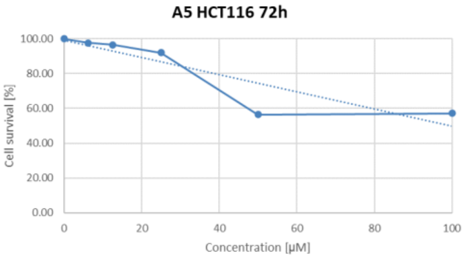
**A6**	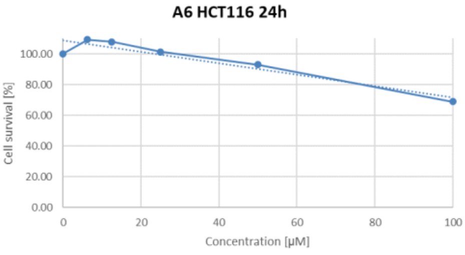	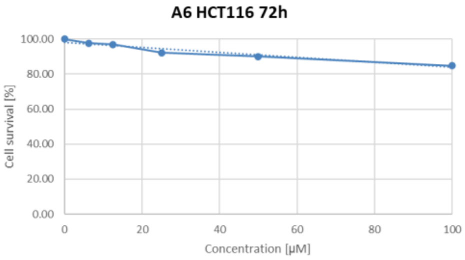

A7

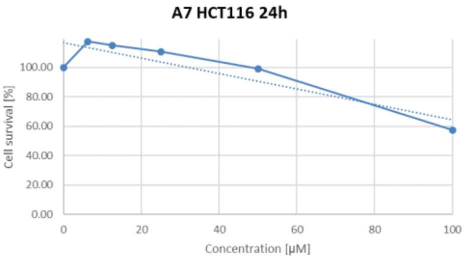

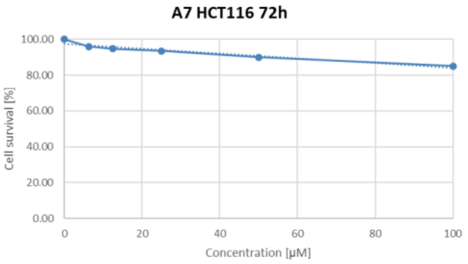

A8

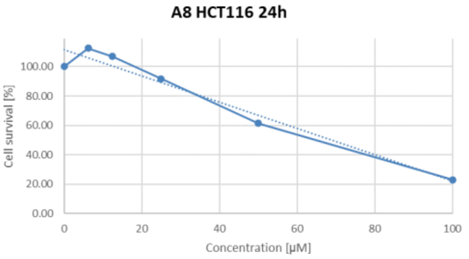

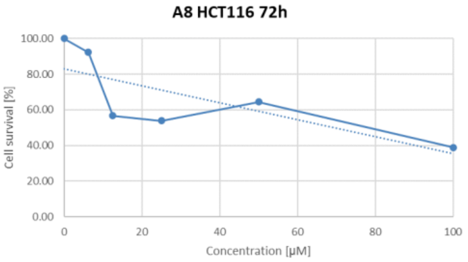

A9

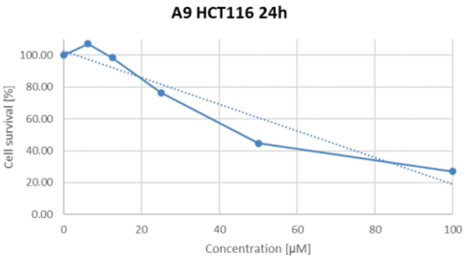

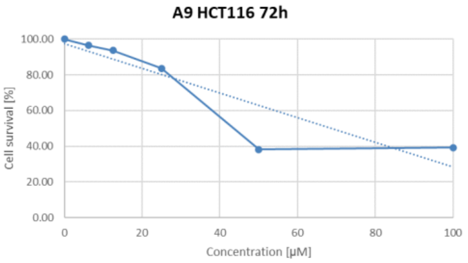

A10

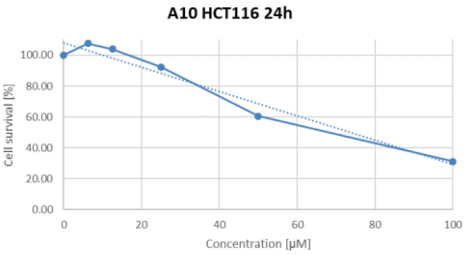

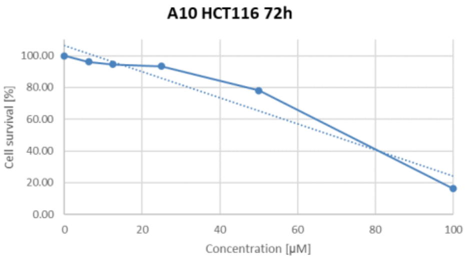

A11

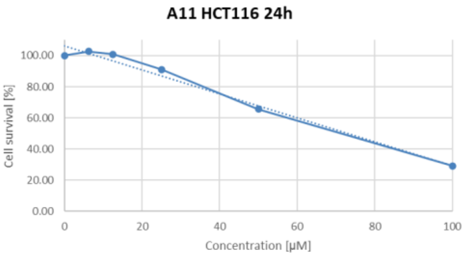

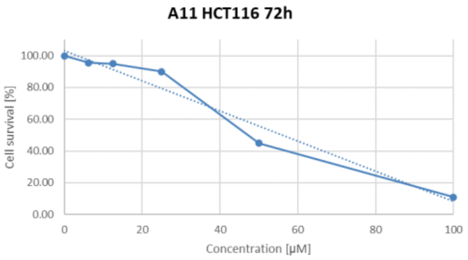

A12

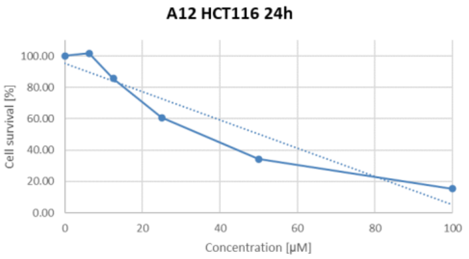

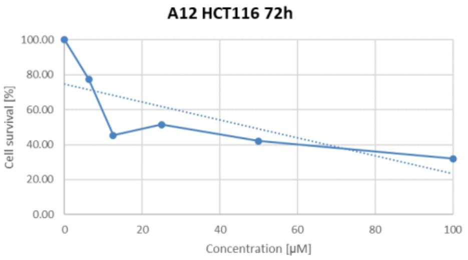

HT29	24h	72h

A1

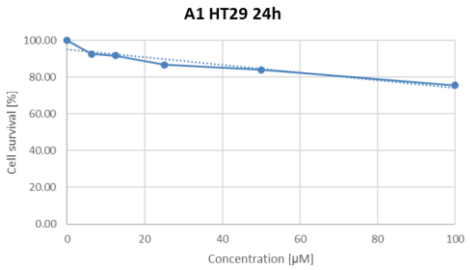

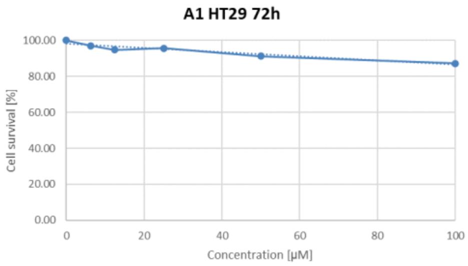

A2

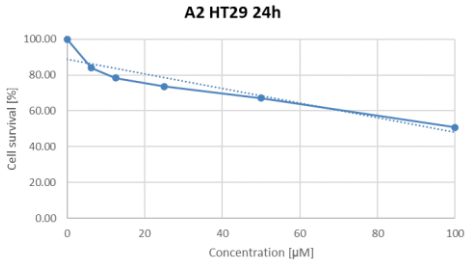

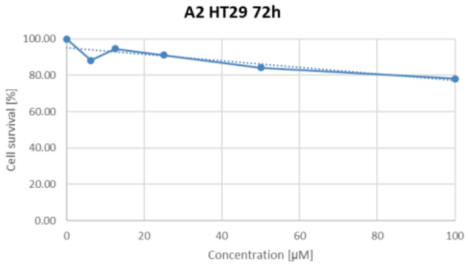

A3

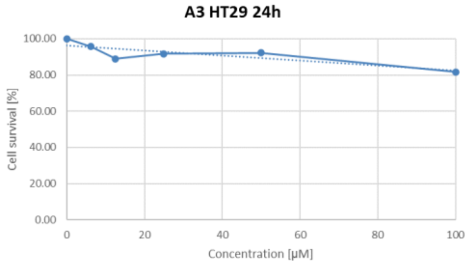

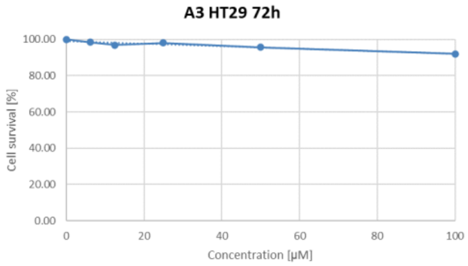

A4

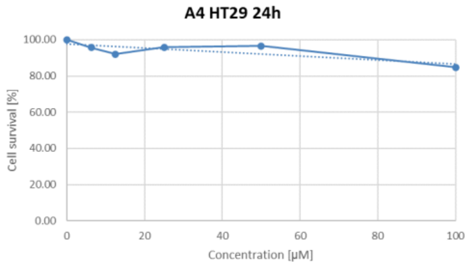

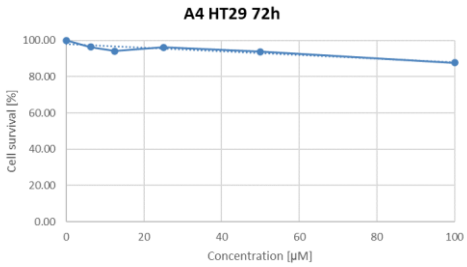

A5

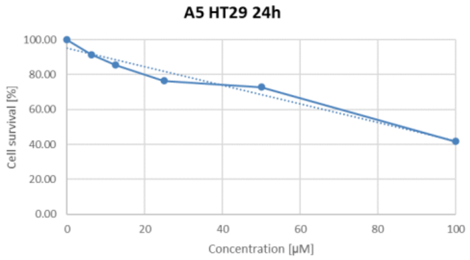

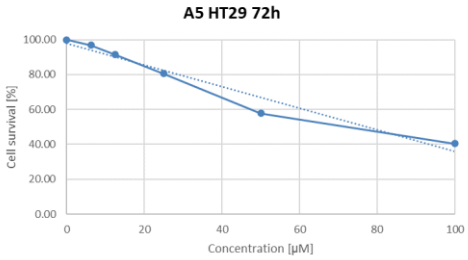

A6

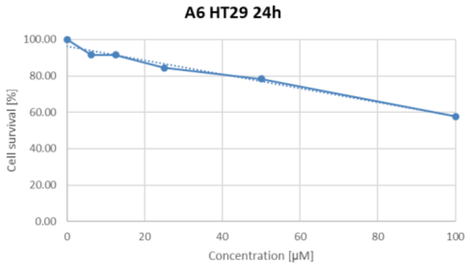

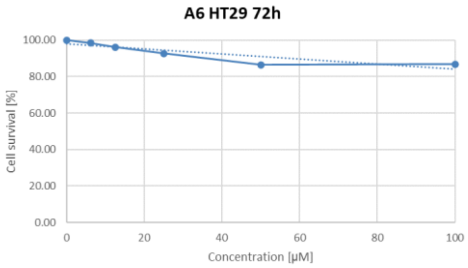

A7

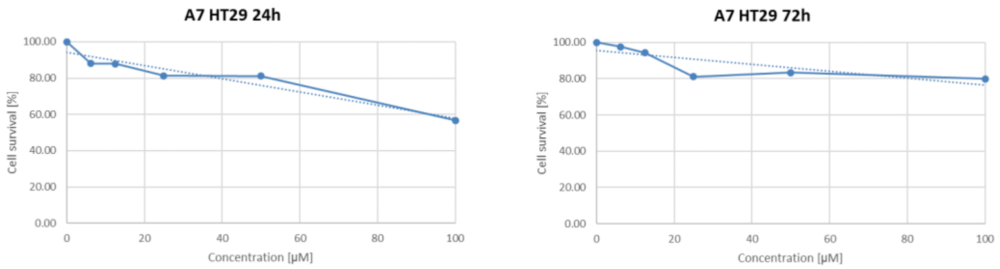

A8

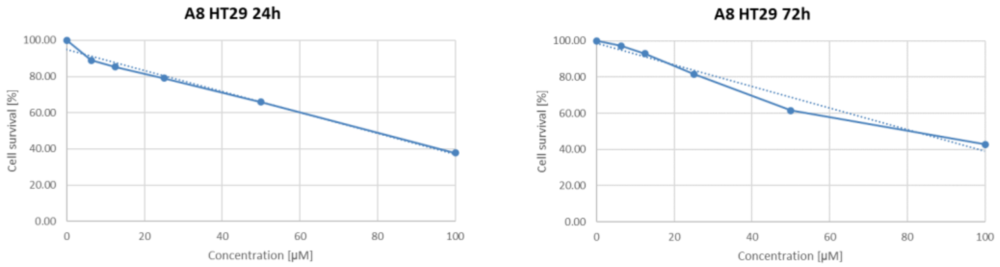

A9

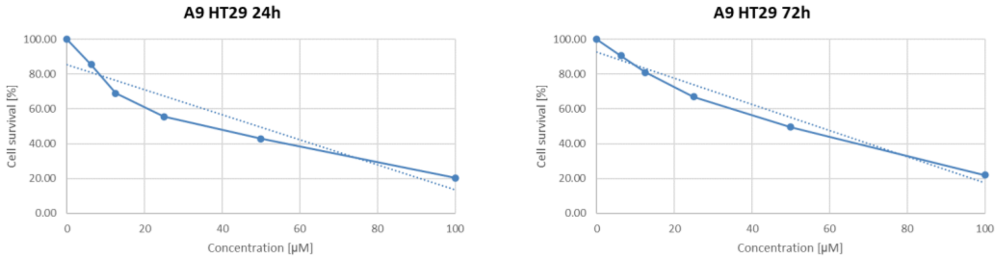

A10

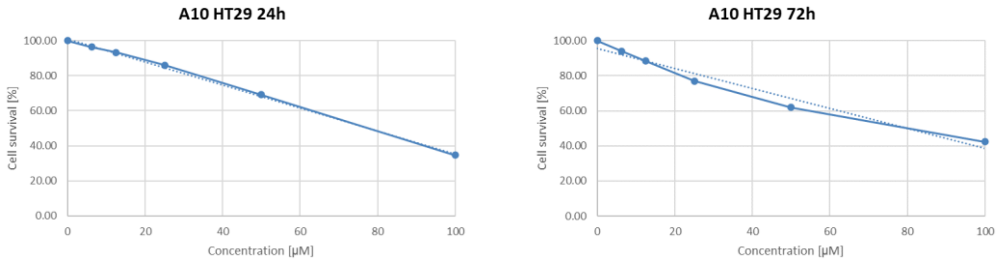

A11

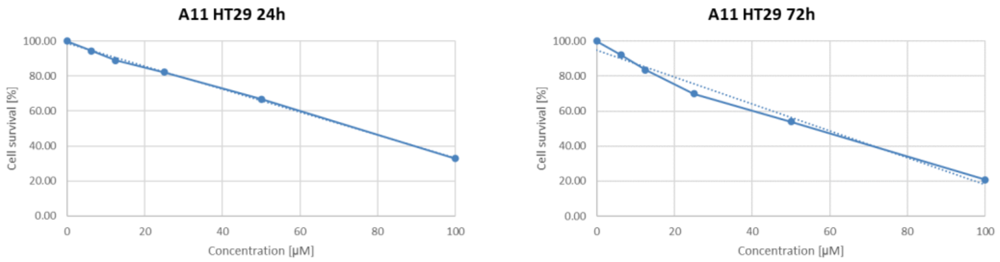

A12

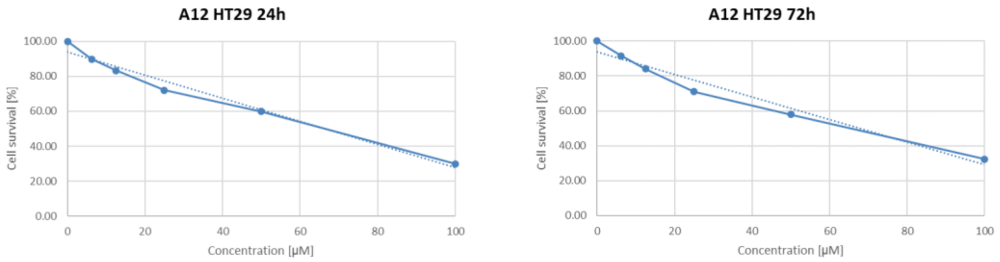

BEAS-2B	24h	72h

A1

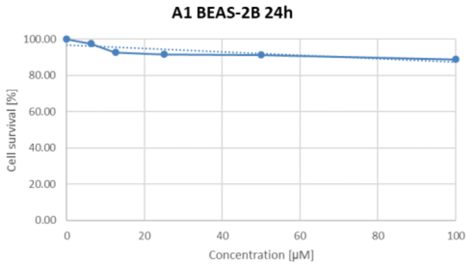

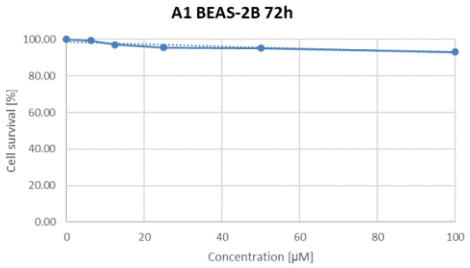

A2

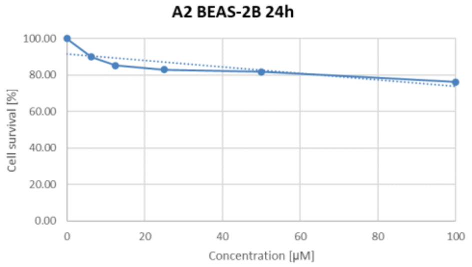

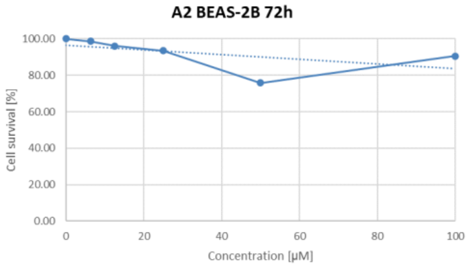

A3

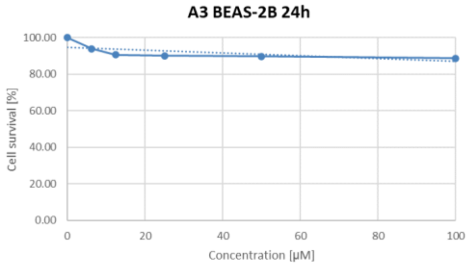

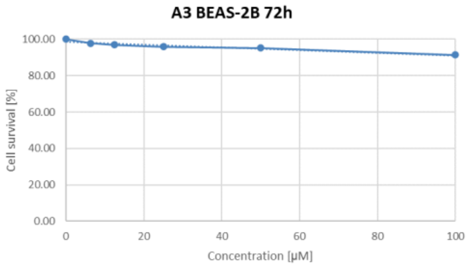

A4

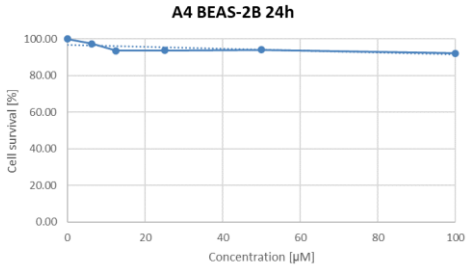

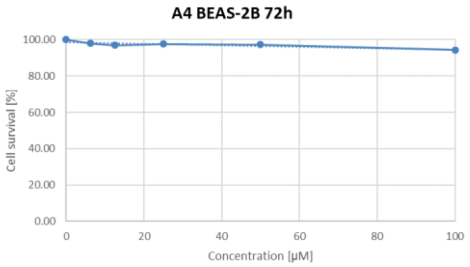

A5

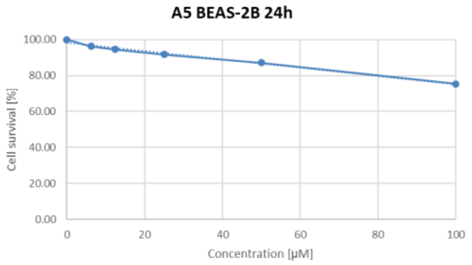

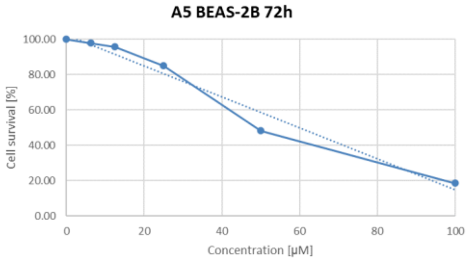

A6

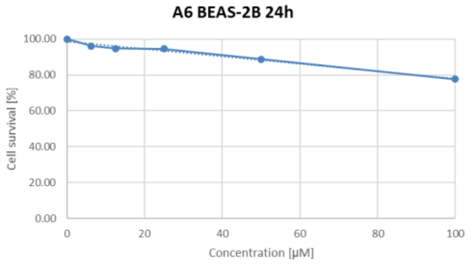

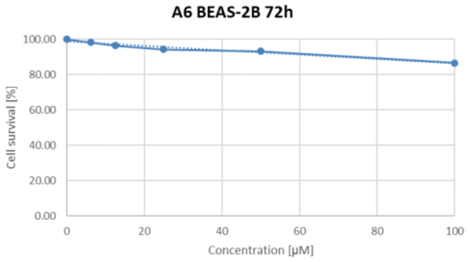

A7

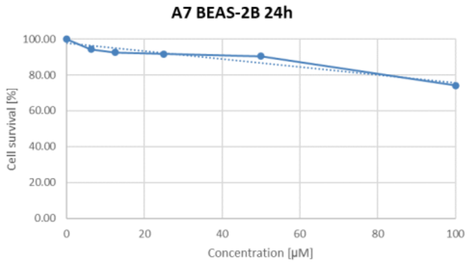

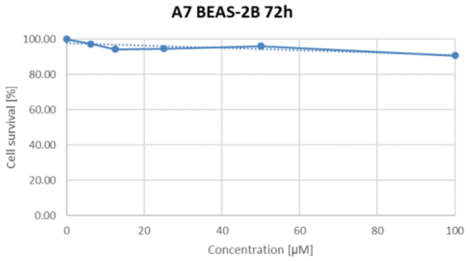

A8

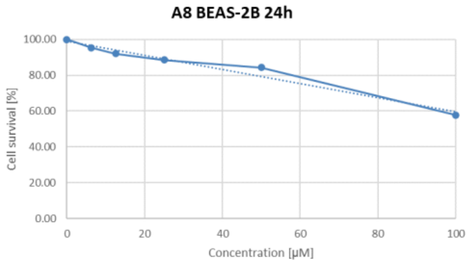

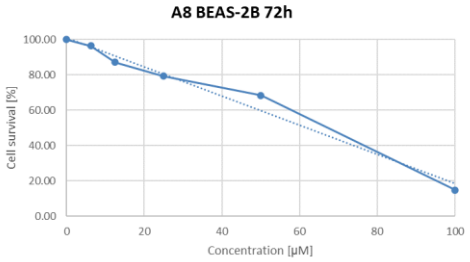

A9

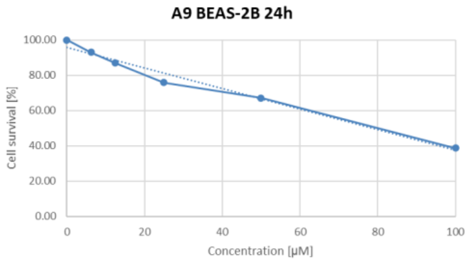

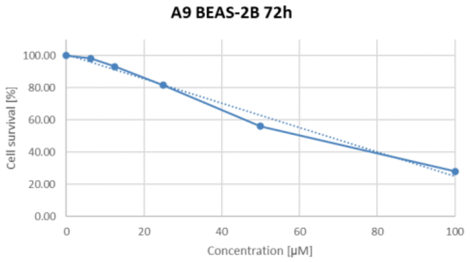

A10

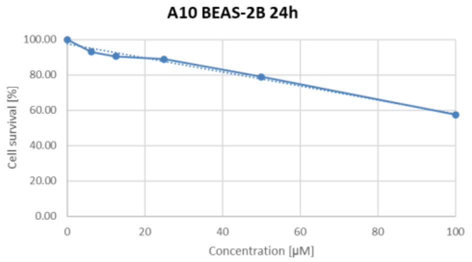

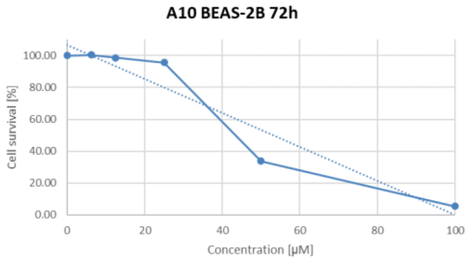

A11

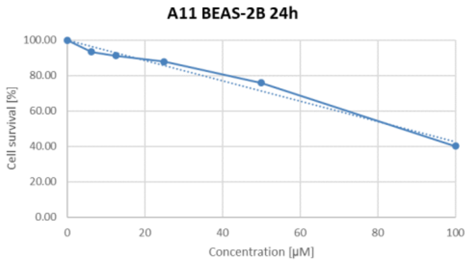

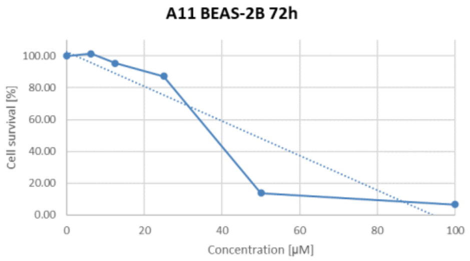

A12

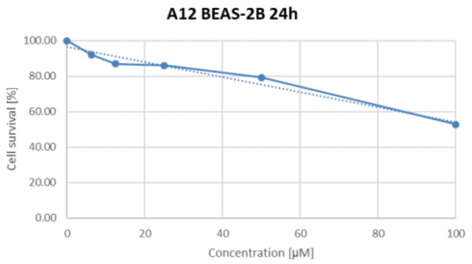

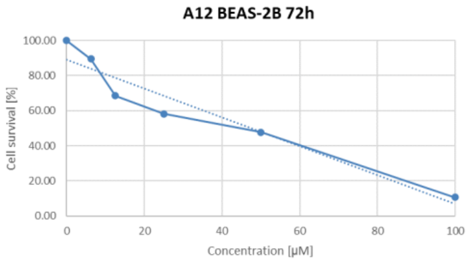

Figure S2. Representative microscopic images of HCT116 cells after 24 h treatment with compounds **A1–A12** at a concentration of 100 µM under standard culture conditions. Images were acquired at 10× magnification.

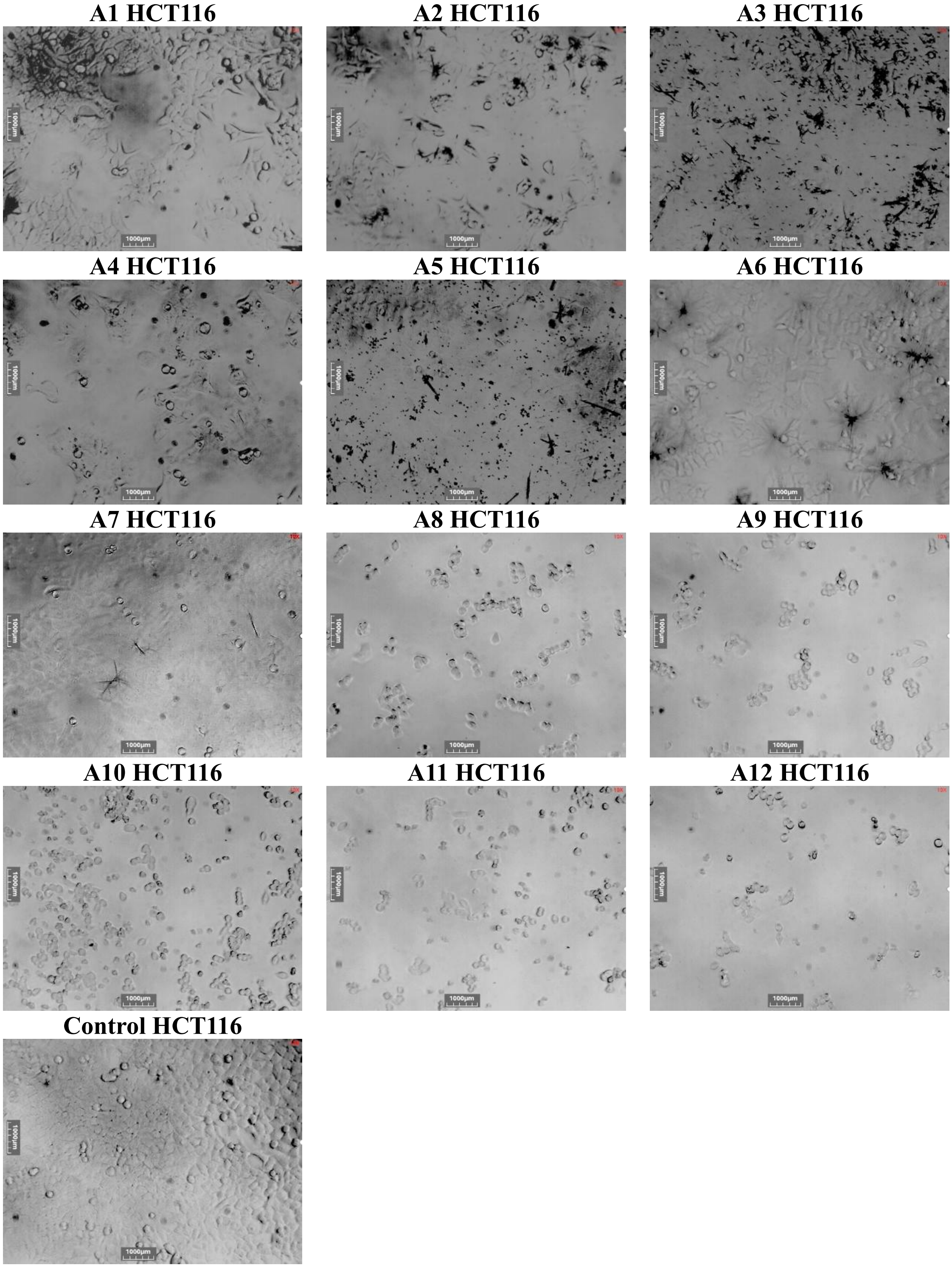

Figure S3. Representative microscopic images of HT29 cells after 24 h treatment with compounds **A1–A12** at a concentration of 100 µM under standard culture conditions. Images were acquired at 10× magnification.

A1 HT29

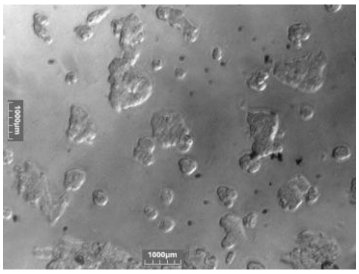

A2 HT29

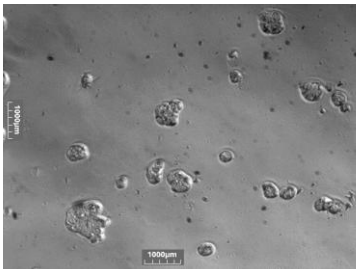

A3 HT29

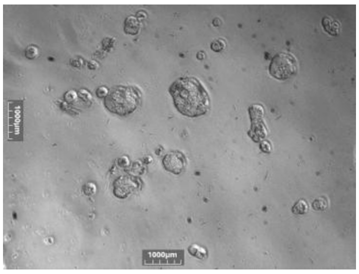

A4 HT29

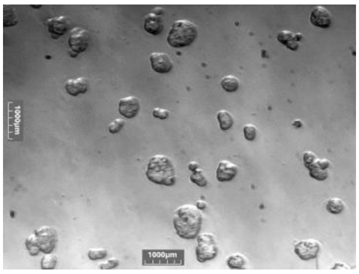

A5 HT29

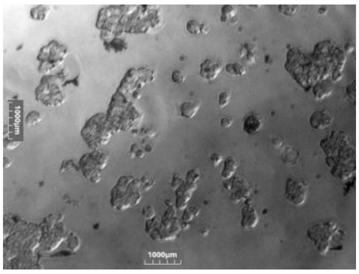

A6 HT29

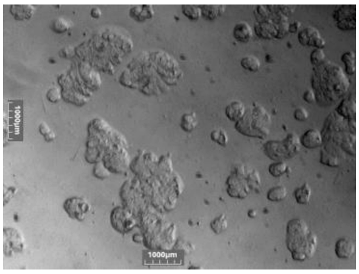

A7 HT29

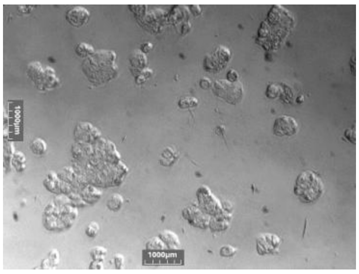

A8 HT29

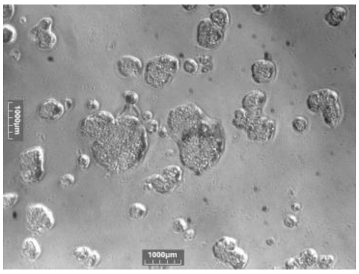

A9 HT29

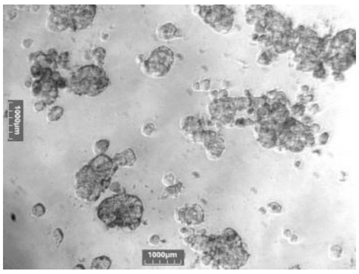

A10 HT29

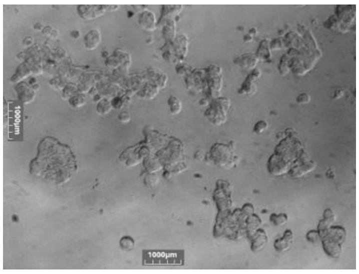

A11 HT29

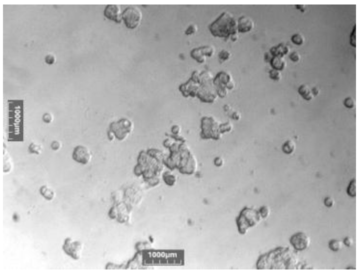

A12 HT29

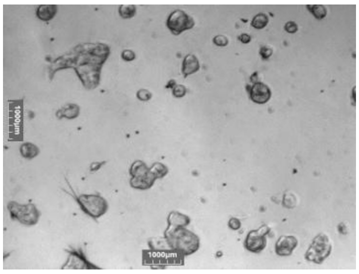

Control HT29

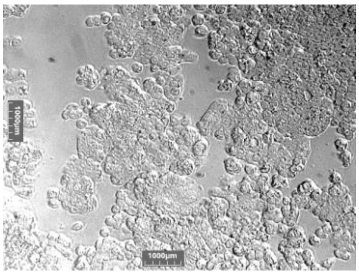

Figure S4. Representative microscopic images of BEAS-2B cells after 24 h treatment with compounds **A1–A12** at a concentration of 100 µM under standard culture conditions. Images were acquired at 10× magnification.

A1 BEAS-2B

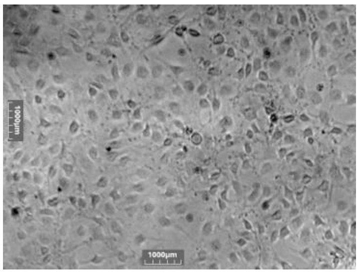

A2 BEAS-2B

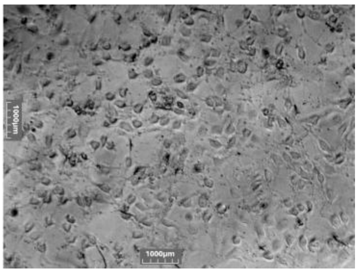

A3 BEAS-2B

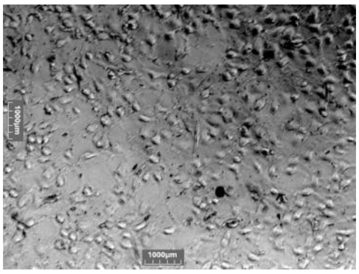

A4 BEAS-2B

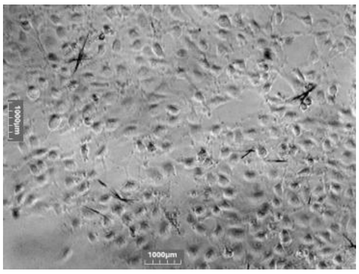

A5 BEAS-2B

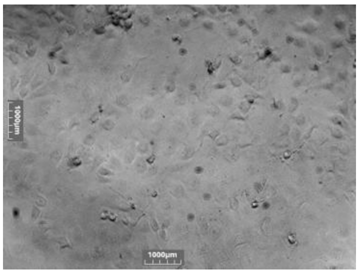

A6 BEAS-2B

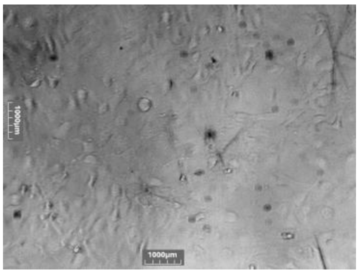

A7 BEAS-2B

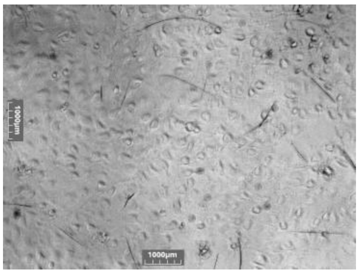

A8 BEAS-2B

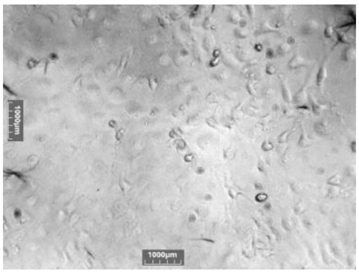

A9 BEAS-2B

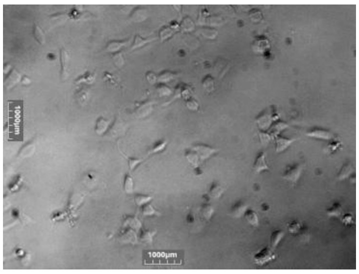

A10 BEAS-2B

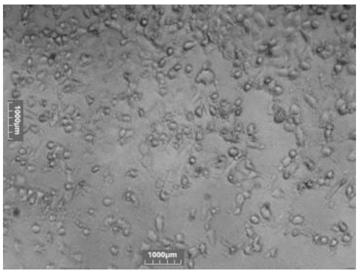

A11 BEAS-2B

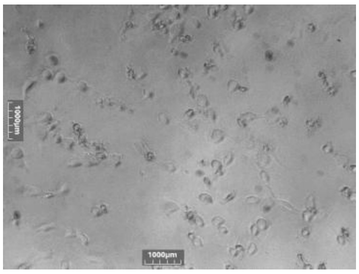

A12 BEAS-2B

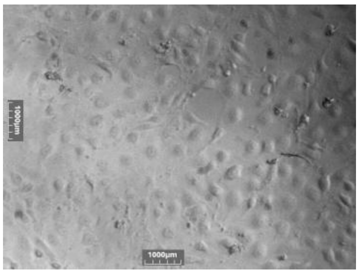

Control BEAS-2B

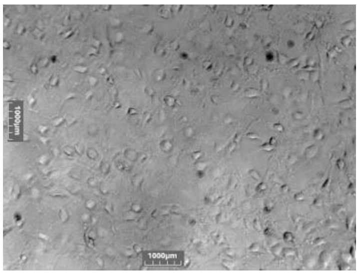

Figure S5. Representative flow cytometry histograms showing intracellular ROS levels in HCT116 and BEAS-2B cells following treatment with compounds **A1–A12** for 24 h. ROS production was assessed using CellROX™ Green staining.

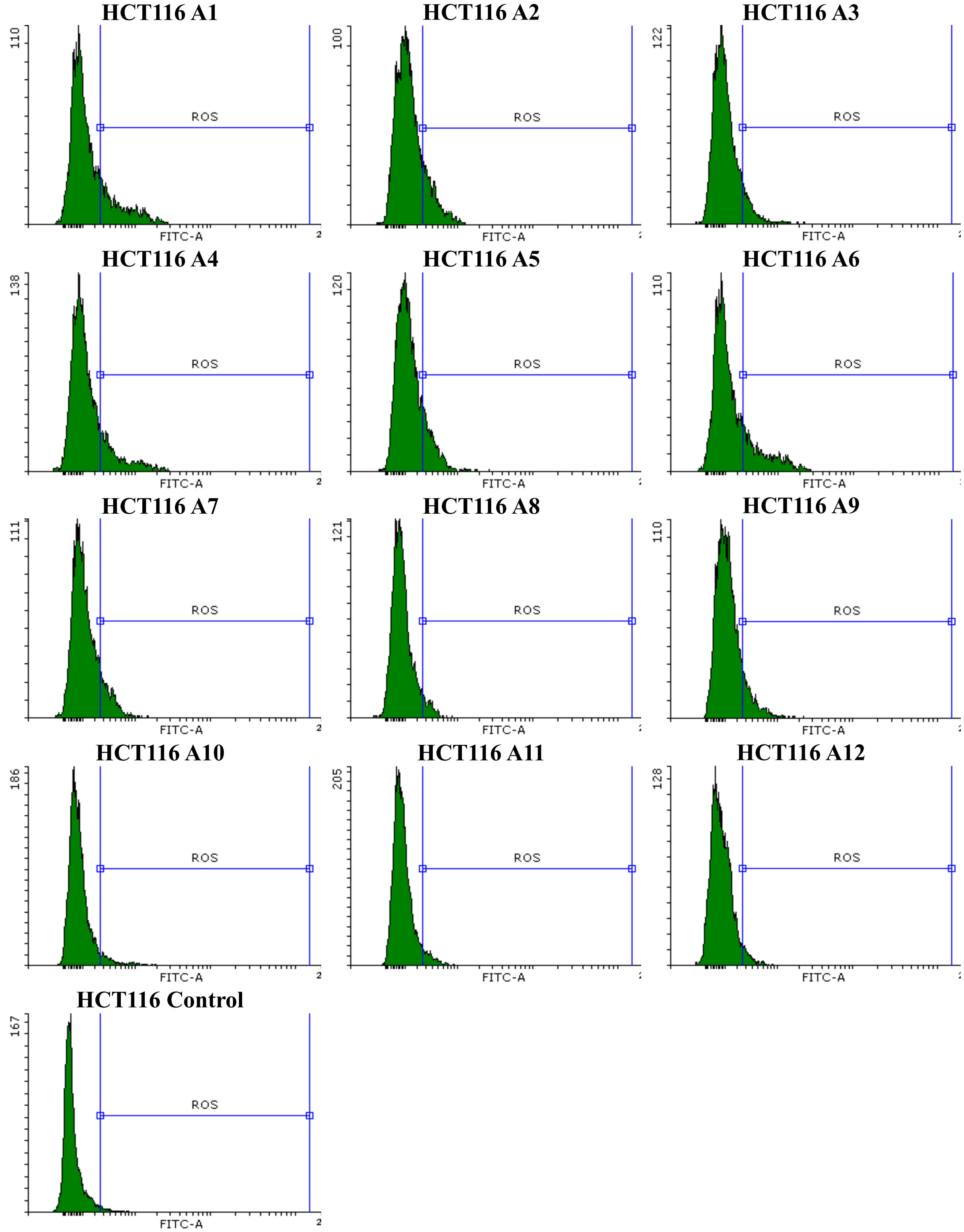

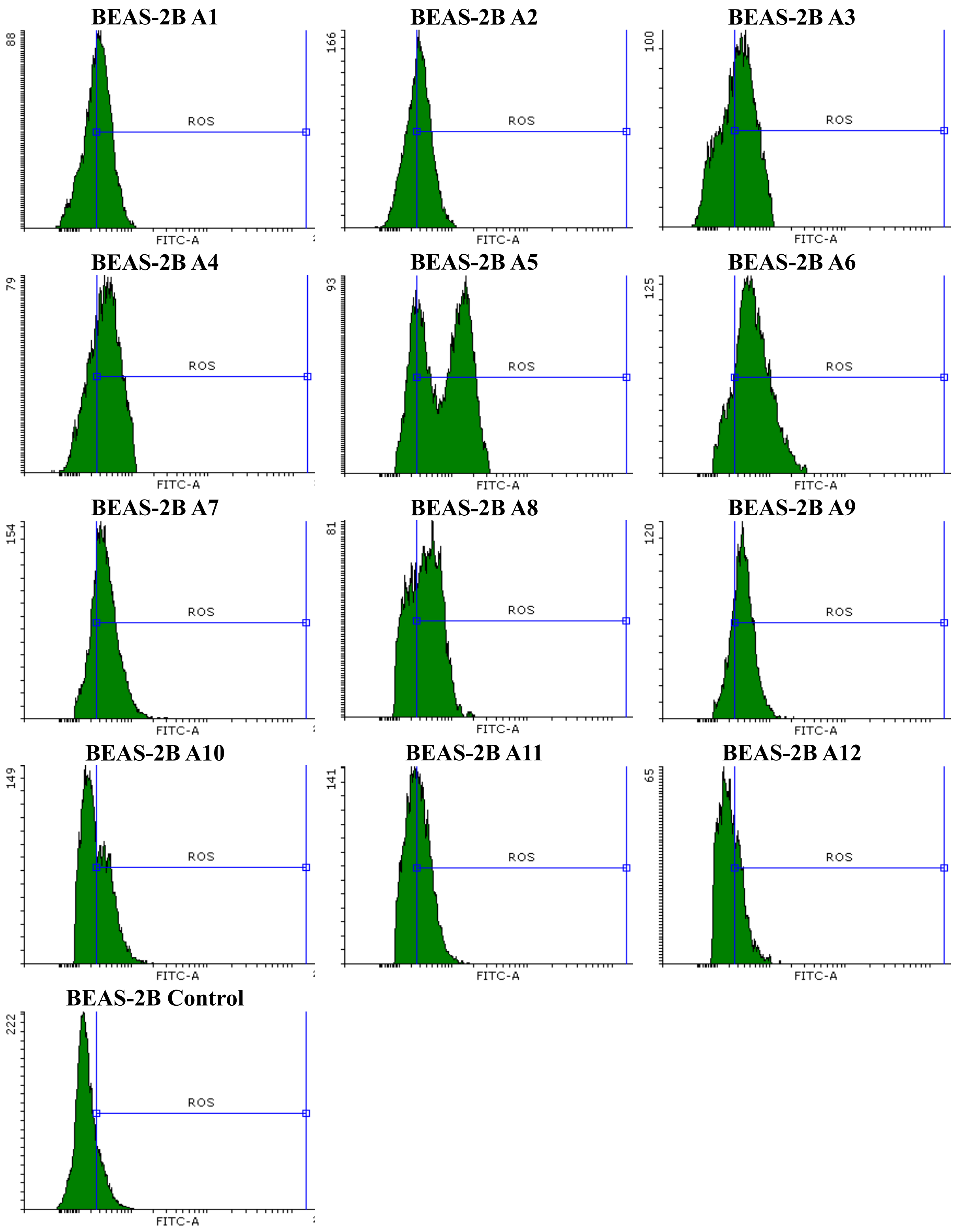
BEAS-2B A1
88
ROS
FITC-A
BEAS-2B A2
166
ROS
FITC-A
BEAS-2B A3
100
ROS
FITC-A
BEAS-2B A4
79
ROS
FITC-A
BEAS-2B A5
93
ROS
FITC-A
BEAS-2B A6
125
ROS
FITC-A
BEAS-2B A7
154
ROS
FITC-A
BEAS-2B A8
81
ROS
FITC-A
BEAS-2B A9
120
ROS
FITC-A
BEAS-2B A10
149
ROS
FITC-A
BEAS-2B A11
141
ROS
FITC-A
BEAS-2B A12
65
ROS
FITC-A
BEAS-2B Control
222
ROS
FITC-A

Figure S6. Quantitative cell cycle distribution of HCT116 cells after 24 h treatment with compounds **A1**–**A12** at a concentration of 100 µM. Data are presented as mean ± SD from three replicates.

Treatment	sub-G1 [%]	G0/G1 [%]	S [%]	G2/M [%]
Control	5.52±0.66	79.92±1.06	3.61±0.18	10.95±1.01
A1	18.15±0.92	58.35±1.31	5.48±1.12	18.03±0.65
A2	32.82±0.68	44.60±0.33	3.97±0.10	18.60±0.96
A3	9.29±0.48	72.48±0.77	2.70±0.23	15.53±1.03
A4	19.27±1.07	59.66±0.38	4.92±0.52	16.16±1.19
A5	13.62±0.43	21.66±0.83	11.13±0.43	53.59±0.49
A6	12.37±0.45	61.58±0.23	2.05±0.40	24.00±0.21
A7	8.58±0.37	76.83±0.67	2.09±0.07	12.50±0.38
A8	18.65±2.14	60.21±0.34	4.17±0.93	16.97±1.79
A9	11.54±0.44	32.81±0.77	20.22±0.29	35.42±0.73
A10	11.48±0.21	31.35±0.71	24.33±0.21	32.84±0.35
A11	19.46±0.76	25.40±0.71	19.15±0.28	35.98±0.46
A12	17.96±0.42	34.74±1.29	21.12±0.33	26.19±1.43

Figure S7. Quantitative cell cycle distribution of BEAS-2B cells after 24 h treatment with compounds **A1**–**A12** at a concentration of 100 µM. Data are presented as mean ± SD from three replicates.

Treatment	sub-G1 [%]	G0/G1 [%]	S [%]	G2/M [%]
Control	8.26±0.28	39.47±1.61	11.41±0.57	40.86±0.87
A1	9.01±1.89	44.39±0.63	19.13±1.15	27.46±2.43
A2	13.46±0.42	43.78±1.84	14.14±1.13	28.62±0.51
A3	5.08±0.35	46.16±0.33	13.76±0.74	35.01±0.72
A4	15.40±0.92	41.80±1.48	13.62±0.86	29.18±1.92
A5	12.98±1.53	41.55±1.08	21.51±0.67	23.96±1.50
A6	10.73±0.60	43.82±0.62	15.14±0.08	30.30±1.22
A7	16.46±0.56	43.06±0.51	13.12±0.91	27.36±0.56
A8	38.85±0.28	31.90±0.33	14.81±0.60	14.43±0.86
A9	51.69±1.23	22.62±0.85	18.32±0.37	7.36±0.15
A10	42.73±0.80	31.06±0.74	13.24±0.28	12.97±0.43
A11	53.68±2.09	22.86±1.09	13.95±1.35	9.50±1.49
A12	43.72±1.51	29.20±1.70	15.82±1.46	11.26±1.03

Figure S8. Representative Annexin V-FITC/PI dot plots of HCT116 and BEAS-2B cells treated with compounds **A1–A12** for 24 h. The plots illustrate the distribution of viable, early apoptotic, late apoptotic, and necrotic cell populations.

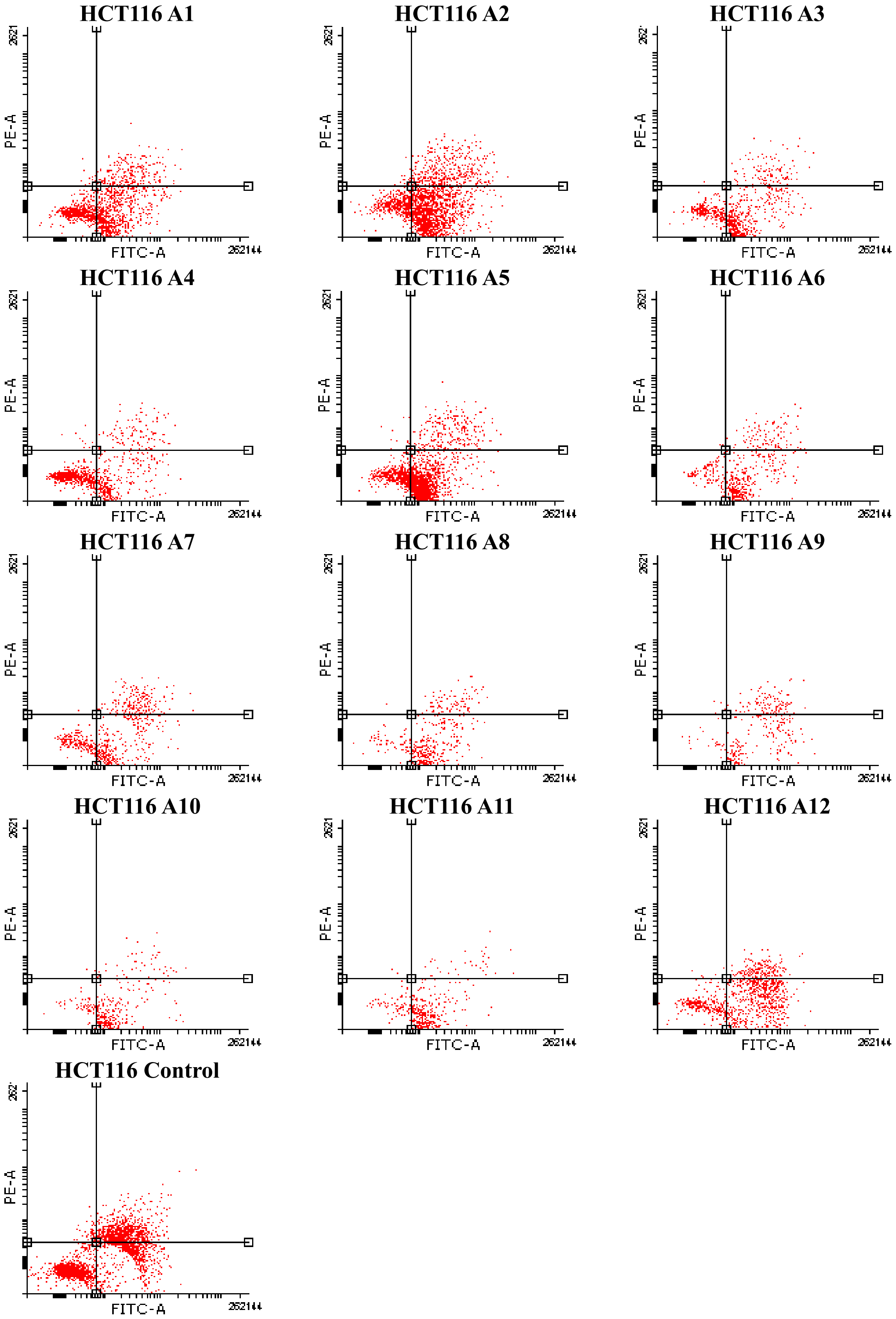

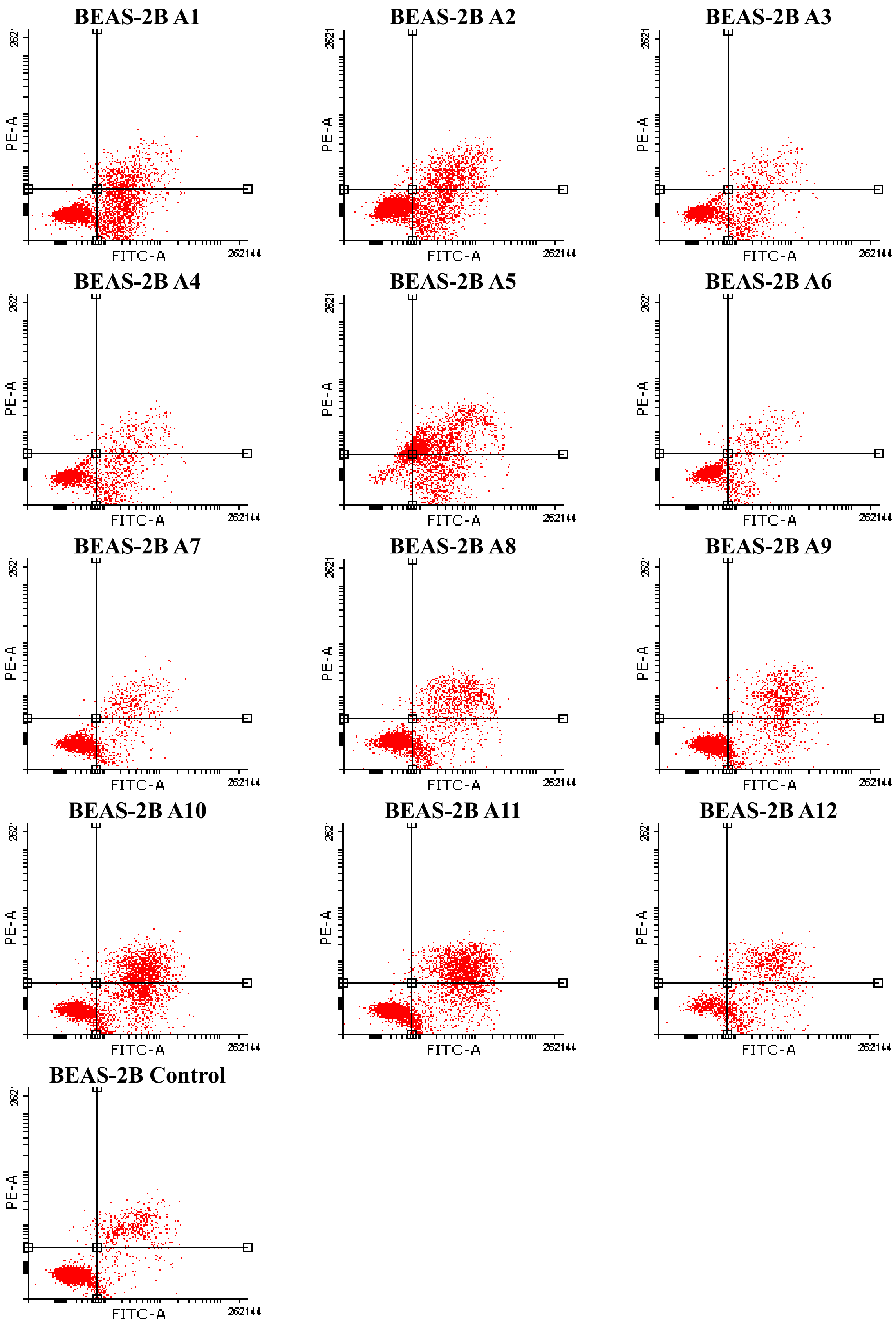
BEAS-2B A1
BEAS-2B A2
BEAS-2B A3
BEAS-2B A4
BEAS-2B A5
BEAS-2B A6
BEAS-2B A7
BEAS-2B A8
BEAS-2B A9
BEAS-2B A10
BEAS-2B A11
BEAS-2B A12
BEAS-2B Control
PE-A
FITC-A
262144
